# Flexible Bayesian Product Mixture Models for Vector Autoregressions

**Published:** 2024-04

**Authors:** Suprateek Kundu, Joshua Lukemire

**Affiliations:** Department of Biostatistics, The University of Texas MD Anderson Cancer Center, University of Texas, Houston, TX 77030, USA; Department of Biostatistics and Bioinformatics, Emory University, Atlanta, GA 30322, USA

**Keywords:** Dirichlet process mixtures, spatio-temporal data, functional magnetic resonance imaging, human connectome project, vector auto-regressive models

## Abstract

Bayesian non-parametric methods based on Dirichlet process mixtures have seen tremendous success in various domains and are appealing in being able to borrow information by clustering samples that share identical parameters. However, such methods can face hurdles in heterogeneous settings where objects are expected to cluster only along a subset of axes or where clusters of samples share only a subset of identical parameters. We overcome such limitations by developing a novel class of product of Dirichlet process location-scale mixtures that enables independent clustering at multiple scales, which results in varying levels of information sharing across samples. First, we develop the approach for independent multivariate data. Subsequently we generalize it to multivariate time-series data under the framework of multi-subject Vector Autoregressive (VAR) models that is our primary focus, which go beyond parametric single-subject VAR models. We establish posterior consistency and develop efficient posterior computation for implementation. Extensive numerical studies involving VAR models show distinct advantages over competing methods in terms of estimation, clustering, and feature selection accuracy. Our resting state fMRI analysis from the Human Connectome Project reveals biologically interpretable connectivity differences between distinct intelligence groups, while another air pollution application illustrates the superior forecasting accuracy compared to alternate methods.

## 1. Introduction

Multivariate time-series data routinely arise in diverse application areas such as finance (Cramer and Miller, 1978), econometrics (Engle and Watson, 1981), air pollution forecasting (Nath et al., 2021) and medical imaging (Kundu and Risk, 2021), among other domains. In order to tackle such data, a rich body of work on modeling autocorrelations and temporal cross-correlations between variables with multivariate outcomes has been developed, of which vector autoregressive (VAR) models are widely used (Lütkepohl, 2005). Our focus in this paper is on Bayesian VAR modeling, which was initially heavily motivated by econometric research (Doan et al., 1984) and has since seen a rich development (Korobilis, 2013). More recently, Bayesian VAR models have been adopted with increasing prominence in biomedical research including patient-level predictive modeling (Lu et al., 2018) and functional Magnetic Resonance Imaging (fMRI) applications (Gorrostieta et al., 2013; Chiang et al., 2017) in neuroimaging studies. However, existing Bayesian VAR literature has primarily focused on methodological and computational developments, with limited theoretical investigations. Recently, Ghosh et al. (2018) addressed this gap by establishing posterior consistency for the autocovariance matrix in parametric Bayesian VAR models based on single subject data.

The vast majority of the Bayesian VAR literature involves Gaussian assumptions and parametric prior specifications that may not be sufficiently flexible in characterizing the underlying probability distributions with non-regular features. For example, it is known that the nature of shocks in econometric analysis may not always be Gaussian (Weise, 1999). Similarly, flexible VAR modeling is necessary for analyzing heterogeneous multi-subject data in neuroimaging studies, where parametric VAR models may prove inadequate (see our Human Connectome Project (HCP) application in Section 6). Non-Gaussianity is also observed in air pollution data captured via sensors (Kim et al., 2013), where it is often of interest to perform forecasting using VAR models (Hajmohammadi and Heydecker, 2021). Such parametric VAR models may result in inaccurate performance when parametric assumptions are violated or even mis-specified. To bypass parametric constraints in VAR models, some recent articles relaxed Gaussianity assumptions (Jeliazkov, 2013). Recently, Bayesian nonparametric VAR models were proposed by Kalli and Griffin (2018) involving single subject data, where the mixing weights of the transition density depend on the previous lags. On the other hand, Billio et al. (2019) proposed Dirichlet process mixture of normal-Gamma priors on the VAR autocovariance elements. Unlike for the parametric case, the non-parametric methods are more robust to mis-specification and can potentially cater to a large class of models. However, the above approaches were applied to small or moderate dimensional data with limited or no emphasis on pooling information across samples and with negligible or no theoretical investigations.

Existing literature has largely ignored the problem of developing provably flexible non-parametric Bayesian VAR methodology to model heterogeneous multi-subject time-series data, to our knowledge. Such approaches are desirable over single-subject VAR analyses in terms of being able to pool information across samples in a flexible manner that can accommodate arbitrary probability distributions. They also facilitate robust and reproducible parameter estimates and provide a natural foundation for conduct inferences to test for differences across samples via credible intervals, which may not be straightforward under single-subject analysis. Although there is some literature on parametric VAR modeling of multi-subject data, these existing approaches typically require *a priori* knowledge of class labels (Gorrostieta et al., 2013; Chiang et al., 2017; Kook et al., 2021). Hence, they have a limited ability to accommodate heterogeneity within each class and may result in poor performance when the class labels are mis-specified due to no clear distinction between groups. Moreover, they clearly suffer from the aforementioned pitfalls of parametric methods.

Motivated by the above discussions, we propose a broad class of novel Bayesian non-parametric models that specify Dirichlet process (DP) mixture priors independently on mutually exclusive subsets of model parameters. Our specification results in a product of Dirichlet process mixture (PDPM) priors. A key feature of the proposed approach is the ability to allow differential clustering at multiple scales, which enables clusters of samples that share only a subset of common model parameters resulting in greater flexibility. We develop several variants of the proposed approach that encourage differential degrees of heterogeneity via different modes of multiscale clustering by altering the manner in which the parameter space is partitioned. First, we develop the PDPM approach in the generic setting of multivariate density estimation for kernel mixtures of the form ∫K(x;Θ)dP(Θ), and establish posterior consistency properties. We also provide a toy example that illustrates the distinct numerical advantages of the product mixture models compared to traditional DP mixtures in terms of clustering accuracy. Subsequently, we generalize the proposed PDPM approach to multivariate time-series data under the framework of a VAR model, which is our primary focus in this article. In such settings, the multiscale clustering approach becomes even more relevant given the large number of parameters in the autocovariance matrix whose dimension grows quadratically with the vector dimension. Starting from a VAR model that allows for limited differences in clustering across multiple scales and greater model parsimony, we eventually develop a variant that is able to independently cluster row-specific parameters, which provides greater flexibility in practical applications. By specifying appropriate base measures in the DP prior, it is possible to enable appropriate shrinkage for the autocovariance elements that facilitates feature selection. Additional dimension reduction is also possible via a low rank representation for the residual covariance.

By designing non-parametric Bayesian VAR models based on heterogeneous multi-subject data, we are able to relax the parametric assumptions and provide a more flexible characterisation of heterogeneity via unsupervised clustering. The proposed methods are particularly desirable in terms of being able to bypass any restrictive assumptions such as the presence of replicated samples, which is routinely assumed in Bayesian non-parametric literature (Tokdar, 2006; Durante et al., 2017), but may potentially lead to inadequate characterization of heterogeneity. In particular, replicated samples are structured to share fully identical sets of model parameters within a given cluster, which may not be realistic in applications where heterogeneous samples are often effectively clustered only along a subset of directions with the remaining axes being uninformative/redundant for clustering (Agrawal et al., 2005).

Another appealing feature of the proposed approach is the associated posterior consistency properties for density estimation, as the number of samples (n) grows to ∞. We note that such theoretical results for VAR models involving multivariate time-series data represent non-trivial extensions of the rich theoretical properties established in the Bayesian non-parametric literature for independent outcomes (Tokdar, 2006; Canale and De Blasi, 2017). We resolve the significant challenges arising from the non-parametric Bayesian theoretical analysis by establishing Kullback-Leibler properties for VAR models, and constructing carefully designed sieves that are shown to satisfy certain entropy bounds and tail prior probability conditions under the product of DP priors. Moreover, we show that the theoretical results hold for commonly used base measures that enable straightforward posterior computation, and subsequently outline the computational complexity.

We develop an efficient and scalable Markov chain Monte Carlo (MCMC) implementation for the proposed class of models in the VAR framework. In addition, we illustrate the sharp numerical advantages and efficient mixing under the proposed non-parametric Bayesian VAR approach in terms of parameter estimation and recovering the true clusters, compared to competing state-of-the-art methods. Further, the inferential capability of the proposed approach is evident from accurate feature selection of the autocovariance elements. Our analysis of resting state fMRI data from a subset of individuals in the HCP study infers several effective connectivity differences between the high and low fluid intelligence groups that are supported by existing evidence in literature. Moreover, the analysis under the proposed approach produces biologically reproducible estimates that are consistent across repeated neuroimaging scans from the same samples. In contrast, a single subject VAR analysis is able to identify only negligible effective connectivity differences across groups, which seems biologically implausible. Using a second data application example involving air pollution data from the Environment Protection Agency (EPA), we illustrate the considerable advantages in forecasting accuracy under the proposed approach compared to a parametric VAR model even when the dimension of the outcome is small.

The rest of the article is structured as follows. Section 2 develops the product of DP mixtures for independently distributed multivariate data and establishes posterior consistency properties. Section 3 extends the methodology to multivariate time-series data under a VAR framework, along with illustrating theoretical properties. Section 4 describes the posterior computation scheme. Section 5 reports results from extensive simulation studies involving VAR models. Sections 6 and 7 describe our analysis of the neuroimaging data from the HCP as well as air pollution data from the EPA. Section 8 contains additional discussions. Appendices are provided that contain other relevant details.

## 2. Product of DP Mixtures for Multivariate Data

### 2.1 A Primer on DP mixture approaches

Consider i.i.d. random vectors $x_{i},i,=1,\ldots ,n$, each of dimension D×1, and denote the collection of vectors as $X_{n}=\left\{x_{1},\ldots ,x_{n}\right\}$. Non-parametric Bayesian literature has often focused on modeling these vectors under a DP location mixture or location-scale framework. Such approaches (Escobar and West, 1995) often specify $x_{i}∼N\left(\mu_{i},\Sigma_{i}\right),\left(\mu_{i},\Sigma_{i}\right)∼P,P∼DP\left(MP_{0}\right),i=1,\ldots ,n$, where $\Sigma_{i}\in S_{D\times D}$ denotes the covariance for subject $i,S_{D\times D}$ denotes the space of all D×D symmetric positive definite matrices, $P_{0}$ denotes the base measure of the DP, and M is the precision parameter. We note that alternative choices other than the Gaussian kernel may also be used but are not considered here for simplicity. The resulting DP location-scale mixture induces the unknown probability density $f_{P}(x)=\int \;\phi_{\Sigma }(x-\mu )dP(\mu ,\Sigma )$, where $\phi_{\Sigma }(\cdot -\mu )$ denotes the density of a D-dimensional normal distribution with mean μ and covariance Σ. Given that $P∼DP\left(MP_{0}\right)$, the proposed method results in probability distributions on the class of densities $ℱ=\left\{f_{P}\right\}$, which can also be seen from the result $f_{P}(x)=\sum_{h=1}^{\infty }\pi_{h}\phi_{\Sigma_{h}}\left(x-\mu_{h}\right)$, where $\left(\mu_{h},\Sigma_{h}\right)∼P_{0}$ and $\pi_{h}=\nu_{h}\prod_{l=1}^{h-1}\left(1-\nu_{l}\right),\nu_{h}∼Be(1,M)$, using Sethuraman’s (1994) stick-breaking representation.

The above commonly used DP mixture specification results in clusters of replicated samples that share identical sets of parameters (μ,Σ), which allows for pooling of information across samples resulting in robust learning. While such a clustering mechanism is routinely used and often backed by posterior consistency guarantees, it may not be well-equipped to succeed for more heterogeneous settings where the clustering is dictated by a small number of axes or subspaces, with the other axes being irrelevant to clustering. A more reasonable approach is to allow differential clustering at multiple scales that does not constrain samples to share fully identical parameter sets, but instead allows subsets of parameters to cluster independently resulting in partially overlapping clusters. Such a multi-scale clustering approach results in a more accurate characterization of heterogeneity that is expected to improve finite sample performance. The above arguments form the basis of the proposed product mixture priors in this article.

### 2.2 Proposed Methodology and Properties

We propose a class of novel product mixture priors that is equipped to perform differential clustering at multiple scales. Consider equally sized mutually exclusive and exhaustive subsets of the full parameter set denoted as $\mu =\cup_{m_{1}=1}^{{ℳ}_{\mu }}\mu_{m_{1}},\tilde{\sigma }=\cup_{m_{2}=1}^{{ℳ}_{\sigma }}\sigma_{m_{2}}$, where $\tilde{\sigma }$ denotes the vectorized upper triangular matrix of Σ, and $\left\{\mu_{1},\ldots ,\mu_{{ℳ}_{\mu }}\right\}$ represent subsets of equal cardinality, and similarly for $\left\{\sigma_{1},\ldots ,\sigma_{{ℳ}_{\sigma }}\right\}$. Consider specifying the following product of DP priors on the parameters:

(1) $$\mu_{m_{1}}\overset{\mathrm{indep}}{∼}P_{\mu },\;m_{1}=1,\ldots ,{ℳ}_{\mu },\;\sigma_{m_{2}}\overset{\mathrm{indep}}{∼}P_{\sigma },\;m_{2}=1,\ldots ,{ℳ}_{\sigma },\;\text{Σ}\in S_{D\times D},\;P_{\mu }∼DP\left(\alpha_{1}P_{1}^{\text{*}}\right),\;P_{\sigma }∼DP\left(\alpha_{2}P_{2}^{\text{*}}\right),$$

where each component is assigned independent priors $\mu_{m_{1}}\overset{\mathrm{indep}}{∼}P_{\mu },\sigma_{m_{2}}\overset{\mathrm{indep}}{∼}P_{\sigma }$, that follow Dirichlet process with base measures $P_{1}^{*}$ and $P_{2}^{*}$ respectively, with corresponding precision parameters $\alpha_{1},\alpha_{2}$. The specification (1) results in a product of DP priors on the original parameters (μ,Σ) that is denoted by $\Pi^{*}$, where the exact prior depends on the way in which the partitions are defined. Hence, one can obtain a class of product mixture priors by tweaking the partition structure to reflect the most appropriate setting for the data at hand. The product priors in (1) induce priors Π on ℱ via the relationship:

(2) $$f_{P}(x)=\int \;\int \phi_{\text{Σ}}(x-\mu )d{\text{Π}}^{\text{*}}(\mu ,\text{Σ})\;=\sum_{h_{11},\ldots ,h_{1{ℳ}_{\mu }}=1}^{\infty }\sum_{h_{\sigma }=1}^{\infty }\pi_{1,h_{11}}\ldots \pi_{{ℳ}_{\mu },h_{1{ℳ}_{\mu }}}\pi_{\sigma ,h_{\sigma }}\phi_{{\text{Σ}}_{h_{\sigma }}}\left(x-{\left(\mu_{1,h_{11}}^{T},\ldots ,\mu_{{ℳ}_{\mu },h_{1{ℳ}_{\mu }}}^{T}\right)}^{T}\right),$$

where the second equality is obtained by Sethuraman’s (1994) stick breaking representation with $\pi_{h_{1m_{1}}}=\nu_{h_{1m_{1}}}\;\prod_{l_{1}<h_{1m_{1}}}\left(1-\nu_{l_{1}}\right),\nu_{l_{1}}∼Be\left(1,\alpha_{1}\right),\pi_{\sigma ,h_{\sigma }}=\nu_{\sigma ,h_{\sigma }}\prod_{l_{2}<h_{\sigma }}\left(1-\nu_{\sigma ,l_{2}}\right)$, $\nu_{\sigma ,h_{\sigma }}∼\mathrm{Be}\left(1,\alpha_{2}\right)$, and further $\Sigma_{h_{\sigma }}∼P_{2}^{*},\mu_{m_{1}}∼P_{1}^{*}$, and ${ℳ}_{\sigma }$ is assumed to be one in the above expression. The choice of ${ℳ}_{\sigma }=1$ is guided by practical considerations in VAR models that is our primary focus (next section) where the residual covariance matrix often has a sparse or even diagonal structure after regressing out the lag effects of previous time points. However our treatment can be generalized to ${ℳ}_{\sigma }>1$ in a straightforward manner. We note that the above form in (2) follows the generic kernel mixture representation ∫K(x;Θ)dP(Θ) that is commonly considered in non-parametric Bayesian density estimation literature (Wu and Ghosal, 2008). We denote the resulting class of priors on ℱ arising from (2) as the product of Dirichlet process mixtures (PDPM).

**Figure 1:**
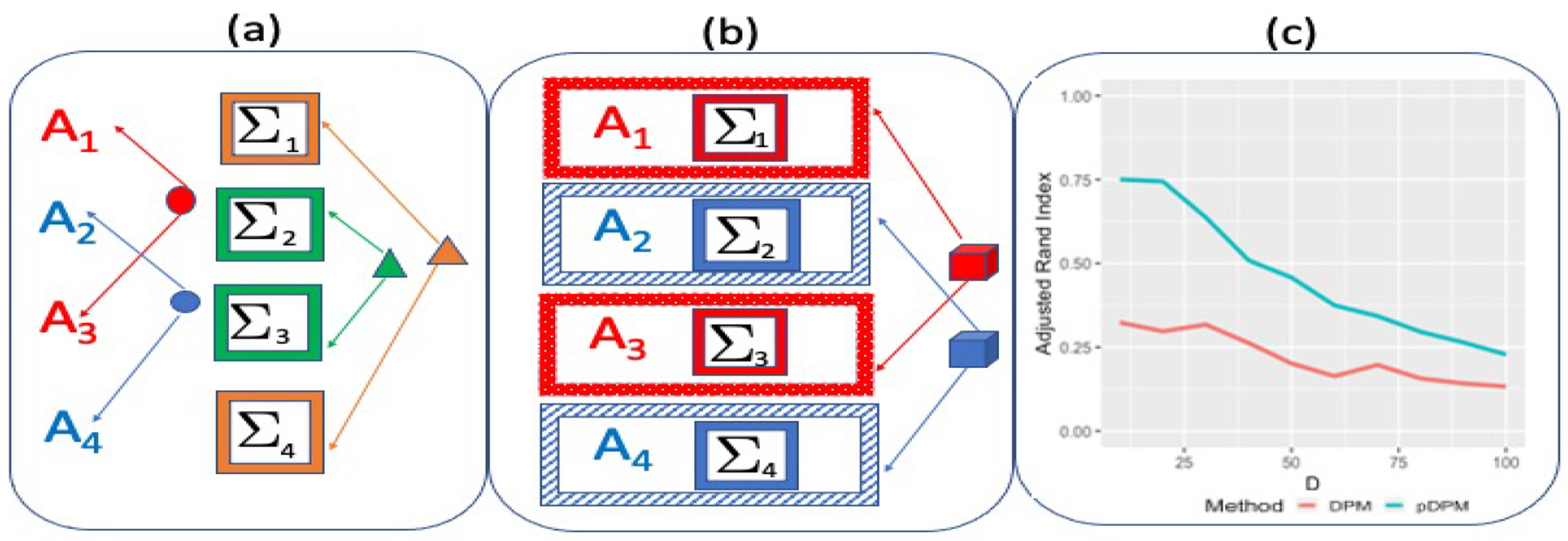
A schematic representation of the product of DP mixture prior. Panel (a) illustrates the product prior that separately clusters the mean (represented by A) into red and blue clusters and the covariance (Σ) into green and saffron clusters. Panel (b) represents the traditional DP mixture prior that forms clusters containing samples having identical values for both the mean and covariance parameters. Panel (c) illustrates the results from the toy example under the traditional DPM and the proposed PDPM, in terms of the change in clustering accuracy with varying dimension when the clustering is dictated by a subset of axes.

The most straightforward case of the prior in (1) is given as $\Pi^{*}(\mu ,\Sigma )=P_{\mu }(\mu )\times P_{\sigma }(\Sigma )$, which specifies independent priors on the mean and covariance parameters without further partitioning these parameters (i.e. ${ℳ}_{\mu }=1,{ℳ}_{\sigma }=1$). The PDPM operates by clustering the mean and covariance parameters independently, which suggests separate modes of pooling information across samples for the mean and covariance. Such a multiscale clustering approach results in greater flexibility and a more accurate characterization of heterogeneity compared to replicated samples with fully identical parameter sets via allowing for dedicated clusters of samples that share common mean signatures (but not necessarily for the covariance), along with independently constructed subgroups of samples that share common patterns in the covariance (but not necessarily for the mean). As a more flexible generalization, one can consider the *generalized PDPM (gPDPM)* model that specifies independent DP priors for each element of the mean vector, i.e. $\Pi^{*}(\mu ,\Sigma )=\prod_{m=1}^{D}P_{\mu }\left(\mu_{m}\right)\times P_{\sigma }(\tilde{\sigma })$. The gPDPM approach allows separate clustering for each element of μ across samples, which enables differential clustering along various axis and provides a more granular approach for pooling information, albeit at the cost of a larger number of model parameters . The multiscale clustering approach is expected to excel in settings where the clustering is dictated by a subset of axes in the mean with the other axes being redundant towards clustering. The above discussions highlight the advantages of the multiscale clustering aspect under the proposed product mixture modeling methodology, and provides the central motivation for this article. A schematic representation of the above ideas is presented in Figure 1.

#### Toy Example:

We illustrate the advantages of the multiscale clustering approach using a toy example. Multivariate data $Y_{i}∼N_{D}\left(\mu_{i},\Sigma_{i}\right)$, for i=1,…,250, was generated such that the mean across samples were identical except the first d elements, where $d\approx \frac{D}{3}$. For the first d elements of μ, there were 5 clusters, each with a corresponding d–vector of μ values. Similarly, 5 clusters were generated for Σ(D×D) that were constructed independent of the mean. We used the standard DPM and the proposed PDPM to fit these data, and evaluate the clustering performance across varying dimensions. The posterior computation steps for both approaches are just simplified versions of the posterior computation for the PDPM-VAR model that will be introduced in the sequel (omitted here for conciseness). For each method, we evaluated the adjusted Rand index for clustering the mean vector that is averaged over all MCMC iterations (Rand, 1971). Figure 1c illustrates the clustering accuracy. Unsurprisingly, the PDPM offers significant improvement over the DPM for such a heterogeneous clustering setup across varying dimensions D, which clearly illustrates the considerable advantages of the proposed approach. The standard DPM approach allocates identical mean and covariance parameters for all samples within each cluster, which can not tackle the differential clustering allocations between the mean and covariance and hence results in spurious clusters that adversely affect the overall clustering accuracy.

#### Theoretical Properties:

From a theoretical perspective, it is possible to show that the proposed product of DP approach leads to posterior consistency for Kullback-Leibler neighborhoods, under reasonable assumptions on the true density $f_{0}$ that are routinely assumed in multivariate density estimation literature (Wu and Ghosal, 2008). This is not surprising given that the density follows a generic kernel mixture representation ∫K(x;Θ)dP(Θ) commonly encountered in the literature. Some additional notations are provided below. Denote the Euclidean norm for a vector as ‖⋅‖, and denote the spectral norm of a matrix as $\|\cdot {\|}_{2}$. Further, denote the eigenvalues of a D×D positive definite matrix Σ in decreasing order as $\lambda_{1}(\Sigma )\geq \ldots \geq \lambda_{D}(\Sigma )$. Let a≲b imply that a is less than b upto a constant, and let $\left\lfloor \cdot \right\rfloor$ denote the floor operator. Denote the Kullback-Leibler (KL) divergence between densities f,g∈ℱ as KL(f,g)=∫log(f/g)f. Denote the set of natural numbers as ℕ.

We now establish our result on positive prior support for Kullback-Leibler neighborhoods under the product of DP prior below, based on the following assumptions.

*(C1)* $0<f_{0}(x)<M$ for some constant M and all $x\in {ℜ}^{D}$;

*(C2)* $\int \;f_{0}(x)log\left(f_{0}(x)\right)dx<\infty$;

*(C3)* $\int \;f_{0}(x)log\left(f_{0}(x)/\phi_{\delta }(x)\right)dx<\infty$ where $\phi_{\delta }(x)={inf}_{\|t-x\|<\delta }f_{0}(t)$ for some δ>0;

*(C4)* for some $\eta >0,\int \;\|x{\|}^{2(1+\eta )}f_{0}(x)dx<\infty$.

Assumptions (C1)-(C4) are similar to routinely used conditions in non-parametric Bayesian literature for establishing posterior consistency properties. For example, these conditions were proposed in Wu and Ghosal (2008) for establishing Kullback-Leibler convergence properties for location-scale mixtures. Since then, they have been used extensively in related literature such as Wu and Ghosal (2010) for multivariate location mixtures, in Canale and De Blasi (2017) for showing strong consistency properties for multivariate location-scale mixtures, as well as for conditional density estimation (Pati et al., 2013), and Dirichlet mixtures of exponential power densities (Scricciolo, 2011), among others. Condition (C1) simply implies that the true density $f_{0}$ is bounded which is a reasonable and mild assumption. Conditions (C2) and (C3) are subtle, but are also mild, as noted in Pati et al. (2013). For example, condition (C2) should be satisfied by appropriate location-scale mixtures of normals. Condition (C4) imposes a minor tail restriction that should be satisfied by the t-distribution with suitable degrees of freedom, among others.

**Lemma 1:** *Let* $f_{0}\in ℱ$ *and assume conditions (C1)-(C4) hold. Then for the prior defined in* (1), *we have* $\Pi \left(f\in ℱ\;:\;\int log\left(f_{0}/f\right)f_{0}\leq \eta^{*}\right)\geq 0$, *for any* $\eta^{*}>0$.

**Remark 1:** Lemma 1 provides weak consistency guarantees by establishing positive prior support for arbitrarily small Kullback-Leibler neighborhoods of $f_{0}$, as per Schwartz (1965).

An outline of the proof is provided in the Appendix. Although weak consistency is useful, a more appealing feature is strong consistency, ensuring that the posterior distribution concentrates in arbitrarily small $L_{1}$ neighborhoods of the true density. The next result states that under certain tail conditions on the base measure of the DP priors, it is possible to derive strong posterior consistency corresponding to the PDPM priors.

**Theorem 1:** *Suppose* $f_{0}$ *satisfies the conditions of Lemma 1. Then the posterior is strongly consistent at* $f_{0}$ *under the PDPM and gPDPM priors* Π *in* (2) *with base measures that satisfy the conditions: (i)* $P_{2}^{*}\left(\lambda_{1}\left(\Sigma_{h_{\sigma }}^{-1}\right)>x^{*}\right)≲exp\left(-c_{1}{\left(x^{*}\right)}^{c_{2}}\right),P_{2}^{*}\left(\lambda_{D}\left(\Sigma_{h_{\sigma }}^{-1}\right)\;<\;1/x^{*}\right)≲{\left(x^{*}\right)}^{-c_{3}},P_{2}^{*}\left(\frac{\lambda_{1}\left(\Sigma^{-1}\right)}{\lambda_{D}\left(\Sigma_{h_{\sigma }}^{-1}\right)}>x^{*}\right)≲{\left(x^{*}\right)}^{-\kappa }$, *for some constants* $c_{1},c_{2},c_{3},\kappa$, *and all clusters* $h_{\sigma }$; *(ii)* $P_{1}^{*}\left(\left\|\mu_{m_{1},h_{1m_{1}}}\right\|>x^{*}\right)≲{\left(x^{*}\right)}^{-2(r+1)}$ *for all clusters* $h_{1m_{1}}$, *where* $m_{1}=1,\ldots ,{ℳ}_{\mu }$.

Theorem 1 provides explicit conditions on the PDPM prior that will ensure strong consistency, corresponding to any true density $f_{0}$ lying in the weak neighborhood of the prior Π on the set of densities ℱ. The proof is provided in the Appendix. The tail conditions on the base measures in Theorem 1 are very reasonable and hold for commonly used distributions (such as Gaussian and Laplace) on the mean, as well as the inverse-Wishart distribution on the covariance (see Lemmas 2-3 in the sequel). It should be noted that the procedure for proving the strong consistency result in Theorem 1 corresponding to non-compact space of densities ℱ relies on carefully designed sieves ${ℱ}_{n}$ that are compact subsets of ℱ but that grow with n to eventually cover all of ℱ as n→∞. These sieves must satisfy certain sufficient conditions for the strong consistency result to hold. These sufficient conditions are motivated from ideas in Theorem 5 of Ghosal and Van Der Vaart (2007), and were derived by Shen et al. (2013) for location mixtures and in Theorem 1 of Canale and De Blasi (2017) for location-scale mixtures. For clarity, we restate the result in Canale and De Blasi (2017) as Theorem 2 in our paper that will be leveraged to establish the posterior consistency under our set-ups in Sections 2 and 3 (Theorems 1 and 5 respectively).

Denote the entropy of a space of densities 𝒢⊂ℱ as N(ϵ,𝒢,d), which is defined (in terms of the metric d) as the minimum integer N for which there exists densities $f_{1},\ldots ,f_{N}\in ℱ$ satisfying $𝒢\subset \cup_{j=1}^{N}\left\{f\;:\;d\left(f,f_{j}\right)<\epsilon \right\}$. The distance metric used to study convergence in the space ℱ is evaluated in terms of the Hellinger distance (defined $d(f,g)={\left[\int (\sqrt{f}-\sqrt{g}{)}^{2}\right]}^{1/2}$), as well as the $L_{1}$ metric (defined as $\left\|f-g{\|}_{1}=\int |f-g\right|$). Further, denote $F_{0}^{n}$ as the n-products measure, where $F_{0}$ is the probability measure corresponding to $f_{0}$.

**Theorem 2 (Canale and De Blasi):** *Consider sieves* ${ℱ}_{n}\subset ℱ$ *with* ${ℱ}_{n}↑ℱ$ *as* n→∞, *where* ${ℱ}_{n}=\cup_{j}{ℱ}_{n,j}$, *with (2A)* $\Pi \left({ℱ}_{n}^{c}\right)≲e^{-bn}$; *and (2B)* $\sum_{j}\sqrt{N\left(2\epsilon ,{ℱ}_{n,j},d\right)}\sqrt{\Pi \left({ℱ}_{n,j}\right)}e^{-(4-c)n\epsilon^{2}}\to 0$, *for* b,c,ϵ>0. *Then* $\Pi \left(f\;:\;d\left(f_{0},f\right)>8\epsilon |X_{n}\right)\to 0$ *in* $F_{0}^{n}$–*probability for any* $f_{0}$ *in the weak support of* Π *defined in* (1).

In Theorem 2, condition (*2A*) suggests that the sieve should grow with the sample size such that only small neighborhoods with exponentially small prior probabilities are excluded. On the other hand, condition (*2B*) reflects the summability condition that involves smaller subsets ${ℱ}_{n,j}$ that cover the sieve ${ℱ}_{n}$ under the union operation. It places constraints on the growth rate of the metric entropy in a manner that the weighted sum of the square root of metric entropy of ${ℱ}_{n,j}$ (weighted by the corresponding square root of prior probabilities) go towards zero with increasing n. We construct such sieves in the proof of Theorem 1 in the Appendix, and illustrate that the conditions (2A) and (2B) are satisfied, which results in strong consistency.

Model (1) lays the foundation for the novel PDPM priors, that potentially has a wide array of applications, and can likely be generalized to most frameworks that involve clustering under Dirichlet process mixtures. We are now well positioned to turn our focus on the primary goal in this article, which is to develop a provably flexible non-parametric Bayesian methodology for multivariate time-series data modeled under a VAR framework, which is one of the first such set of results in literature, to our knowledge.

## 3. Extension to Vector Autoregressive Models

### 3.1 Proposed Model

Consider the data matrix $X_{i}=\left(x_{i1},\ldots ,x_{iT}\right)$, where $x_{it}$ represents the (D×1) temporally dependent multivariate measurement for the i-th subject at the t-th time point (i=1,…,n,t=1,…,T). Note that our model can easily accommodate subject-specific scan lengths $\left(T_{i}\right)$; however we will assume $T_{i}=T$ from hereon in, to ease the exposition. Throughout, we will also assume a fixed dimension (D), and a pre-specified number of time scans (T), which is consistent with the routinely used fixed dimensional assumptions in the literature on non-parametric modeling of location-scale mixtures. Consider the VAR model:

(3) $$x_{it}=\sum_{k=1}^{min\{t-1,K\}}A_{ik}x_{i,t-1}+\epsilon_{it},\;\epsilon_{it}∼N\left(0,\Sigma_{i}\right),\;i=1,\ldots ,n,t=2,\ldots ,T,$$

where $A_{ik}$ denotes the D×D matrix of autocovariance parameters for subject i at lag k $(k=1,\ldots ,K),\Sigma_{i}\in S_{D\times D}$ denotes the time-invariant residual covariance for subject i, and the lag order (K) is pre-specified as per standard practice in the VAR model literature (Ghosh et al., 2018). Model (3) implies that the mean of $x_{t}$ depends on $x_{t-1},\ldots ,x_{1}$ when t≤K and on $x_{t-1},\ldots ,x_{t-K}$ for t>K, with $x_{i1}∼N\left(0,\Sigma_{i}\right)$ as per convention. As is common in practice, the intercept term is fixed to be zero and not included in (3).

In order to understand the properties of (3), it is imperative to note that the likelihood for the i-th sample can be written as a product of conditional densities as

(4) $$L\left(X_{i}|\Theta_{i},\Sigma_{i}\right)=\prod_{t=2}^{T}\phi_{\Sigma_{i}}\left(x_{it}-\sum_{k=1}^{min\{t-1,K\}}A_{ik}x_{i,t-k}\right)\times \phi_{\Sigma_{i}}\left(x_{i1}\right),\;i=1,\ldots ,n,$$

where $\Theta_{i}$ denotes the collection of autocovariance matrices for sample i across lags. For example, the likelihood for the ith sample under a VAR(2) model may be written as $\prod_{t=3}^{T}\phi_{\Sigma_{i}}\left(x_{it}-\sum_{k=1}^{2}A_{ik}x_{i,t-k}\right)\times \phi_{\Sigma_{i}}\left(x_{i2}-A_{i1}x_{i,1}\right)\times \phi_{\Sigma_{i}}\left(x_{i1}\right)$. The above likelihood in (4) will be used throughout in our treatment of VAR models. We note that (4) is a different way of representing the likelihood compared to the linear regression framework that is often used in single subject VAR models (Ghosh et al., 2018).

Our goal involves multi-subject VAR analysis by proposing suitable priors on $\left(\Theta_{i},\Sigma_{i}\right)$ in (3) to leverage common patterns of information across samples in an unsupervised and flexible manner. A natural framework for pooling information across subjects is via clustering, which also inherently results in model parsimony that is particularly important in our settings where the number of parameters grow with n. Such a clustering approach should enable straightforward posterior computation and result in theoretical guarantees. To this end, we extend the PDPM methodology to the case of multivariate time-series data that imposes independent DP mixture priors separately on Θ and Σ to induce multiscale clustering. Depending on the manner of the DP prior specification on the autocovariance elements, one can obtain different variants of the proposed method that allow for varying degrees of model parsimony and varying levels of information sharing within samples, via different patterns of autocovariance clusters. Such a multi-scale clustering approach is particularly relevant in the context of VAR models where the dimension of the autocovariance matrix increases quadratically with the outcome dimension D, making it imperative to avoid the assumption of replicated samples that is embedded in typical mixture modeling approaches. The resulting PDPM approach leads to a more fitting characterization of heterogeneity and greater accuracy, as illustrated via extensive numerical studies involving VAR models in the sequel. In addition, appropriate base measures in the DP can be chosen to encourage shrinkage in the autocovariance elements that facilitate feature selection, as well as to induce low rank decomposition for the residual covariance resulting in additional model parsimony.

#### Product of DP mixtures for VAR models:

In the following specifications, we will omit subscript i where appropriate, for notational convenience and as per convention (Wu and Ghosal, 2008; Canale and De Blasi, 2017). We propose the following PDPM prior

(5) $$\Theta =\left\{\mathrm{vec}\left(A_{1}\right),\ldots ,\mathrm{vec}\left(A_{K}\right)\right\}∼P_{\Theta },\;P_{\Theta }∼DP\left(\alpha_{1}P_{1}^{*}\right),\Sigma ∼P_{𝒮},\;P_{𝒮}∼DP\left(\alpha_{2}P_{2}^{*}\right),$$

where $\alpha_{1},\alpha_{2}$, represent precision parameters in the Dirichlet process, the base measure $P_{1}^{*}$ belongs to the space of probability measures ${𝒫}_{1}$ on ${𝒟}_{1}=\underset{K}{\underbrace{{ℜ}^{D^{2}\times 1}\times \ldots \times {ℜ}^{D^{2}\times 1}}}$, and the base measure $P_{2}^{*}$ belongs to the space of probability measures ${𝒫}_{2}$ on ${𝒟}_{2}=S_{D\times D}$. Model (5) specifies unknown distributions $P_{\Theta }$ and $P_{𝒮}$ on model parameters, that are modeled under independent DP priors. The resulting product of DP priors in (5) is defined on the space of densities 𝒫 with domain ${𝒟}_{1}\times {𝒟}_{2}$ and may be expressed as $\Pi^{*}(\Theta ,\Sigma )=P_{𝒮}(\Sigma )\times P_{\Theta }(\Theta )$. This prior specification translates to a *product* of DP *mixture of VAR (PDPM-VAR)* models that induces a prior Π on the space of probability densities ℱ for the data matrix X as follows:

(6) $$f_{P}(X)=\int \;\int \prod_{t=1}^{T}\phi_{\text{Σ}}\left(x_{t}-\sum_{k=1}^{\text{min}\{t-1,K\}}A_{k}x_{t-k}\right)dP_{\text{Θ}}(\text{Θ})dP_{𝒮}(\text{Σ})\;=\sum_{h_{1}=1}^{\infty }\sum_{h_{\sigma }=1}^{\infty }\pi_{h_{1}}\pi_{\sigma ,h_{\sigma }}\prod_{t=1}^{T}\phi_{{\text{Σ}}_{h_{\sigma }}}\left(x_{t}-\sum_{k=1}^{\text{min}\{t-1,K\}}A_{k,h_{1}}x_{t-k}\right),$$

where $\pi_{h_{1}}=\nu_{h_{1}}\prod_{l_{1}<h_{1}}\left(1-\nu_{l_{1}}\right),\nu_{h_{1}}∼Be\left(1,\alpha_{1}\right),\pi_{\sigma ,h_{\sigma }}=\nu_{\sigma ,h_{\sigma }}\prod_{l_{2}<h_{\sigma }}\left(1-\nu_{\sigma ,l_{2}}\right),\nu_{\sigma ,h_{\sigma }}∼\mathrm{Be}\left(1,\alpha_{2}\right)$, and further $\Sigma_{h_{\sigma }}∼P_{2}^{*},\left(\mathrm{vec}\left(A_{1,h_{1}}\right),\ldots ,\mathrm{vec}\left(A_{K,h_{1}}\right)\right)∼P_{1}^{*}$. We consider a broad class of base measures to study theoretical properties (Section 3.2), but for implementation we focus on specific choices for $\left(P_{1}^{*},P_{2}^{*}\right)$ that facilitate posterior computations (Section 4).

While (5) provides a greater degree of flexibility in terms of accommodating heterogeneity compared to existing DP mixture approaches, there is further scope for generalizing this approach to accommodate additional heterogeneity in lag-specific and row-specific relationships. Such generalizations become particularly important when clusters of samples tend to share common autocovariance elements for some but not all lags or have identical elements for only a subset of rows/nodes in the autocovariance matrices in practical applications. For example, the latter scenario arises when the effective clustering for the autocovariance elements is confined to a subset of rows in the matrix A, with the remaining rows being irrelevant with respect to clustering. Such aspects are routinely encountered in heterogeneous and high-dimensional clustering problems (Agrawal et al., 2005), such as our VAR settings of interest where the number of autocovariance parameters increase quadratically with the outcome dimension (D). We now generalize the PDPM-VAR method below to account for such heterogeneous settings.

#### Generalization across autocovariance rows:

It is possible to generalize the PDPM-VAR model in (5) in a manner that relaxes the restriction to have fully identical autocovariance matrices for all samples within a given autocovariance cluster. In particular, consider an approach that specifies independent priors on the VAR model parameters corresponding to each row of the autocovariance matrices, which results in row-specific clustering patterns. In particular, denote $A_{k,d^{\prime }•}$ as the $d^{\prime }$-th row of $A_{k}$ and consider the following specification

(7) $$\mathrm{vec}\left\{A_{1,d^{\prime }•}^{\prime },\ldots ,A_{K,d^{\prime }•}^{\prime }\right\}\overset{\mathrm{indep}}{∼}P_{\Theta_{d^{\prime }}},\;P_{\Theta_{d^{\prime }}}∼DP\left(\alpha_{d^{\prime }}^{*}P_{1d^{\prime }}^{**}\right),\;\Sigma ∼P_{𝒮},\;P_{𝒮}∼DP\left(\alpha_{2}P_{2}^{*}\right),d^{\prime }=1,\ldots ,D,$$

where $A^{\prime }$ denotes the transpose of A, and the row-specific priors $P_{\Theta_{d^{\prime }}}\left(vec\left\{A_{1,d^{\prime }•}^{\prime },\ldots ,A_{K,d^{\prime }•}^{\prime }\right\}\right)$ are specified independently for each row and jointly across lags. The product of DP prior in (7) is expressed as $\Pi^{*}(\Theta ,\Sigma )=P_{𝒮}(\Sigma )\times \prod_{d^{\prime }=1}^{D}P_{\Theta_{d^{\prime }}}\left(vec\left\{A_{1,d^{\prime }•}^{\prime },\ldots ,A_{K,d^{\prime }•}^{\prime }\right\}\right)$, and results in the row-generalized PDPM-VAR (rgPDPM-VAR) model that induces priors on ℱ via

(8) $$f_{P}(X)=\sum_{h_{1,1}=1}^{\infty }\ldots \sum_{h_{1,D}=1}^{\infty }\sum_{h_{\sigma }=1}^{\infty }\left(\pi_{\sigma ,h_{\sigma }}\prod_{d^{\prime }=1}^{D}\pi_{d^{\prime },h_{1d^{\prime }}}^{*}\right)\prod_{t=1}^{T}\phi_{\Sigma_{h_{\sigma }}}\left(x_{t}-\sum_{k=1}^{min\{t-1,K\}}A_{k,h_{11},\ldots ,h_{1D}}x_{t-k}\right),$$

where $A_{k,h_{11},\ldots ,h_{1D}}$ denotes the autocovariance matrix at lag k that assigns the $h_{1d^{\prime }}$-th mixture component to the $d^{\prime }$-th row with prior probability $\pi_{d^{\prime },h_{1d^{\prime }}}^{*}=\nu_{d^{\prime },h_{1d^{\prime }}}^{*}\prod_{l_{1d^{\prime }}<h_{1d^{\prime }}}\left(1-\nu_{l_{1d^{\prime }}}^{*}\right)$, where $\nu_{d^{\prime },h_{1d^{\prime }}}^{*}∼\mathrm{Be}\left(1,\alpha_{d^{\prime }}^{*}\right),\mathrm{vec}\left\{A_{1,d^{\prime }•,h_{1,d^{\prime }}}^{\prime },\ldots ,A_{K,d^{\prime }•,h_{1,d^{\prime }}}^{\prime }\right\}\overset{\mathrm{indep}}{∼}P_{1d^{\prime }}^{**}$ and $A_{k,d^{\prime }•,h_{1,d^{\prime }}}$ denotes the $d^{\prime }$-th row for the matrix $A_{k}$ that takes values from the $h_{1,d^{\prime }}$-th mixture component. Further, $\Sigma_{h_{\sigma }}∼P_{2}^{*}$ with prior probability $\pi_{\sigma ,h_{\sigma }}=\nu_{\sigma ,h_{\sigma }}\prod_{l_{2}<h_{\sigma }}\left(1-\nu_{\sigma ,l_{2}}\right)$ and $\nu_{\sigma ,h_{\sigma }}∼Be\left(1,\alpha_{2}\right)$.

In the scenario when multiple rows have identical clustering configurations, the rgPDPM-VAR model is able to identify clusters of samples that share identical autocovariance elements corresponding to a subset of nodes only, but exhibit variations corresponding to the remaining autocovariance rows. We note that for our motivating neuroimaging applications, this scenario translates to identical effective connectivity corresponding to a subset of brain regions within a autocovariance cluster, while the remaining brain regions are allowed to exhibit varying connectivity profiles within this cluster. By allowing row-specific clustering patterns in the autocovariance matrix, the rgPDPM-VAR approach results in a more complete characterization of heterogeneity compared to the PDPM-VAR. Additional generalizations are also possible; for example, one may extend specification (7) to impose row- and lag-specific priors. However, such extensions may result in a rapid rise in parameters that presents potential computational issues, and hence are not considered further.

#### Generalization across lags:

For the second extension, we specify independent DP priors for the autocovariance matrices at each lag, which results in lag-specific clustering as follows:

(9) $$\mathrm{vec}\left(A_{k}\right)\overset{\mathrm{indep}}{∼}P_{\Theta_{k}},\;P_{\Theta_{k}}∼DP\left(\alpha_{1k}P_{1k}^{*}\right),\;\Sigma ∼P_{𝒮},P_{𝒮}∼DP\left(\alpha_{2}P_{2}^{*}\right),\;k=1,\ldots ,K,$$

where $P_{\Theta_{k}}$ denotes the unknown density for $\mathrm{vec}\left(A_{k}\right)$ that is modeled under a DP prior with base measure $P_{1k}^{*}$ and precision parameter $\alpha_{1k}(k=1,\ldots ,K)$, and the prior on the residual covariance parameters is defined similarly to (5), but with the understanding that $\alpha_{2}$ and $P_{2}^{*}$ in the DP priors in (9) and (5) are allowed to be distinct. The resulting product of DP priors in (9) may be expressed as $\Pi^{*}(\Theta ,\Sigma )=P_{𝒮}(\Sigma )\times \prod_{k=1}^{K}P_{\Theta_{k}}\left(A_{k}\right)$. As under the PDPM-VAR, specification (9) induces a prior on the space of densities ℱ via

(10) $$f_{P}(X)=\sum_{h_{11},\ldots ,h_{1K}=1}^{\infty }\sum_{h_{\sigma }=1}^{\infty }\pi_{\sigma ,h_{\sigma }}\left(\prod_{k=1}^{K}\pi_{k,h_{1k}}\right)\prod_{t=1}^{T}\phi_{\Sigma_{h_{\sigma }}}\left(x_{t}-\sum_{k=1}^{min\{t-1,K\}}A_{k,h_{1k}}x_{t-k}\right),$$

where $\pi_{k,h_{1k}}=\nu_{k,h_{1k}}\prod_{l_{k,1k}<h_{k,1k}}\left(1-\nu_{k,l_{1k}}\right)\;(k=1,\ldots ,K),\pi_{\sigma ,h_{\sigma }}=\nu_{\sigma ,h_{\sigma }}\prod_{l_{2}<h_{\sigma }}\left(1-\nu_{\sigma ,l_{2}}\right)$ and $\nu_{k,h_{1k}}∼\mathrm{Be}\left(1,\alpha_{1k}\right),\nu_{\sigma ,h_{\sigma }}∼\mathrm{Be}\left(1,\alpha_{2}\right)$, and further $\mathrm{vec}\left(A_{k,h_{1k}}\right)∼P_{1k}^{*},\Sigma_{h_{\sigma }}∼P_{2}^{*}$ for k=1,…,K, using the stick-breaking construction in Sethuraman (1994). We denote the model under (3) and (9) as the lag-generalized product of DP mixture of VAR (lgPDPM-VAR) model and note that this model reduces to the PDPM-VAR for lag 1 models. This approach is expected to be less flexible compared to the rgPDPM-VAR method in general, but may exhibit some advantages when the clustering patterns are distinct across lags.

### 3.2 Theoretical Properties

#### Notations and Definitions:

In this section we will establish posterior consistency properties of the proposed product of DP mixture of VAR models. We will assume that the D×T data matrices $X_{1},\ldots ,X_{n}$, are i.i.d. under some true density $f_{0}\in ℱ$. We note that the theoretical derivations corresponding to VAR models involving multivariate time-series data are more involved than the independent multivariate outcome settings in Section 2 that has been the focus of existing non-parametric Bayesian density estimation literature. Moreover, our theoretical results assume fixed T (finite time set-up) with growing number of samples, which is in contrast to theoretical settings in parametric VAR analysis for single subjects that rely on growing T (Ghosh et al., 2018).

Throughout the article, we will assume the following reasonable regularity conditions on $f_{0}\left(x_{t}\;|\;X_{1:(t-1)}\right)$ that reflect counterparts of the assumptions (C1)-(C4) corresponding to multivariate density estimation. Here, $f_{0}\left(x_{t}\;|\;X_{1:(t-1)}\right)$ denotes the true conditional density of $x_{t}$ that depends on previous time scans up to a certain known lag (K).

*(A0)* The form of the true density satisfies $f_{0}(X)=\left\{\prod_{t=1}^{T}f_{0}\left(x_{t}\;|\;x_{t-1},\ldots ,x_{1}\right)\right\}=\left\{\prod_{t=1}^{T}f_{0}\left(x_{t}\;|\;X_{1:(t-1)}\right)\right\}$, for all $X\in {ℜ}^{D\times T}$.

*(A1)* $0<f_{0}(X)<M$ for some constant M and for all $X\in {ℜ}^{D\times T}$.

*(A2)* $\left|\int \;f_{0}\left(x_{t}\;|\;X_{1:(t-1)}\right)log\left(f_{0}\left(x_{t}\;|\;X_{1:(t-1)}\right)\right)dx_{t}\right|<\infty$, point-wise for $X_{1:(t-1)}$ for all t.

*(A3)* For all t and some $\delta >0,\int f_{0}\left(x_{t}\;|\;X_{1:(t-1)}\right)log\left(\frac{f_{0}\left(x_{t}|X_{1:(t-1)}\right)}{\phi_{\delta }^{*}\left(x_{t}|X_{1:(t-1)}\right)}\right)dx_{t}\;<\;\infty$, where $\phi_{\delta }^{*}\left(x_{t}\;|\;X_{1:(t-1)}\right)={inf}_{\left\|r-x_{t}\right\|<\delta }f_{0}\left(r|X_{1:(t-1)}\right)$, point-wise for $X_{1:(t-1)}$.

*(A4)* For all t and some $\eta >0,\int {\left\|x_{t}\right\|}^{2(1+\eta )}f_{0}\left(x_{t}\;|\;X_{1:(t-1)}\right)dx_{t}<\infty$, point-wise for $X_{1:(t-1)}$.

Condition *(A0)* expresses the true density as a product of conditional densities, subject to a known K, where the true conditional density only depends on $x_{t-1},x_{t-2},\ldots ,x_{t-K}$, when t>K and depends on $x_{t-1},x_{t-2},\ldots ,x_{1}$ for t≤K. Condition *(A1)* assumes that the true density is bounded. Further, assumptions (A1)-(A4) are reminiscent of conditions used for conditional density estimation in Pati et al. (2013) who focused on dependent stick-breaking processes. In the special case when the true density corresponds to a VAR structure, *(A0)*-*(A4)* would imply (among other things) that the true VAR parameters are well-behaved and satisfy stability conditions so that the true density does not blow up to ∞ or attenuate to zero.

The following Theorem formally states the result on positive prior support under the above assumptions. The proof is provided in the Appendix and uses key results in Wu and Ghosal (2008) for multivariate density estimation under DP mixtures.

**Theorem 3:** *Suppose assumptions* (*A0*) – (*A4*) *are satisfied. Then the product of DP mixture priors* Π *specified in* (5), (7), *and* (9) *satisfies the Kullback-Leibler property, i.e.* $\Pi \left(f\in ℱ\;:\;\int \;log\left(f_{0}/f\right)f_{0}\leq \eta^{*}\right)\geq 0$, *for any* $\eta^{*}>0$.

The next goal is to establish strong consistency for the proposed approach. We will again leverage the sufficiency conditions in Theorem 2 that rely on careful sieve constructions. In practice, it may not be straightforward to construct such sieves for the matrix-variate density estimation case, since the metric entropy depends on a number of terms including the sample size n, dimension D, as well as T (see Theorem 4). A major contribution of our work is to construct appropriate sieves using the stick-breaking representation and inspired by the ideas implemented in Shen et al. (2013), which satisfy the conditions in Theorem 2.

#### Sieve Constructions:

The sieves are constructed so as to allow the norm of the elements in the autocovariance matrices, as well as the condition number of the residual covariance matrices, to increase with sample size at an appropriate rate that satisfies the conditions in Theorem 2. We note that the condition number of a matrix frequently appears in the random matrix literature (Edelman, 1988) and is defined as the ratio of the largest to the smallest eigen values, i.e. $\lambda_{1}(\Sigma )/\lambda_{D}(\Sigma )=\lambda_{1}\left(\Sigma^{-1}\right)/\lambda_{D}\left(\Sigma^{-1}\right)$. For our purposes, we construct the following sieves corresponding to the PDPM-VAR model in (3) and (5) as:

(11) $${ℱ}_{n}=\{f_{p}\;:\;P=\underset{h_{1}\geq 1}{\sum }\underset{h_{\sigma }\geq 1}{\sum }\pi_{h_{1}}\pi_{\sigma ,h_{\sigma }}\delta \Theta_{h_{1}},{\text{Σ}}_{h_{\sigma }}\;:\;\underset{h_{1}>H_{n}}{\sum }\pi_{h_{1}}<\epsilon_{1}\underset{h_{\sigma }>H_{n}}{\sum }\pi_{\sigma ,h_{\sigma }}<\epsilon_{2},\text{and for}\;h_{\sigma }\leq H_{n},{\underline{\sigma }}_{n}^{2}\leq \lambda_{D,\Sigma_{h_{\sigma }}}\leq \lambda_{1,h_{\sigma }}\leq {\underline{\sigma }}_{n}^{2}{\left(1+\epsilon /\sqrt{D}\right)}^{M_{n}},\;1<\frac{\lambda_{1,h_{\sigma }}}{\lambda_{D,h_{\sigma }}}\leq n^{H_{n}}\},\;{ℱ}_{n,\mathrm{jl}}=\left\{f_{p}\in {ℱ}_{n}\;:\;\text{for}\;h_{1},h_{\sigma }\leq H_{n},\underline{a_{h_{1,j}}}\leq \left\|\mathrm{vec}\left(A_{k,h_{1}}\right)\right\|\leq {\bar{a}}_{h_{1,j}}\forall k,\underline{u_{h_{\sigma },l}}\leq \frac{\lambda_{1,h_{\sigma }}}{\lambda_{D,h_{\sigma }}}\leq u_{h_{\sigma ,}l}\right\},$$

where $\delta_{\theta }$ denotes the probability measure degenerate at $\theta ,\lambda_{d^{\prime },\Sigma_{h_{\sigma }}}$ is a shorthand for $\lambda_{d^{\prime }}\left(\Sigma_{h_{\sigma }}\right)$, i.e. the eigen values corresponding to $\Sigma_{h_{\sigma }},j,l$ are integers that are $\leq H_{n}$ for a given n, the sequences $\left\{H_{n}\right\},\left\{M_{n}\right\},\left\{{\underline{\sigma }}_{n}\right\}\left\{\underline{a_{h_{1},j}}\right\},\left\{{\overset{‾}{a}}_{h_{1},j}\right\},\left\{\underline{u_{h_{\sigma },j}}\right\},\left\{u_{h_{\sigma },j}\right\}$ grow to ∞ with n and are chosen appropriately such that ${ℱ}_{n}\subset \cup_{j,l}{ℱ}_{n,jl}$, and further, ${ℱ}_{n}↑ℱ$ as n→∞. Moreover, the sieves corresponding to rgPDPM-VAR in (3) and (7) are constructed as:

(12) $${ℱ}_{n}=\{f_{p}\;:\;P=\sum_{h_{1,1}=1}^{\infty }\cdot \cdot \sum_{h_{1,D}=1}^{\infty }\sum_{h_{\sigma }=1}^{\infty }\left(\pi_{\sigma ,h_{\sigma }}\prod_{d^{\prime }=1}^{D}\pi_{d^{\prime },h_{1d^{\prime }}}^{*}\right)\delta \Theta_{h_{1d^{\prime }},\Sigma_{h_{\sigma }}}\;:\;\sum_{h_{1d^{\prime }}>H_{n}}\pi_{d^{\prime },h_{1d^{\prime }}}^{*}<\epsilon_{1},\;\forall d^{\prime }\leq D,\;\sum_{h_{\sigma }>H_{n}}\pi_{\sigma ,h_{\sigma }}<\epsilon_{2},\;\text{and for}\;h_{\sigma }\leq H_{n},{\underline{\sigma }}_{n}^{2}\leq \lambda_{D,h_{\sigma }}\leq \lambda_{1,h_{\sigma }}\leq {\underline{\sigma }}_{n}^{2}{\left(1+\epsilon /\sqrt{D}\right)}^{M_{n}},\;1<\frac{\lambda_{1,h_{\sigma }}}{\lambda_{D,h_{\sigma }}}\leq n^{H_{n}}\},\;{ℱ}_{n,\mathrm{jl}}=\{f_{p}\in {ℱ}_{n}\;:\;{\underline{a}}_{h_{1d^{\prime }},j}\leq \left\|\mathrm{vec}\left(A_{k,d^{\prime }}•,h_{1,d^{\prime }}\right)\right\|\leq {\bar{a}}_{h_{1d^{\prime }},j}\;\text{for}\;h_{11},\ldots ,h_{1D}\leq H_{n},\;\text{and}\;d^{\prime }=1,\ldots ,D,\;\text{and}\;{\underline{u}}_{h_{\sigma },l}\leq \frac{\lambda_{1,h_{\sigma }}}{\lambda_{D,h_{\sigma }}}\leq u_{h_{\sigma },l},\;\text{for}\;h_{\sigma }\leq H_{n}\},$$

and the sieves for the lgPDPM-VAR model in (3) and (9) are constructed similarly as:

(13) $${ℱ}_{n}=\{f_{p}\;:\;P=\sum_{h_{11}=1}^{\infty }\cdots \sum_{h_{1K}=1}^{\infty }\sum_{h_{\sigma }=1}^{\infty }\pi_{\sigma ,h_{\sigma }}\left(\prod_{k=1}^{K}\pi_{k,h_{1k}}\right)\delta \Theta_{h_{1k},\Sigma_{h_{\sigma }}}\;:\;\sum_{h_{1,1k}>H_{n}}\pi_{k,h_{1k}}<\epsilon_{1},\;\forall k=1,\ldots ,K,\;\sum_{h_{\sigma }>H_{n}}\pi_{\sigma ,h_{\sigma }}<\epsilon_{2},\;\text{and for}\;h_{\sigma }\leq H_{n},{\underline{\sigma }}_{n}^{2}\leq \lambda_{D}\left(\Sigma_{h_{\sigma }}\right)\leq \lambda_{1}\left(\Sigma_{h_{\sigma }}\right)\leq {\underline{\sigma }}_{n}^{2}{\left(1+\epsilon /\sqrt{D}\right)}^{M_{n}},1<\frac{\lambda_{1,h_{\sigma }}}{\lambda_{D,h_{\sigma }}}\leq n^{H_{n}}\},\;{ℱ}_{n,jl}=\left\{f_{p}\in {ℱ}_{n}:{\underline{a}}_{h_{1k},j}\leq \left\|\mathrm{vec}\left(A_{k,h_{1k}}\right)\right\|\leq {\bar{a}}_{h_{1k},j}\;\text{for all}\;h_{1k}\leq H_{n},{\underline{u}}_{h_{\sigma },l}\leq \frac{\lambda_{1,h_{\sigma }}}{\lambda_{D,h_{\sigma }}}\leq u_{h_{\sigma },l}\;,h_{\sigma }\leq H_{n}\right\},$$

where ${ℱ}_{n}\subset \cup_{j,l}{ℱ}_{n,jl}$ and ${ℱ}_{n}\;↑\;ℱ$ as n→∞, and it is understood that the sequences $\left\{H_{n}\right\},\left\{M_{n}\right\},\left\{{\underline{\sigma }}_{n}\right\},\left\{\underline{a_{h_{1},j}}\right\},\left\{{\overset{‾}{a}}_{h_{1},j}\right\},\left\{u_{h_{\sigma },l}\right\},\left\{u_{h_{\sigma },l}\right\}$ are chosen appropriately and can be specific to sieves corresponding to PDPM-VAR, lgPDPM-VAR or rgPDPM-VAR. The following results establish entropy bounds that are vital to establishing strong consistency.

**Theorem 4:** *The entropy bound for sieves* (11) *satisfies* $N\left(\epsilon ,{ℱ}_{n,jl},\|\cdot {\|}_{1}\right)≲{\left(\frac{M^{D}}{\epsilon^{-C_{1}}}\right)}^{H_{n}}\times \prod_{h_{\sigma }\leq H_{n}}\;{\left\{\frac{2Du_{h\sigma ,l}}{\epsilon^{2}}\right\}}^{D(D-1)/2}\times \prod_{h_{1}\leq H_{n}}{\left\{{\left(\frac{C_{h_{1,j},h_{\sigma ,l}}^{*}{\overset{‾}{a}}_{h_{1},j}}{{\underline{\sigma }}_{n}\epsilon }+1\right)}^{D^{2}}-{\left(\frac{C_{h_{1,j},h_{\sigma ,l}}^{*}{\underline{a}}_{h_{1},j}}{{\underline{\sigma }}_{n}\epsilon }-1\right)}^{D^{2}}\right\}}^{K}$, *where constants* $C_{1}>0$ *and* $C_{h_{1,j},h_{\sigma ,l}}^{*}>0$ *depend on* (D,T,K).

**Corollary 1:** *The entropy for sieves in* (12) *and* (13) *corresponding to rgPDPM-VAR and lgPDPM-VAR respectively, satisfy* $N\left(\epsilon ,{ℱ}_{n,jl},\|\cdot {\|}_{1}\right)≲{𝒦}^{*}{\left(\frac{M^{D}}{\epsilon^{-C_{1}}}\right)}^{H_{n}}\times \prod_{h_{\sigma }\leq H_{n}}{\left\{\frac{2Du_{h_{\sigma },l}}{\epsilon^{2}}\right\}}^{D(D-1)/2}$, *where* ${𝒦}^{*}$ *is understood to vary depending on the specific variant of the PDPM model used.*

Having established the entropy bounds, the next step is to propose sensible base measures that satisfy the tail conditions and summability constraints in Theorem 2. These base measures include some commonly used choices as discussed in the sequel.

*(B1)* The base measures corresponding to $P_{𝒮}$ in (5), (7) and (9) satisfy $P_{2}^{*}\left(\lambda_{1}\left(\Sigma_{h_{\sigma }}^{-1}\right)>x^{*}\right)≲exp\left(-c_{1}{\left(x^{*}\right)}^{c_{2}}\right),P_{2}^{*}\left(\lambda_{D}\left(\Sigma_{h_{\sigma }}^{-1}\right)<1/x^{*}\right)≲{\left(x^{*}\right)}^{-c_{3}},P_{2}^{*}\left(\frac{\lambda_{1}\left(\Sigma_{h_{\sigma }}^{-1}\right)}{\lambda_{D}\left(\Sigma_{h_{\sigma }}^{-1}\right)}>x^{*}\right)≲{\left(x^{*}\right)}^{-\kappa }$, for some positive constants $c_{1},c_{2},c_{3},\kappa$, and corresponding to the cluster $h_{\sigma }$.

*(B2)* The base measure corresponding to $P_{\Theta }$ specifies independence across lags, and satisfies the following tail conditions: (i) under PDPM-VAR, $P_{1}^{*}\left(\left\|vec\left(A_{k,h_{1}}\right)\right\|>x^{*}\right)≲{\left(x^{*}\right)}^{-2(r+1)}$ for cluster $h_{1}$; (ii) under lgPDPM-VAR, $P_{1k}^{*}\left(\left\|vec\left(A_{k,h_{1k}}\right)\right\|>x^{*}\right)≲{\left(x^{*}\right)}^{-2(r+1)}$ for cluster $h_{1k}$; and (iii) under rgPDPM-VAR, $P_{1d^{\prime }}^{*}\left(\left\|vec\left\{A_{1,d^{\prime }•,h_{1,d^{\prime }}}^{\prime },\ldots ,A_{K,d^{\prime }•,h_{1,d^{\prime }}}^{\prime }\right\}\right\|>x^{*}\right)≲{\left(x^{*}\right)}^{-2\left(r^{*}+1\right)}$ corresponding to cluster $h_{1d^{\prime }}$, for some constants $r,r^{*}>0$, and $d^{\prime }=1,\ldots ,D$.

The above conditions on the base measures are very reasonable and hold for commonly used distributions on autocovariance matrices (such as Gaussian and Laplace), as well as inverse-Wishart distribution corresponding to $P_{2}^{*}$. These tail conditions are also satisfied by certain low rank decompositions for the covariance, such as a factor model form ($\Sigma =\Lambda \Lambda^{T}+\Omega$ where the D×B matrix Λ contains B≪D factor loadings), which is particularly suitable for scaling up the approach to higher dimensions. Such low rank representations are routinely used for dimension reduction in the factor model literature (Ghosh and Dunson, 2009). Denote ${𝒜}_{d^{\prime },h_{1,d^{\prime }}}=\mathrm{vec}\left\{A_{1,d^{\prime }•,h_{1,d^{\prime }}}^{\prime },\ldots ,A_{K,d^{\prime }•,h_{1,d^{\prime }}}^{\prime }\right\}$ and let DE denote a double exponential prior. The following Lemmas formalize the above discussions on the base measures.

**Lemma 2:** *Condition (B2) holds when* $P_{1}^{*}\left(\mathrm{vec}\left(A_{k,h_{1}}\right)\right)$ *is specified as* $N_{D^{2}}\left(\mathrm{vec}\left(A_{k}\right);\mu ,\Lambda \right)$ *with* $\Lambda ∼IW\left(\Lambda_{0},\nu_{\lambda }\right)$ *corresponding to PDPM-VAR, and for a similar choice of* $P_{1k}^{*}\left(\mathrm{vec}\left(A_{k,h_{1k}}\right)\right)$ *under lgPDPM-VAR. It is also satisfied when* $P_{1d^{\prime }}^{**}\left({𝒜}_{d^{\prime },h_{1,d^{\prime }}}\right)=\prod_{k=1}^{K}N_{D}\left(A_{k,d^{\prime }•,h_{1,d^{\prime }}};\mu ,\Lambda_{d^{\prime }}\right),\Lambda_{d^{\prime }}∼\mathrm{IW}\left(\Lambda_{0d^{\prime }},\nu_{\lambda ,d^{\prime }}\right)$, *under rgPDPM-VAR. Further, (B2) also holds if the above base measures are changed to a product of independent* DE(λ) *priors with suitably large* λ.

**Lemma 3:** *Condition (B1) holds when for cluster* $h_{\sigma },P_{2}^{*}\left(\Sigma_{h_{\sigma }}\right)=IW\left(\Sigma_{h_{\sigma }};\Sigma_{0},\nu_{\sigma }\right)$, *as well as under the low rank representation* $\Sigma_{h_{\sigma }}=\Gamma_{h_{\sigma }}\Gamma_{h_{\sigma }}^{T}+\Omega_{h_{\sigma }}$ *where* $\Gamma_{h_{\sigma }}$ *is* D×B *and* $\Omega_{h_{\sigma }}=\mathrm{diag}\left(\sigma_{1,h_{\sigma }}^{2},\ldots ,\sigma_{D,h_{\sigma }}^{2}\right)$, *and* $P_{2}^{*}\left(\Sigma_{h_{\sigma }}\right)=\left\{\prod_{d^{\prime }=1}^{D}\prod_{m^{\prime }=1}^{B}N\left(\gamma_{d^{\prime }m^{\prime },h_{\sigma }};0,1\right)\right\}\left\{\prod_{j=1}^{D}Ga\left(\sigma_{j,h_{\sigma }}^{-2};a_{\sigma },b_{\sigma }\right)\right\}$.

The proof of Lemma 2 is provided in the Appendix, while that of Lemma 3 follows directly from Corollaries 1 and 2 in Canale and De Blasi (2017). We note that B<<D in Lemma 3 ensures a reduced rank structure on the residual covariance matrix.

One can now use the entropy bounds derived in Theorem 4 and Corollary 1 along with tail conditions in *(B1)*-*(B2)* to establish our strong consistency under a broad class of base measures, by applying Theorem 2. Our strong consistency result is stated below.

**Theorem 5:** *Suppose Theorem 3 holds, and (B1)-(B2) are satisfied. Then for suitably large constants* $r,r^{*},\kappa$, *the posterior distributions corresponding to the PDPM-VAR, lgPDPM-VAR and rgPDPM-VAR are strongly consistent at* $f_{0}$ *under suitable choice of sequences* $\left\{H_{n}\right\},\left\{M_{n}\right\},\left\{{\underline{\sigma }}_{n}\right\}\left\{\underline{a_{h_{1},j}}\right\},\left\{{\overset{‾}{a}}_{h_{1},j}\right\},\left\{\underline{u_{h_{\sigma },j}}\right\},\left\{u_{h_{\sigma },j}\right\}$ *in the sieves* (11), (12), *and* (13).

**Remark 3:** In mathematical terms, strong posterior consistency can be written as $\Pi (\{f\;:\;d\left(f,f_{0}\right)>\epsilon_{f}\}\;|\;X^{\left(1\right)},\ldots ,X^{\left(n\right)})\to 0$ as n→∞ in $F_{0}^{n}$ probability for any $\epsilon_{f}>0$.

**Remark 4:** While Theorem 5 is stated in terms of general class of base measures that satisfy *(B1)*-*(B2)*, we rely on commonly used base measures outlined in Lemmas 2-3 for implementing the proposed approach. We elaborate these choices in the next section.

## 4. Posterior Computation

We outline the posterior computation steps to fit all proposed VAR models that is the main focus of this work. Our approach alternates between sampling parameters related to the autocovariance matrices and the residual covariance matrix. For all models, we update the autocovariance parameters row-wise for one outcome at a time. For the PDPM-VAR, rgPDPM-VAR, and lgPDPM-VAR we use a hierarchical representation of Laplace base measures (Park and Casella, 2008). Under these base measures, these autocovariance elements follow independent DE(λ) distributions (Park and Casella, 2008). Explicit details are provided in Appendix C.

In order to scale up the implementation of the proposed method to high dimensional applications, we use a reduced rank factor model representation for the residual covariance matrix in our implementation, which provides a desired balance between computational scalability and theoretical flexibility. In particular, such a low rank structure on the residual covariance does not adversely impact the accuracy of parameter estimates compared to an unstructured covariance matrix, in our experience involving extensive numerical experiments with true unstructured residual covariances. Moreover, the results for estimation of the autocovariance terms are not particularly sensitive to the choice of rank. Further, it is considerably more flexible and results in greater accuracy compared to a diagonal residual covariance that is routinely used in VAR literature (Kook et al., 2021) but may be restrictive in practical applications. In particular, we specify $\Sigma_{i}=\Gamma_{i}\Gamma_{i}^{\prime }+\Psi_{i}$, where $\Gamma_{i}$ is a D×B factor loadings matrix with B(<<D) factors, and $\Psi_{i}$ is $diag\left\{\sigma_{i,1}^{2},\ldots ,\sigma_{i,D}^{2}\right\}$. To facilitate posterior computation, we use the following parameter expanded version of the model,

(14) $$x_{i,t}=\sum_{k=1}^{min\{t-1,K\}}A_{ik}x_{i,t-k}+\Gamma_{i}^{*}\eta_{i,t}^{*}+\epsilon_{i,t}^{*},\eta_{i,t}^{*}∼N\left(0,\Xi_{i}\right),\epsilon_{i,t}^{*}∼N\left(0,\Psi_{i}\right),$$

where $\Xi_{i}=diag\left\{\xi_{i,1},\ldots ,\xi_{i,B}\right\}$. Under the low rank representation, we impose DP mixture priors on $\left(\Gamma_{i}^{*},\Xi_{i},\Psi_{i}\right)$ leading to a mixture prior on $\Sigma_{i}$. This corresponds to the prior $\left.\Sigma_{i}∼\sum_{h_{\sigma }=1}^{\infty }\pi_{\sigma ,h_{\sigma }}\delta_{\left(\Gamma_{h\sigma }\right.}^{*},\Xi_{h_{\sigma }},\Psi_{h_{\sigma }}\right)$, where $\left(\Gamma_{h_{\sigma }}^{*},\Xi_{h_{\sigma }},\Psi_{h_{\sigma }}\right)∼P_{2}^{*}\equiv P_{\Gamma^{*}}\times P_{\Xi }\times P_{\Psi }$. Here $P_{\Gamma^{*}}$ is a product of independent standard normal distributions, $P_{\Xi }$ is a product of independent Gamma(1/2,1/2) distributions yielding a half-Cauchy prior on the diagonal elements of Γ and a Cauchy prior on the lower-off-diagonal elements as in Ghosh and Dunson (2009), and the inverse of the diagonal elements of Ψ have independent $\mathrm{Gamma}\left(\alpha_{\sigma },\beta_{\sigma }\right)$ priors.

### Computational Cost of the PDPM-VAR Approaches:

The proposed approaches are quite efficient as long as the number of nodes is not overly large. The computation is driven by the need to sample the rows of $A_{i}$ and the terms in the low rank representation for $\Sigma_{i}$ as per equation (14). We briefly discuss the computational costs below. In practice, the computational costs are quite manageable, as demonstrated by the HCP analysis in the sequel.

First, for the PDPM-VAR and rgPDPM-VAR, the rows of $A_{i}$ are updated one at a time, separately for each cluster. This corresponds to performing D draws, each from a DK-variate multivariate normal distribution, thus updating $A_{i}$ requires $D𝒪\left(D^{3}K^{3}\right)$ steps for the PDPM-VAR and rgPDPM-VAR. We note that this can also be parallelized over the outcomes as a possible direction for future work. The draw for the lgPDPM-VAR is more complicated. Although we still sample one row of $A_{i}$ at a time, we now must sample across all clusters, since different subjects can belong to different collections of clusters for a given row due to the lag-specific clustering structure. This means that we must draw from a $H_{\mathrm{all}}D$ dimensional multivariate normal distribution, where $H_{\mathrm{all}}$ is the total number of clusters across all lags. Therefore, this update requires $D\;𝒪\left(H_{\mathrm{all}}^{3}D^{3}\right)$ computational steps. We note that if one does not use the low rank decomposition in (14) but instead imposes a diagonal residual covariance structure, then the computational complexity remains similar.

In the scenario that an unstructured residual covariance structure is imposed but without a low rank decomposition, the computational complexity rapidly increases to $𝒪\left(HD^{6}K^{3}\right)$ due to the need to sample from a $KD^{2}$-variate normal distribution for each cluster. This may result in prohibitive computational costs for higher dimensions. Therefore, sampling from the low rank representation in model (14) is desirable, especially given that sampling the latent factors and their loading is computationally straightforward. In particular, each subject has a $B\times T_{i}$ dimensional matrix of latent factors to sample, where B is generally small and the time points are independent. Thus this simplifies to sampling a set of $T_{i}$ B-dimensional vectors, all of which have the same posterior covariance. Since $T_{i}$ is large relative to B, this means that the draw for the $\eta_{i}$ terms is driven by the cost to generate standard normal draws (each requiring 𝒪(1) operations), and subsequently multiply them with the lower triangular matrix from the Cholesky factorization of the posterior covariance of size B×B. Given that this must only be calculated once per cluster, the cost per subject is 𝒪(DBT). Thus the overall cost across all samples is $𝒪\left(HB^{3}\right)+𝒪(nDBT)$.

## 5. Simulation Studies

We compared the performance under the proposed approaches to a state-of-the-art single-subject VAR approach, as well as an ad-hoc clustering extension of the single subject VAR model that is able to borrow information across samples. We generated data for n=100,200, $D=100,T_{i}=250$, and different levels of sparsity within the autocovariance matrices were considered (75% and 90%). For each data generation setting we generate 25 simulation replicates, and for all settings the true VAR model involved K=2 lags. We consider four settings for generating the subject-level autocovariance matrices that differ with respect to the clustering structure. Settings 1-3 represent the PDPM-VAR, lgPDPM-VAR and rgPDPM-VAR scenarios respectively, while Setting 4 represents a more heterogeneous setting that is obtained via introducing additional random noise to the autocovariance elements generated under Setting 3. For Setting 1, we use 3 autocovariance clusters, for Setting 2 we use 3 clusters for lag 1 and 2 clusters for lag 2, and for Settings 3-4 we vary the true number of clusters randomly (between 2 – 5) across rows of the autocovariance matrix, and the elements of these matrices are generated randomly in order to ensure a stable time series. In Setting 3, subjects within a cluster share the exact same elements for the corresponding rows of the autocovariance matrix, whereas in Setting 4 the subject-level rows of the autocovariance matrix within a cluster are random deviations from a shared mean row. Each cluster’s residual covariance matrix was generated from an Inverse Wishart distribution with D degrees of freedom and diagonal scale matrix with elements equal to D/2. Subject level time courses were obtained by starting with random values for the multivariate observation at the first time point, and subsequently generating future observations from the assumed true VAR model. For a subject, an additional 5 time scans were generated after the initial $T_{i}$ observations to evaluate forecasting accuracy.

### 5.1 Approaches and Performance Metrics

We compare the proposed approaches to the single subject Bayesian VAR (SS-VAR) model developed in Ghosh et al. (2018), which separately models the time courses for each subject. We also consider a two-stage clustering extension of this method, where we first estimated subject specific autocovariance and residual covariances under the single VAR approach by Ghosh et al. (2018) and then applied the k–means clustering separately to the vectorized autocovariance and residual covariance matrix estimates. We choose the number of clusters to maximize the silhouette score (Rousseeuw, 1987) and we then allocate each sample to one of the k clusters that is based on both the autocovariance terms and the residual covariance estimates from the initial SS-VAR fit. We subsequently concatenate the time courses across all subjects within the same cluster in order to borrow information within cluster, and finally re-fit the SS-VAR model to this concatenated data separately for each cluster. Since the true clustering structure was assumed to be unknown when fitting the model, it was not possible to compare the performance with existing multi-subject VAR modeling methods that assume known groupings (Chiang et al., 2017; Kook et al., 2021).

We evaluate performance in terms of (1) autocovariance estimation accuracy, (2) clustering accuracy, (3) feature selection for identifying structural zeros in the autocovariance matrices, and (4) forecasting accuracy. Following Ghosh et al. (2018), we measure estimation accuracy using the relative L2 error of the estimates to the true estimates. Clustering accuracy is measured using the adjusted Rand index (Rand, 1971), which measures agreement between the assigned and true cluster labels, adjusted for chance agreement. Feature selection performance is evaluated via area under the receiver operating characteristic (RoC) curve and precision recall curve (PRC). To calculate both curves we considered a sequence of significance thresholds, and for each threshold, we examined the corresponding credible interval to infer the significance. The corresponding sequence of sensitivity versus 1-specificity values were plotted over varying thresholds in order to obtain the ROC curve, while the PRC was obtained by plotting the positive predictive value (1−FDR) against sensitivity (FDR denotes the false discovery rate). Finally, forecasting accuracy is measured via the relative L2 error of the predicted time courses for time scans $T_{i}+1,\ldots ,T_{i}+5$. The MCMC chains converged for all methods as assessed using Dickey-Fuller tests of stationarity, although the results are not displayed due to space constraints.

### 5.2 Simulation Results

**Figure 2:**
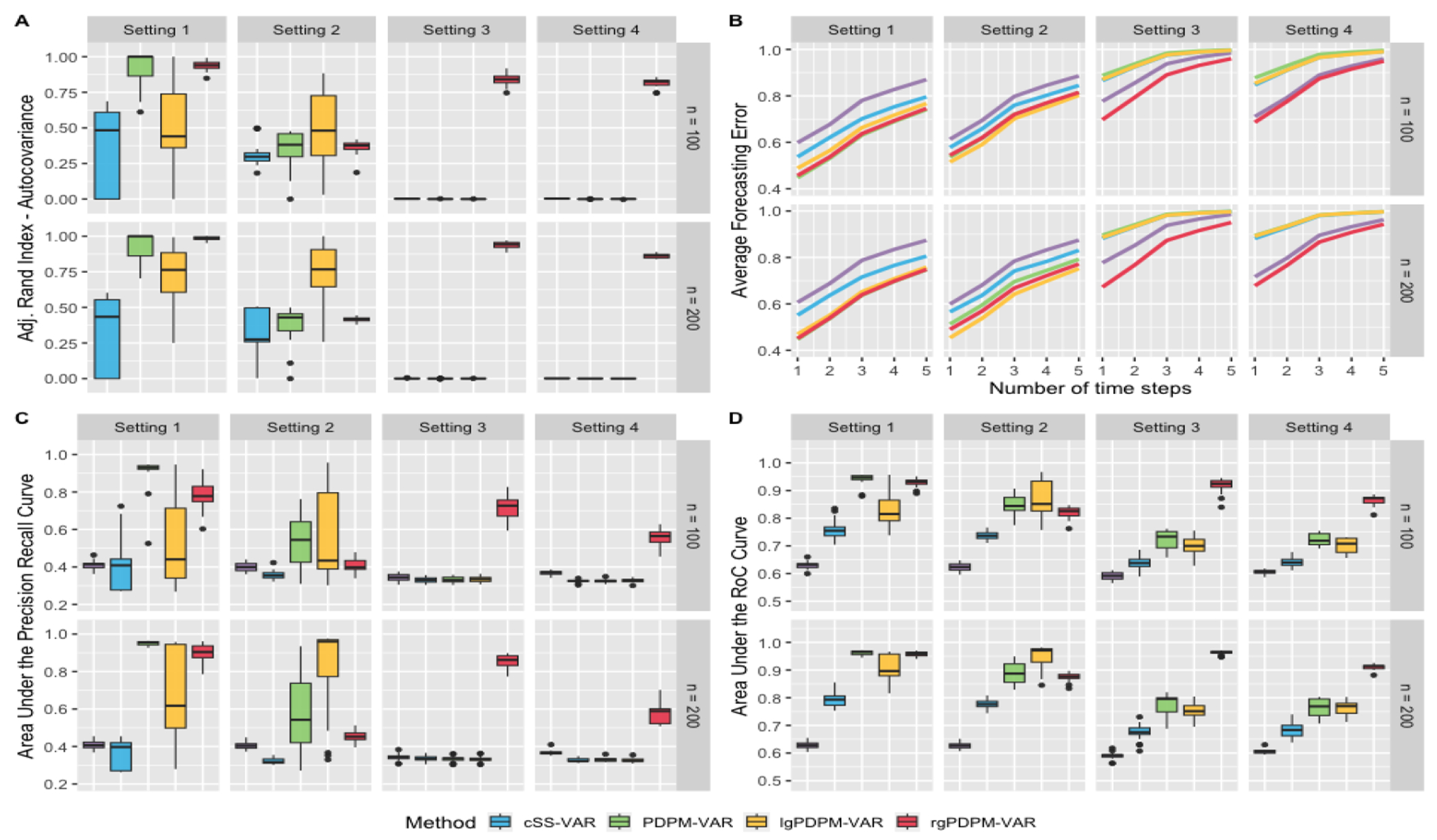
Simulation results for *D* = 100, *T* = 250 case with sparsity level 0.75. Panel A displays the adjusted Rand index for clustering the autocovariance. Panel B displays the forecasting error. Panels C and D display the area under the PR and RoC curves for identifying autocovariance non-zero elements.

**Figure 3:**
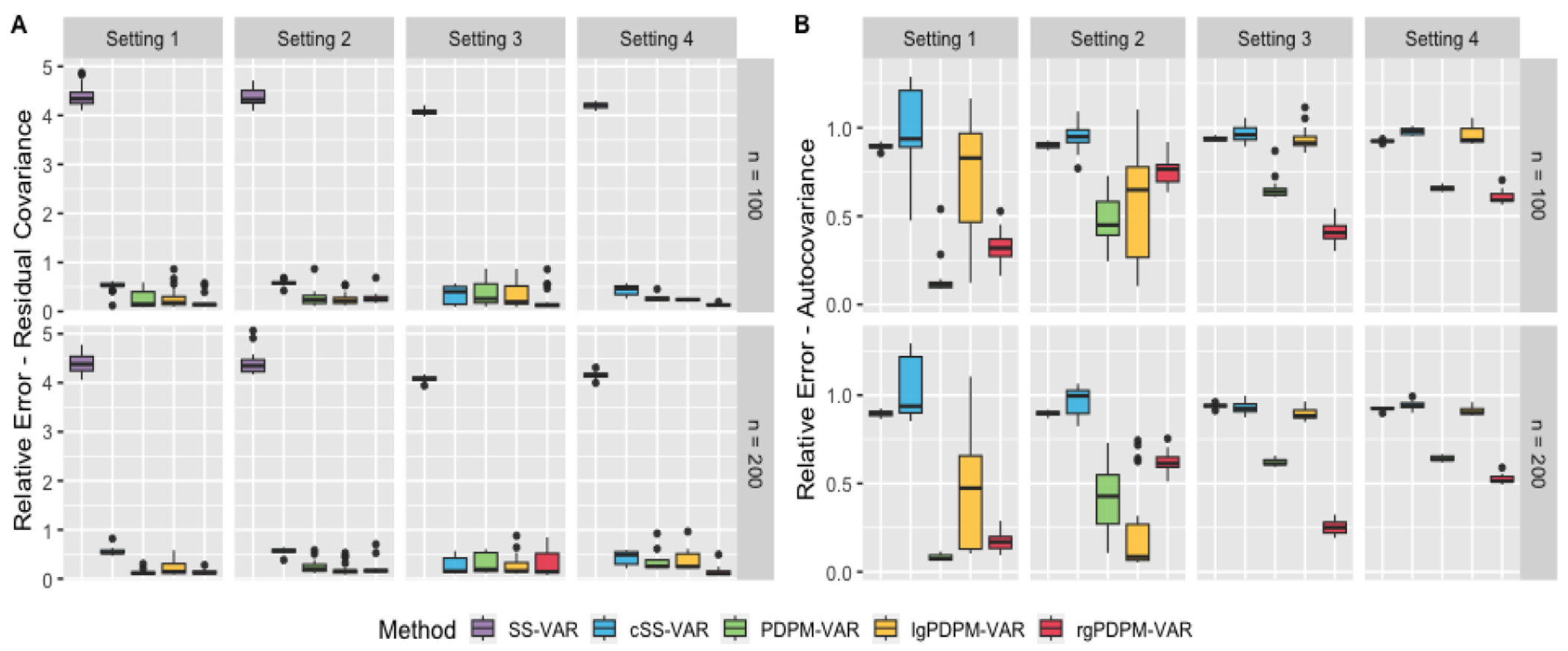
Relative L1 error for estimating the residual covariance (Panel A) and the subject-specific autocovariance matrices (Panel B) for *D* = 100, *T* = 250 case with sparsity level 0.75.

Simulation results are presented in Figures 2–3. Due to space constraints, we provide the simulation results for the most challenging case (D=100,T=250) at the 75% sparsity level here, but the results under the 90% sparsity case were quite similar. Several general patterns are clear from the results. First, the clustering performance for the autocovariance depends heavily on the true clustering structure (Figure 2, Panel A), with the PDPM-VAR, lgPDPM-VAR, and rgPDPM-VAR generally outperforming the other approaches when the data is generated from Settings 1-3 respectively. However, the rgPDPM-VAR often has close to optimal clustering performance when the PDPM-VAR is the true model and it also performs the best for the heterogeneous Setting 4, which reflects the generalizability of this variant. Critically, when there are differences in clustering across the different outcomes corresponding to more heterogeneous scenarios (Settings 3-4), only the rgPDPM-VAR is able to achieve a good clustering score. Finally, across all settings, the SS-VAR with clustering has the worst performance, demonstrating that the ad hoc two-stage analysis procedure is not able to accurately pool information across subjects.

The areas under the ROC and PR curves (Figure 2, Panels C and D) illustrate a consistently superior feature selection performance under the three proposed variants compared to the single subject VAR model with and without clustering. As expected, the PDPM-VAR, lgPDPM-VAR and rgPDPM-VAR approaches have higher area under the ROC and PR curves when the data is generated from Settings 1-3 respectively. In addition, the rgPDPM-VAR often has comparable area under the curve with PDPM-VAR for n=200 under Setting 1 and the best performance under the more heterogeneous Setting 4. These results imply the ability of rgPDPM-VAR to accurately identify the sparsity structure of the autocovariance with a low risk of false discoveries for data with unknown clustering.

When estimating the residual covariance matrices (Figure 3, Panel A), all three proposed approaches are able to heavily outperform the SS-VAR model. The performance under the three proposed approaches is generally comparable, with the rgPDPM-VAR outperforming the others in the more heterogeneous Settings 3 and 4. In addition, the SS-VAR approach with initial clustering has a higher relative error compared to the rgPDPM-VAR for the vast majority of cases, although it occassionally has a slightly improved performance in Setting 3. We conjecture that this is due to the assumed full rank structure for the residual covariance that is modeled via an inverse-Wishart distribution under the SS-VAR, which aligns with the true data generation scenario, in contrast to the assumed low-rank structure on the PDPM-VAR. Unfortunately, the SS-VAR approach with clustering has extremely poor performance in terms of autocovariance estimation (Figure 3 B), while the PDPM-VAR, lgPDPM-VAR, and rgPDPM-VAR approaches typically have the lowest errors when the data is generated from Settings 1-3 respectively. The rgPDPM-VAR method also has the best autocovariance estimation performance under the more heterogeneous Setting 4.

Figure 2 Panel B displays the forecasting error for each of the autocovariance clustering setups, averaged over the sparsity level and the number of time points per subject. With the exception of Setting 2 where lgPDPM-VAR performs best, the rgPDPM-VAR approach has the best or close to optimal forecasting performance for other settings. Moreover, in the more heterogeneous Settings 3-4, the SS-VAR method with initial clustering has better forecasting accuracy compared to the PDPM-VAR and lgPDPM-VAR approaches, although it can not outperform the rgPDPM-VAR method. The relative forecasting performance levels off for greater than three time steps for all approaches, as expected.

#### MCMC Diagnostics:

The MCMC was implemented via a Gibbs sampler and exhibited good mixing as measured by the effective sample size (Appendix Section 12). Moreover, we varied the dimension of the low rank representation of the covariance and found that the results were not particularly sensitive to this choice, however these results are excluded due to space constraints.

#### Synopsis of findings:

Overall, the rgPDPM-VAR provides a desirable balance between model parsimony and accurate estimation and inference for various degrees of heterogeneity across samples. The advantages under the rgPDPM-VAR are most pronounced under the heterogeneous Settings 3 and 4, and it often has close to optimal performance in Setting 1 for larger n. This illustrates the advantages of pooling information across subjects, while accommodating varying levels of heterogeneity at the level of the rows of the autocovariance matrix. While the SS-VAR approach with ad-hoc clustering is also able to pool information, it is highly sensitive to the clustering accuracy in the first step, and it can not capture clustering uncertainty, resulting in inferior performance.

## 6. Analysis of Human Connectome Project Data

### 6.1 Analysis Description

We use the rgPDPM-VAR approach to investigate effective connectivity differences between individuals with high and low fluid intelligence (FI) using a subset of resting-state fMRI data from the Human Connectome Project. Preprocessing details for these data can be found in (Smith et al., 2013). We adopt the 360-region Glasser atlas for parcellation as in Akiki and Abdallah (2019), where each node has a corresponding time course with T=1200. We centered and scaled the subject level time courses for each node before analysis, and verified that each node’s time course was stationary using Dickey-Fuller tests. We grouped the brain nodes into one of 6 well known functional brain networks (Akiki and Abdallah, 2019). These networks corresponded to the central executive (67 nodes), default mode (96 nodes), dorsal salience (23 nodes), somatomotor (55 nodes), ventral salience (49 nodes), and the visual network (70 nodes). We fit a separate VAR model with lag-1 on each of these networks, which corresponds to six separate VAR analyses. We selected the lag 1 model following previous literature on VAR models applied to fMRI data (Kook et al., 2021), and based on the lower temporal resolution of the fMRI data. We restrict our analysis to a subset of samples with the highest 10% and lowest 10% fluid intelligence scores, with n=306 samples. We note that the grouping information was only used for post-model fitting comparisons in effective connectivity across groups. We used 1500 burn-in and 3500 MCMC iterations.

To the best of our knowledge, our approach for analyzing fluid intelligence-related effective connectivity differences using heterogeneous multi-subject data is one of the first such attempts. Most existing approaches involve a single-subject VAR analysis, and subsequently these estimates are combined to estimate between-subject variations and examine group differences (Deshpande et al., 2009). There are a handful of approaches for estimating effective connectivity by pooling information across multiple subjects, however they assume known groups (Chiang et al., 2017) with limited heterogeneity within groups, and have similar limitations as outlined in the Introduction. Our analysis using the rgPDPM-VAR model is able to compute effective connectivity for multiple samples without any given group labels and can account for heterogeneity in an unsupervised manner. We compare the performance with a SS-VAR approach that analyses each sample separately, and subsequently performs permutation tests to assess significant differences (10,000 permutations). For both methods, false discovery rate control was applied to obtained significant elements.

In addition to investigating effective connectivity differences, we are interested in the clustering reliability and biological reproducibility of our findings. We report clustering reliability over two distinct MCMC runs, that are designed to evaluate the reliability of the clusters discovered by rgPDPM-VAR. As discussed in the introduction, for heterogeneous multivariate measurements, one can expect a subset of nodes/rows to drive the clustering whereas for other nodes the clustering patterns likely hold little information. We calculate the ARI for the node-level clustering across the two MCMC runs to investigate this aspect of clustering reliability. To assess biological reproducibility, we conduct our VAR analysis for two scans collected from each individual using different phase-encodings (LR1 and RL1), with the expectation that the parameter estimates should be similar corresponding to the two scans. We examine the correlation of the estimated autocovariance elements across the two runs (LR1 and RL1) under both the SS-VAR and the rgPDPM-VAR, with high correlation providing evidence that the findings are reproducible.

### 6.2 Results

**Figure 4:**
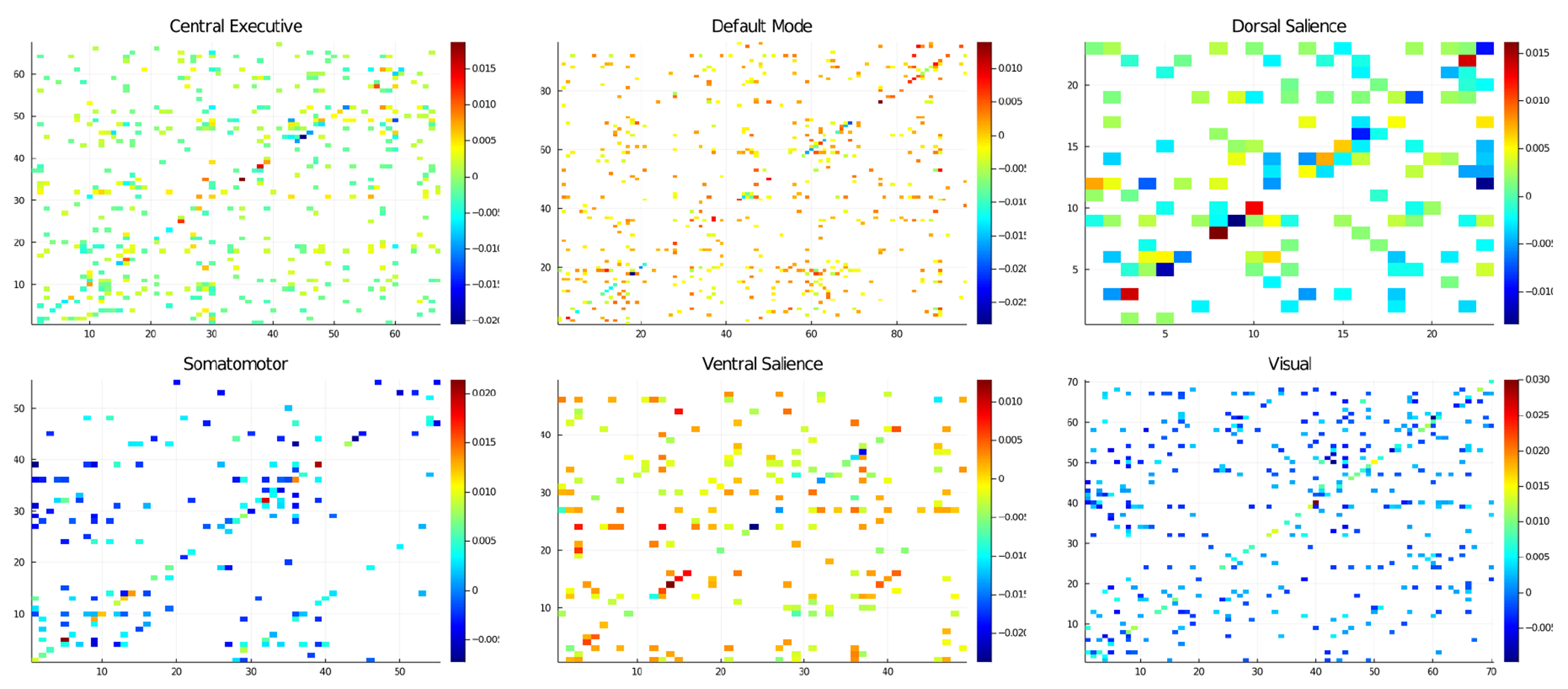
Elements of the autocovariance matrices exhibiting significant differences between the low and high FI groups. The color of the element represents the strength of the mean difference between groups (high FI – low FI), with white elements corresponding to non-significant elements.

Figure 4 displays heatmaps of the significant autocovariance differences between the low and high FI groups under the rgPDPM-VAR, after appropriate FDR control. Several patterns are clear from Figure 4. First, the rgPDPM-VAR is able to identify a large number of significant differences between the two groups after FDR control. Second, the rgPDPM-VAR finds a large number of strong differences along the diagonal. These correspond to AR(1) coefficients, and it seems sensible that if there are differences between groups at Lag 1 that they would be strongly related to each nodes’ own time course. Thirdly, the strongest differences were observed corresponding to the nodes in the Dorsal Salience network, as illustrated in Table 1. These nodes were identified by looking at columns of the autocovariance matrix with a large proportion of significant elements, which accounts for the varying sizes for the 6 networks. These findings are consistent with previous evidence, which have suggested the dorsal salience and attention networks to be highly related to fluid intelligence (Santarnecchi et al., 2017). We note that in contrast, only one significantly different effective connectivity difference between the high and low fluid intelligence groups was reported under the SS-VAR approach. Such results are clearly biologically implausible. Our overall findings point to the advantages of performing a multi-subject analysis accounting for heterogeneity, over a single subject analysis.

**Figure 5:**
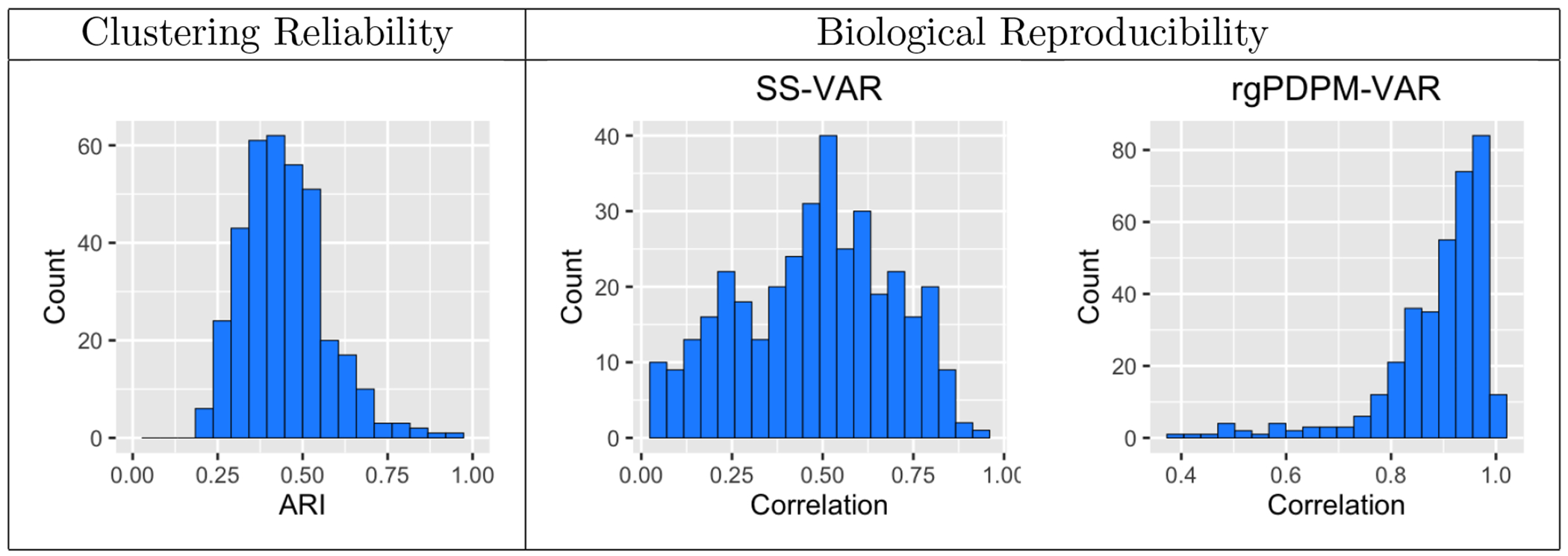
Adjusted Rand index across two runs of the analysis of the HCP LR1 data for assessing clustering reliability (left). Correlation between the rows of $A_{i}$ across two different HCP data sets (right). The first run of the analysis was on the LR1 phase-encoding data and the second on RL1.

To examine biological reproducibility, the right–hand side of Figure 5 displays histograms of the correlations of the rows of the autocovariance matrices across the two analyses corresponding to the LR1 and RL1 fMRI scans, under the SS-VAR and rgPDPM-VAR. The estimates under the rgPDPM-VAR exhibit a very high degree of correlation, almost entirely >0.8. On the other hand, the correlation for the majority of the elements is less than 0.5 under SS-VAR, with only 10 elements registering a correlation greater than 0.8, which implied considerably lower reproducibility overall compared to the multi-subject analysis. Moreover a non-negligible number of nodes had weak reproducibility with correlations less than 0.25 under SS-VAR. In addition, Figure 5 (left) displays a histogram of the ARI for clustering each node across two separate runs of MCMC on the LR1 data, which illustrates clustering reliability. In general, most nodes exhibited 9–10 clusters. As hypothesized in the Introduction, we see a pattern in which a subset of nodes exhibited very high clustering reliability across runs (>0.7), which supports our hypothesis that only some nodes contribute meaningfully towards clustering of samples. On the other hand, most of the nodes exhibited relatively moderate clustering reliability (ARI≈0.5), which indicates much higher clustering than chance, but not fully consistent clustering across all subjects corresponding to these nodes. We note that given the strong biological reproducibility results, the moderate or low clustering reliability for a subset of nodes in our analysis should be attributed to the fact that these nodes are irrelevant to clustering. This provides further justification for using the rgPDPM-VAR, which is designed to accommodate exactly this kind of clustering structure. Finally, the computation for the analysis is very efficient. On average, a single MCMC iteration required 1.8 (centeral executive), 3.8 (default mode), 0.3 (dorsal salience), 1.26 (somatomotor), 1.01 (ventral salience), and 1.9 (visual) seconds on an 8 core 2021 M1 Macbook Air.

## 7. Forecasting of Air Quality Data

While the fMRI study described above focuses on connectivity between brain regions, fMRI studies are not generally concerned with forecasting accuracy. To demonstrate the forecasting accuracy of our method, we next apply the proposed methods to an open source air quality data set from the EPA (https://www.epa.gov/outdoor-air-quality-data). The data consist of daily measurements from air quality monitors spread across the United States. For our purposes, we consider the air quality index time series for nitrogen dioxide $\left({NO}_{2}\right)$, ozone $\left(O_{3}\right)$, and carbon monoxide (CO). We used data from sensors having 100 days of consecutive data in 2000 from May 20th to August 27th. A simple kernel regression density plot for the data for each pollutant produced non-Gaussian curves, which motivates the use of non-parametric Bayesian analysis over parametric forecasting models. While our method does not require the data from each sensor to be overlapping, this restriction helps ensure that the forecasting results are due only to the method and not so some other data-dependent difference. Additionally, the relatively short time span helps reduce concerns about non-stationarity. For each time course, the time series were differenced (5 steps), demeaned, and outliers were replaced using the tsoutliers function in R (López-de Lacalle, 2024). The time courses were then checked for stationarity using a Dickey-Fuller test, and sensors for which the time courses were not stationary were removed from the data. After this procedure, we had 41 sensors with complete data.

We fit the rgPDPM-VAR model to this data using 1500 burn-in samples and 3500 MCMC samples. The concentration parameter was set to 5 to encourage more clusters to spawn. The resulting forecasting accuracy was measured in terms of the relative error and was 0.549 at step 1, 0.877 at step 2, and 0.929 at step 3. Thus the model shows considerable forecasting performance for 1 step forecasting, and naturally this performance degrades as the number of forecasting steps increases. As a comparison, we also used the SS-VAR approach of Ghosh et al. (2018) to analyse each sensor separately. The SS-VAR model illustrates decent performance, but is considerably worse that the rgPDPM-VAR, with a relative forecasting error of 0.598 at step 1, 0.897 at step 2, and 0.959 at step 3. This suggests that even for a small number of nodes, pooling information across sensors/subjects under the rgPDPM-VAR can still provide forecasting benefits. We note that other choices could have been made for the number of differencing steps. However, the overall relative performance between the two methods stays similar, with the proposed rgPDPM-VAR consistently performing better over different settings, as reported below.

## 8. Discussion

In this work, we developed a non-parametric Bayesian framework for i.i.d. multivariate data as well as multivariate time-series data, which provides a fundamentally novel way to borrow information across samples, via a class of novel product of DP mixture priors. The proposed approach employs multi-scale clustering to flexibly borrow information across heterogeneous samples that bypasses restrictive parametric assumptions and the requirement of replicated samples. The method is implemented via an efficient MCMC sampling scheme and computational complexity calculations are presented. The distinct numerical advantages over existing methods are illustrated via extensive numerical examples. The proposed class of methods are shown to have desirable posterior consistency properties that are derived based on novel sieve constructions and careful entropy calculations. Unlike single-subject parametric VAR modeling (Ghosh et al., 2018) that relies on an increasing T to establish posterior consistency, the proposed Bayesian non-parametric analysis focuses on posterior consistency corresponding to density estimation as n→∞. While it would be interesting to explore posterior consistency under our set-up as both T→∞,n→∞, there are potential theoretical challenges to be encountered. For example, increasing the number of timepoints T directly has an impact on the expression of the true density $f_{0}$, as well as on the form of $f_{P}(X)$. The latter has a direct impact on the sieve entropy (Theorem 4) that is intricately tied to the sufficient conditions for posterior consistency in Theorem 2. We plan to explore such aspects in future work.

While this work introduced several variants of the PDPM-VAR model, there are numerous potential extensions that lie within our class of models. In particular, future directions might investigate possible generalizations intended to induce sparsity in the parameter estimates. For example, a spike and slab prior could be used to model the autocovariance elements, with the slab component modeled using a DP mixtures. Additionally, the models could be generalized to accommodate even higher levels of heterogeneity, such as clustering individual autocovariance elements separately. However, such extensions may involve a massive computational burden. The proposed approaches, particularly the rgPDPM-VAR, seem to strike a desirable balance between computational complexity, clustering flexibility and model parsimony, with theoretical guarantees and appealing practical performance. Finally, we note that the proposed product of DP priors provides a viable improvement over traditional DP mixture models and it should have wide applicability to other types of settings that go beyond the VAR framework, which is of immediate interest in this article. We expect to pursue these directions in future research. For example, the VAR model structure could be relaxed to a dynamic linear model framework that can cater to non-stationary time-series, using a set of lower dimensional latent time courses to model the observed data (Sevestre and Trognon, 1996). Such an approach would potentially enrich the kinds of time courses that could be described by incorporating time-varying relationships.

## Figures and Tables

**Table 1:** Table of the 20 nodes with large proportion of significant effects on other nodes within their network. Note that the proportion is used instead of the raw count to account for the different network sizes.

Node	Network	Prop. FI Diff.
L_PF	Dorsal Salience	0.43
R_7Am	Dorsal Salience	0.35
L_IFSa	Dorsal Salience	0.35
L_PHT	Dorsal Salience	0.35
R_PHT	Dorsal Salience	0.30
R_PF	Dorsal Salience	0.30
L_6a	Dorsal Salience	0.30
L_PFt	Dorsal Salience	0.30
R_PGs	Central Executive	0.28
R_IFSa	Dorsal Salience	0.26
R_PFt	Dorsal Salience	0.26
L_PEF	Dorsal Salience	0.26
L_TE2p	Dorsal Salience	0.26
R_V3A	Visual	0.23
R_PSL	Ventral Salience	0.22
R_7PL	Dorsal Salience	0.22
R_6r	Dorsal Salience	0.22
L_7Am	Dorsal Salience	0.22
L_6r	Dorsal Salience	0.22
L_V3A	Visual	0.21

**Table 2:** Forecasting results under the rgPDPM-VAR and SS-VAR models for the air quality data with different levels of differencing. The values in the cells represent the relative L2 error between the model-based predictions and the true values, and the standard deviation is given in parenthesis.

Differencing Level	1 Step Forecasting		2 Step Forecasting		3 Step Forecasting	

	rgPDPM-VAR	SS-VAR	rgPDPM-VAR	SS-VAR	rgPDPM-VAR	SS-VAR
1	1.202 (0.043)	1.204 (0.120)	1.022 (0.089)	1.054 (0.146)	1.002 (0.037)	1.009 (0.053)
2	0.718 (0.064)	0.731 (0.118)	0.995 (0.131)	1.034 (0.140)	1.022 (0.021)	1.022 (0.041)
3	0.644 (0.143)	0.696 (0.254)	0.913 (0.376)	0.934 (0.411)	1.057 (0.129)	1.067 (0.178)
4	0.616 (0.133)	0.703 (0.226)	0.889 (0.212)	0.932 (0.256)	0.962 (0.227)	1.000 (0.498)
5	0.549 (0.118)	0.598 (0.167)	0.877 (0.247)	0.897 (0.237)	0.929 (0.127)	0.959 (0.150)

**Figure 6:**
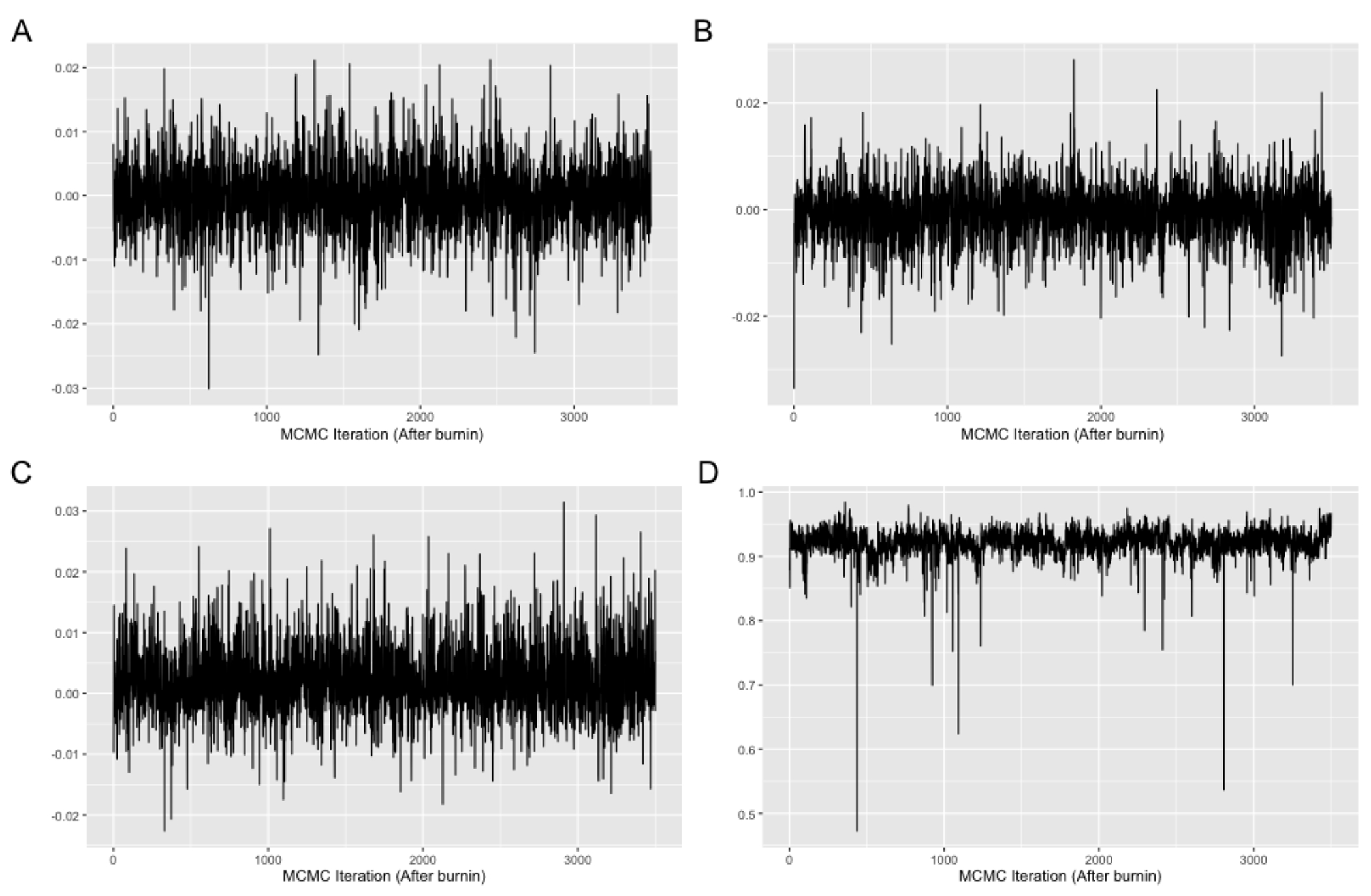
Trace plots for 4 selected elements of the subject-specific autocovariance matrices. (A) subject 34, node 49 lag 2 effect on node 25; (B) subject 38, node 23 lag 1 effect on node 86; (C) subject 31, node 70 lag 1 effect on node 77; (D) subject 38, node 23 lag 2 effect on node 45.

## Appendix

### Appendix A. Proofs of Results

**Proof of Lemma 1:** An outline for the proof of Lemma 1 is provided, which follows similar steps as the proof of Lemma 1 in Canale and De Blasi (2017). One may write $KL\left(f_{0},f_{P}\right)=KL\left(f_{0},f_{P_{\epsilon }}\right)+KL\left(f_{P_{\epsilon }},f_{P}\right)$ and then show that each of the terms in the right hand side can be made exceedingly small with positive probability, for some compactly supported $P_{\epsilon }$. The compact support for $P_{\epsilon }$ is taken as ${\left[-\mu^{*},\mu^{*}\right]}^{D}\times \left\{\Sigma \in 𝒮\;:\;{\underline{\sigma }}^{2}<\lambda_{l}(\Sigma )<{\overset{‾}{\sigma }}^{2},1\leq l\leq p\right\}$, for some constants $\mu^{*}>0$ and $0<\underline{\sigma }<\overset{‾}{\sigma }$, and eigenvalues denoted as $\lambda_{l},l=1,\ldots ,p$, the second term can also be shown to be infinitesimally small with positive probability.

**Proof of Theorem 1:** The proof relies on two parts, i.e calculating the entropy bounds and calculating the prior probability of the constructed sieves, and then using them to show the summability condition in Theorem 2 holds. We will illustrate the proof for the case of ${ℳ}_{\mu }=1$, and extensions to higher values of ${ℳ}_{\mu }$ are straightforward.

First, we will construct sieves of the following form -

(15)
$$F_{n}=\left\{f_{p}\;:\;P=\sum_{h_{1}\geq 1}\sum_{h_{\sigma }\geq 1}\pi h_{1}\pi_{\sigma },h_{\sigma }\delta \mu_{h_{1}},\sum_{h_{\sigma }}\;:\;\sum_{h_{1}>H_{n}}\pi_{h_{1}}<\epsilon ,\sum_{h_{\sigma }>H_{n}}\pi_{\sigma },h_{\sigma }<\epsilon ,\;\text{and for}h_{\sigma }\leq H_{n},{\underline{\sigma }}_{n}^{2}\leq \lambda_{D},\lambda_{1}\leq {\underline{\sigma }}_{n}^{2}{\left(1+\epsilon /\sqrt{D}\right)}^{M_{n}},\;1<\frac{\lambda_{1}(\sum_{h_{\sigma }})}{\lambda_{D}(\sum_{h_{\sigma }})}\leq n^{H_{n}}\right\},$$

(16)
$${ℱ}_{n,j,l}=\left\{f_{p}\in {ℱ}_{n}\;:\;\text{for}\;h_{1},h_{\sigma }\leq H_{n},n^{H_{n}^{2}}\left(j_{h_{1}}-1\right)={\underline{a}}_{h_{1},j}\leq \|\mu_{h_{1}}\|\leq {\bar{a}}_{h_{1},j}=n^{H_{n}^{2}}j_{h_{1}},k\in \{1,\ldots ,K\},n^{l_{h_{\sigma }}-1}<\frac{\lambda_{1}\left({\text{Σ}}_{h_{\sigma }}\right)}{\lambda_{D}\left({\text{Σ}}_{h_{\sigma }}\right)}\leq n^{l_{h_{\sigma }}}\right\},$$

where $M_{n}={\underline{\sigma }}^{-2c_{2}}=n$ and $H_{n}=\left\lfloor Cn\epsilon^{2}/log(n)\right\rfloor$ for some positive constant C, and clearly ${ℱ}_{n}\subset \cup_{j,l}{ℱ}_{n,j,l}$. Using similar techniques to those used in Lemma 6 in the Appendix, it is possible to show the tail condition (2*A*) holds in Theorem 2, i.e. $\Pi \left({ℱ}_{n}^{c}\right)\leq e^{-b^{*}n}$. Further, using similar steps as in the proof of Lemma 4 in the Appendix, the distance between the two densities $f_{P_{1}}$ and $f_{P_{2}}$ can be expressed as ${\left\|f_{P_{1}}-f_{P_{2}}\right\|}_{1}\leq \epsilon^{2}+\sum_{h_{1},h_{\sigma }<H_{n}}|\pi_{h_{1}}^{\left(1\right)}\pi_{\sigma ,h_{\sigma }}^{\left(1\right)}-\pi_{h_{1}}^{\left(2\right)}\pi_{\sigma ,h_{\sigma }}^{\left(2\right)}|+\epsilon +\sum_{h_{1},h_{\sigma }\leq H_{n}}\pi_{h_{1}}^{\left(1\right)}\pi_{\sigma ,h_{\sigma }}^{\left(1\right)}{\left\|\phi_{\Sigma_{h_{\sigma }}^{\left(1\right)}}\left(x-\mu_{h_{1}}^{\left(1\right)}\right)-\phi_{\Sigma_{h_{\sigma }}^{\left(2\right)}}\left(x_{t}-\mu_{h_{1}}^{\left(2\right)}\right)\right\|}_{1}$, where $\|\cdot {\|}_{1}$ denotes the $L_{1}$ norm. Using similar steps as in the proof of Lemma 2 in Canale and De Blasi (2017), it is possible to show that ${\left\|\phi_{\Sigma_{h_{\sigma }}^{\left(1\right)}}\left(x-\mu_{h_{1}}^{\left(1\right)}\right)-\phi_{\Sigma_{h_{\sigma }}^{\left(2\right)}}\left(x_{t}-\mu_{h_{1}}^{\left(2\right)}\right)\right\|}_{1}\leq \sqrt{\frac{2}{\pi }}\|\frac{\mu_{h_{1}}^{\left(1\right)}-\mu_{h_{1}}^{\left(2\right)}\|}{\sqrt{\lambda_{D}\left(\Sigma_{h_{\sigma }}^{\left(2\right)}\right)}}$ $+{\left\{\sum_{k=1}^{D}\frac{\lambda_{k}\left(\Sigma_{h_{\sigma }}^{\left(1\right)}\right)}{\lambda_{k}\left(\Sigma_{h_{\sigma }}^{\left(2\right)}\right)}-log\frac{\lambda_{k}\left(\Sigma_{h_{\sigma }}^{\left(1\right)}\right)}{\lambda_{k}\left(\Sigma_{h_{\sigma }}^{\left(2\right)}\right)}-1\right\}}^{1/2}+\left\{2D{\left\|O_{h_{\sigma }}^{\left(1\right)}-O_{h_{\sigma }}^{\left(2\right)}\right\|}_{2}\frac{\lambda_{1}\left(\Sigma_{h_{\sigma }}^{\left(1\right)}\right)}{\lambda_{D}\left(\Sigma_{h_{\sigma }}^{\left(2\right)}\right)}\right\}$, where $O\left(j^{\prime }\right)$ represents the matrix of orthogonal vectors in the spectral decomposition of $\Sigma^{\left(j^{\prime }\right)},j^{\prime }=1,2$. Finally, we need to establish an upper bound for the term $\sum_{h_{1},h_{\sigma }<H_{n}}\left|\pi_{h_{1}}^{\left(1\right)}\pi_{\sigma ,h_{\sigma }}^{\left(1\right)}-\pi_{h_{1}}^{\left(2\right)}\pi_{\sigma ,h_{\sigma }}^{\left(2\right)}\right|$, which is given by Lemma 5 as $\sum_{h_{1},h_{\sigma }<H_{n}}\left|{\tilde{\pi }}_{h_{1}}^{\left(1\right)}{\tilde{\pi }}_{\sigma ,h_{\sigma }}^{\left(1\right)}-\pi_{h_{1}}^{\left(2\right)}\pi_{\sigma ,h_{\sigma }}^{\left(2\right)}\right|+\left|1-(1-\epsilon {)}^{2}\right|$, where $\tilde{\pi }=\frac{\pi_{h}}{\left(1-\sum_{h>H}\pi_{h}\right)}$.

For a given $f_{P}\in {ℱ}_{n,\mathrm{jl}}$ with $P=\underset{h_{1}\geq 1}{\sum }\underset{h_{\sigma }\geq 1}{\sum }\pi_{h_{1}}^{\left(1\right)}\pi_{\sigma ,h_{\sigma }}^{\left(1\right)}\delta_{\left(\Theta_{h_{1}}^{\left(1\right)},\Sigma_{h_{\sigma }}^{\left(1\right)}\right)}$ and $\Sigma_{h}={\left(O_{h}\Lambda_{h}O_{h}^{T}\right)}^{-1}$ where $\Lambda_{h}=\mathrm{diag}\left(\lambda_{h,1},\ldots ,\lambda_{h,D}\right)$, we will construct another density, $f_{\overset{ˆ}{P}}$, where $\overset{ˆ}{P}=\underset{h_{1}\geq 1}{\sum }\underset{h_{\sigma }\geq 1}{\sum }\pi_{h_{1}}^{\left(1\right)}\pi_{\sigma ,h_{\sigma }}^{\left(1\right)}\delta_{\left({\overset{ˆ}{\Theta }}_{h_{1}}^{\left(1\right)},{\overset{ˆ}{\Sigma }}_{h_{\sigma }}^{\left(1\right)}\right)}$ within the ϵ-net and then compute the cardinality of the ϵ-net set to derive an upper bound for the entropy of sieve ${ℱ}_{n,\mathrm{jl}}$. To construct such a density, we will choose:

1. ${\overset{ˆ}{\mu }}_{h_{1}}\in {\overset{ˆ}{ℛ}}_{h_{1}},h_{1}=1,\ldots ,H$, where ${\overset{ˆ}{ℛ}}_{h_{1}}$ is a $\epsilon^{*}$-net of ${ℛ}_{h_{1}}:=\left\{\mu \in {ℜ}^{D}:{\underline{\mu }}_{h_{1},j}\leq \|\mu \|\leq \right.$ $\left.{\overset{‾}{\mu }}_{h_{1},j}\right\}$, such that $\left\|\mu_{h_{1}}-{\overset{ˆ}{\mu }}_{h_{1}}\right\|\leq {\underline{\sigma }}_{n}\epsilon ,k=1,\ldots ,K$, where $\overset{‾}{\mu },\underline{\mu }$, and ${\underline{\sigma }}_{n}$ correspond to the sieve boundaries in (16).
2. $\left\{{\overset{ˆ}{\pi }}_{h_{1}}{\overset{ˆ}{\pi }}_{h_{\sigma }},h_{1},h_{\sigma }\leq H_{n}\right\}\in \overset{ˆ}{\Delta }$, where $\overset{ˆ}{\Delta }$ is a ϵ-net of a $H_{n}^{2}$ dimensional probability simplex such that $\sum_{h_{1},h_{\sigma }\leq H_{n}}\left|{\tilde{\pi }}_{h_{1}}{\tilde{\pi }}_{h_{\sigma }}-{\overset{ˆ}{\pi }}_{h_{1}}{\overset{ˆ}{\pi }}_{h_{\sigma }}\right|\leq \epsilon$, and ${\tilde{\pi }}_{h}=\frac{\pi_{h}}{\sum_{h\leq H_{n}}\pi_{h}},h\leq H_{n}$.
3. ${\overset{ˆ}{O}}_{h}\in {\overset{ˆ}{𝒪}}_{h}$, where ${\overset{ˆ}{𝒪}}_{h}$ is a $\delta_{h}$-net of the set ${𝒪}_{h}$ defined as the set of D×D orthogonal matrices with respect to the spectral norm $\|\cdot {\|}_{2}$ with $\delta_{h}=\epsilon^{2}/\left(2Du_{h,l}\right)$ such that $\|O_{h}-{\overset{ˆ}{O}}_{h}{\|}_{2}\leq T\delta_{h}$.
4. $\left(m_{h,1},\ldots ,m_{h_{D}}\right)\in \{1,\ldots ,M{\}}^{D},h=1,\ldots ,H$, such that ${\overset{ˆ}{\lambda }}_{h,l}={\left\{{\underline{\sigma }}^{2}(1+\epsilon \sqrt{D}{)}^{m_{h,l}-1}\right\}}^{-1}$ will satisfy $1\leq {\overset{ˆ}{\lambda }}_{h,l}/\lambda_{h,l}<(1+\epsilon /\sqrt{D})$.

Under this construction, it can be shown that ${\left\|f_{P}-f_{\overset{ˆ}{P}}\right\|}_{1}<C^{*}\epsilon$ for some constant $C^{*}$, by employing some additional algebra and similar arguments as in the proof of Lemma 2 in Canale and De Blasi (2017) and Theorem 4 in our paper. Further, the cardinality of the ϵ-net can be computed by noting that $\#(\overset{ˆ}{\Delta })≲\epsilon^{-H_{n}^{2}}$ for $j=1,2,\#\left({\overset{ˆ}{𝒪}}_{h}\right)≲\delta_{h}^{-D(D-1)/2},\#\left({\overset{ˆ}{ℛ}}_{k,h}\right)≲$ $\left[{\left(\frac{{\overset{‾}{a}}_{h}}{\epsilon^{*}}+1\right)}^{D}-{\left(\frac{a_{h}}{\epsilon^{*}}-1\right)}^{D}\right]$. Using these quantities, one can write the upper bound for the exponential of the entropy bound as,

$${\left(M\right)}^{DH_{n}}\epsilon^{-H_{n}^{2}}\times \underset{h_{1}\leq H_{n}}{\prod }\left\{{\left(\frac{{\bar{a}}_{h_{1},j}}{{\underline{\sigma }}_{n}\epsilon }+1\right)}^{D}-{\left(\frac{{\underline{a}}_{h_{1},j}}{{\underline{\sigma }}_{n}\epsilon }-1\right)}^{D}\right\}\underset{h_{\sigma }\leq H_{n}}{\prod }{\left\{\frac{2Du_{h_{\sigma },l}}{\epsilon^{2}}\right\}}^{D(D-1)/2}\;\approx \exp\left\{DH_{n}\log(M)+H_{n}^{2}\log\left(\frac{1}{\epsilon }\right)+\frac{D(D-1)}{2}\log\left(n^{l_{h_{\sigma }}}\right)+\frac{D(D-1)}{2}\log\left(\frac{1}{\epsilon }\right)\right\},$$

when n and $j_{h_{1}}$ is large, and using the definitions of $\underline{a}$ and $\overset{‾}{a}$ defined in (16), and following similar steps as in the proof of Theorem 2 in Canale and De Blasi (2017) to show that $\left[{\left(\frac{{\overset{‾}{a}}_{h_{1},j}}{{\underline{\sigma }}_{n}\epsilon /2}+1\right)}^{D}-{\left(\frac{{\underline{a}}_{h_{1},j}}{{\underline{\sigma }}_{n}\epsilon /2}-1\right)}^{D}\right]≲\left[\frac{n^{\left(H_{n}+\frac{1}{2c_{2}}\right)D}j_{h1}^{D-1}}{(\epsilon {)}^{D}}\right]$. Further, using similar steps as in (33) in the proof of Theorem 5, we have $\Pi \left({ℱ}_{n,\mathrm{jl}}\right)\leq \underset{h_{1}\leq H_{n}}{\prod }P_{1}^{*}\left(\left\|\mu_{h_{1}}\right\|>n^{H_{n}^{2}}\left(j_{h_{1}}-1\right)\right)$ $\prod_{h_{\sigma }\leq H_{n}}P_{2}^{*}\left(\lambda_{1}(\Sigma )/\lambda_{D}(\Sigma )>n^{\left(l_{h_{\sigma }}-1\right)}\right)≲\prod_{h_{1}\leq H_{n}}\left\{{\left(n^{H_{n}^{2}}\left(j_{h_{1}}-1\right)\right)}^{-1_{\left(j_{h_{1}}\geq 2\right)}2(r+1)}\right\}$ $\times \prod_{h_{\sigma }\leq H_{n}}{\left(n^{\left(l_{h_{\sigma }}-1\right)}\right)}^{-1_{\left(l_{h_{\sigma }}\geq 1\right)}\kappa }\approx \left\{n^{-2H_{n}^{3}(r+1)}\prod_{h_{1}\leq H_{n}}{\left(j_{h_{1}}-1\right)}^{-1_{\left(j_{h_{1}}\geq 2\right)}2(r+1)}\right\}$ $\times \left\{\prod_{h_{\sigma }\leq H_{n}}{\left(n^{\kappa \left(l_{h_{\sigma }}-1\right)}\right)}^{{-1}_{\left(l_{h_{\sigma }}\geq 1\right)}}\right\}$, for large n.

Finally, using similar steps as in Lemma 7 in the Appendix it is possible to show that the summability condition in Theorem 2 holds. This proves the strong consistency result for the product mixture of DP priors for multivariate density estimation.

**Proof of Theorem 3:** We will use the conditions in Lemmas 2-4 and Theorem 2 in Wu and Ghosal (2008) to prove our results. Note that $f_{0}(X)=\prod_{t=1}^{T}f_{0}\left(x_{t}|X_{1:(t-1)}\right)$, where $f_{0}\left(x_{t}|X_{1:(t-1)}\right)=f_{0}\left(x_{1}\right)$ for t=1 by convention. As a shorthand notation, we will denote $\frac{f_{0}(x)}{f_{P}(x)}=\left(f_{0}/f_{P}\right)(x)$ in the following proof. For any P∈𝒫, note that the KL divergence can be expressed as $KL\left(f_{0}(X),f_{P}(X)\right)=\int \;f_{0}(X)log\left(\left(f_{0}/f_{P}\right)(X)\right)dx_{1}\ldots dx_{T}$

(17)
$$=\int \overset{T}{\underset{t=1}{\prod }}f_{0}\left(x_{t}|X_{1:(t-1)}\right)\log\left(\overset{T}{\underset{t=1}{\prod }}\left(f_{0}/f_{P}\right)\left(x_{t}|X_{1:(t-1)}\right)\right)dx_{1}\ldots dx_{T}\;=\int \overset{T}{\underset{t=1}{\prod }}f_{0}\left(x_{t}|X_{1:(t-1)}\right)\times \left\{\overset{T}{\underset{t=1}{\sum }}\log\left(\left(f_{0}/f_{P}\right)\left(x_{t}|X_{1:(t-1)}\right)\right)\right\}dx_{1}\ldots dx_{T}\;=\overset{T}{\underset{t=1}{\sum }}[\underset{t^{*}>t}{\prod }\int \underset{=1}{\underset{︸}{f_{0}\left(x_{t^{*}}|X_{1:\left(t^{*}-1\right)}\right)dx_{t^{*}}}}\times \int \{\int f_{0}\left(x_{t}|X_{1:(t-1)}\right)\log\left(\left(f_{0}/f_{P}\right)\left(x_{t}|X_{1:(t-1)}\right)\right)dx_{t}\;\times \underset{t^{\prime }<t}{\prod }f_{0}\left(x_{t^{\prime }}|X_{1:\left(t^{\prime }-1\right)}\right)dx_{t^{\prime }}\}]\;=\overset{T}{\underset{t=1}{\sum }}\left\{\int KL\left(f_{0}\left(x_{t}|X_{1:(t-1)}\right),f_{P}\left(x_{t}|X_{1:(t-1)}\right)\right)\overset{t^{\prime }=1}{\underset{t-1}{\prod }}f_{0}\left(x_{t^{\prime }}|X_{1:\left(t^{\prime }-1\right)}\right)dx_{t^{\prime }-1}\ldots dx_{1}\right\},$$

which is a sum of integrals involving Kullback-Leibler divergences of conditional densities. In the following derivations, we will use the shorthand notation $KL_{\left(f_{0},f_{P}\right)}\left(x_{t}|X_{1:(t-1)}\right)$ to denote $KL\left(f_{0}\left(x_{t}|X_{1:(t-1)}\right),f_{P}\left(x_{t}|X_{1:(t-1)}\right)\right)$ where convenient. We will prove that $KL_{\left(f_{0},f_{P}\right)}\left(x_{t}|X_{1:(t-1)}\right)$ is infinitesmall (<ϵ) with positive probability pointwise for $X_{1:(t-1)}$ for t=1,…,T, which will imply that the above sum on the right hand side of the equality also becomes infinitesmall for fixed T, and hence we will prove our Theorem.

As a first step, we will define $P_{\epsilon }$ on a compact set $\left\{\Theta \;:\;-a\leq A_{k}\left(j,j^{\prime }\right)\leq a,k=1,\ldots ,K,\;\text{and}\;\left\|\sum_{k=1}^{K}A_{k}x_{t-k}\right\|<m\right\}\times \left\{\Sigma \in 𝒮\;:\;h_{m}=m^{-\eta }\leq \lambda_{D}(\Sigma )<\ldots <\lambda_{1}(\Sigma )\leq \overset{¯}{M},t=1,\ldots ,T\right\}={𝒟}_{1}^{*}\times {𝒟}_{2}^{*}$, such that $P_{\epsilon }\left({𝒟}_{1}^{*}\times {𝒟}_{2}^{*}\right)=1$, for some m,η>0. We will construct $P_{\epsilon }$ such that it ensures that the upper bounds for the terms in the right hand side of the above equality are arbitrarily small with positive prior probability:

(18)
$$KL_{\left(f_{0},f_{P}\right)}\left(x_{t}|X_{1:(t-1)}\right)=KL_{\left(f_{0},f_{P_{\epsilon }}\right)}\left(x_{t}|X_{1:(t-1)}\right)+KL_{\left(f_{P_{\epsilon }},f_{P}\right)}\left(x_{t}|X_{1:(t-1)}\right).$$

Write $r=\sum_{k}A_{k}x_{t-k}$, and $t_{m}^{-1}=\int_{\|r\|<m}f_{0}\left(r|X_{1:(t-1)}\right)d(r)$ and define $t_{m}^{-1}f_{P_{\epsilon }}\left(x_{t}|X_{1:(t-1)}\right)$

$$=\int_{\|r\|<m}\phi_{\text{Σ}}\left(x_{t}-r|X_{1:(t-1)}\right)f_{0}\left(r_{t-k}|X_{1:(t-1)}\right)d(r)\geq \int_{\|r\|<m}\phi_{h_{m}I_{D}}\left(x_{t}-r|X_{1:(t-1)}\right)f_{0}\left(r|X_{1:(t-1)}\right)d(r)\;\times {\left(\frac{\lambda_{D}(\text{Σ})}{\lambda_{1}(\text{Σ})}\right)}^{(D-1)/2}=t_{m}\underset{\|x_{t}-\theta h_{m}\|<m}{\int }\;\phi_{h_{m}I_{D}}\left(\theta |X_{1:(t-1)}\right)f_{0}\left(x_{t}-\theta h_{m}|X_{1:(t-1)}\right)d\theta \times {\left(\frac{\lambda_{D}(\text{Σ})}{\lambda_{1}(\text{Σ})}\right)}^{(D-1)/2}$$

where $\theta =\left(x_{t}-\sum_{k=1}^{K}A_{k}x_{t-k}\right)/h_{m}$, and the inequality in the second last step is derived using the fact that ${\left(\frac{\lambda_{D}(\Sigma )}{\lambda_{1}(\Sigma )}\right)}^{(D-1)/2}\phi_{\lambda_{D}(\Sigma )I_{D}}(x)\leq \phi_{\Sigma }(x)\leq {\left(\frac{\lambda_{1}(\Sigma )}{\lambda_{D}(\Sigma )}\right)}^{(D-1)/2}\phi_{\lambda_{1}(\Sigma )I_{D}}(x)$. Using the above, we have $KL_{\left(f_{0},f_{P_{\epsilon }}\right)}\leq {\left(\frac{\lambda_{1}(\Sigma )}{\lambda_{D}(\Sigma )}\right)}^{(D-1)/2}KL_{\left(f_{0},f_{P_{\epsilon ,\Sigma =h_{m}I_{D}}}\right)}$, where $f_{P_{\epsilon ,\Sigma =h_{m}I_{D}}}$ has the same form as $f_{P_{\epsilon }}$ but with Σ set to $h_{m}I_{D}$. Hence, the next step is to show that $KL\left(f_{0},f_{P_{\epsilon ,\Sigma =h_{m}I_{D}}}\right)\left(x_{t}|X_{1:(t-1)}\right)$ can be made arbitrarily small with positive prior probability, point-wise for all $X_{1:(t-1)}$, which will help establish that the first term on the right hand side of (18) is negligible with positive prior probability.

Since when m→∞ and $h_{m}\to 0,\phi_{h_{m}I_{D}}\left(x_{t}-\sum_{k=1}^{K}A_{k}x_{t-k}|X_{1:(t-1)}\right)$ takes non-zero values only when $\sum_{k=1}^{K}A_{k}x_{t-k}\to x_{t}$, we obtain $\phi_{h_{m}I_{D}}\left(x_{t}-\sum_{k=1}^{K}A_{k}x_{t-k}|X_{1:(t-1)}\right)f_{0}\left(\sum_{k=1}^{K}A_{k}x_{t-k}|\right.$ $\left.X_{1:(t-1)}\right)\to f_{0}\left(x_{t}|X_{1:(t-1)}\right)$ as m→∞ and $h_{m}\to 0$, which also implies $f_{P_{\epsilon ,\Sigma =h_{m}I_{D}}}\left(x_{t}|\right.$ $\left.X_{1:(t-1)}\right)\to f_{0}\left(x_{t}|X_{1:(t-1)}\right)$, point-wise for all $X_{1:(t-1)}$. We will combine the above convergence with the fact the $log\left(\frac{f_{0}\left(x_{t}|X_{1:(t-1)}\right)}{f_{P_{\epsilon },\Sigma =h_{m}I_{D}}\left(x_{t}|X_{1:(t-1)}\right)}\right)$ is bounded and integrable (as shown in the sequel) and subsequently apply the DCT to achieve our result. As a next step, note $KL_{(f_{0},f_{P_{\epsilon ,\text{Σ}=h_{m}I_{D}})}}\left(x_{t}|X_{1:(t-1)}\right)=\int_{\left\|x_{t}\leq m\right\|}f_{0}\left(x_{t}|X_{1:(t-1)}\right)\log\left(\frac{f_{0}\left(x_{t}|X_{1:(t-1)}\right)}{f_{P_{\epsilon ,\Sigma =h_{m}I_{D}}}\left(x_{t}|X_{1:(t-1)}\right)}\right)dx_{t}+\int_{\left\|x_{t}>m\right\|}f_{0}\left(x_{t}|X_{1:(t-1)}\right)\log\left(\frac{f_{0}\left(x_{t}|X_{1:(t-1)}\right)}{f_{P_{\epsilon ,\Sigma =h_{m}I_{D}}}\left(x_{t}|X_{1:(t-1)}\right)}\right)dx_{t}$. Using similar arguments as in the Proof of Theorem 2 in Wu and Ghosal (2008), for $\left\|x_{t}\right\|>m$, we have $f_{P_{\epsilon ,\Sigma =h_{m}I_{D}}}\left(x_{t}|\right.$ $\left.X_{1:(t-1)}\right)$

$$\geq t_{m}\int_{\|\sum_{k}A_{k}x_{t-k}\|<m}\phi_{h_{m}I_{D}}\left(x_{t}+mx_{t}/\|x_{t}\||X_{1:(t-1)}\right)f_{0}\left(r|X_{1:(t-1)}\right)d\left(\overset{k=1}{\underset{K}{\sum }}A_{k}x_{t-k}\right)\;=\phi_{h_{m}I_{D}}\left(x_{t}+mx_{t}/\|x_{t}\|\right)\times \left\{\underset{=1}{\underset{︸}{t_{m}\int_{\|\sum_{k}A_{k}x_{t-k}\|<m}f_{0}\left(r|X_{1:(t-1)}\right)d\left(\overset{k=1}{\underset{K}{\sum }}A_{k}x_{t-k}\right)}}\right\}\;=\phi_{h_{m}I_{D}}\left(x_{t}+mx_{t}/\|x_{t}\|\right)=m^{\eta }\phi_{I_{D}}\left(m^{\eta }x_{t}+m^{1+\eta }x_{t}/\|x_{t}\|\right)\geq \|x_{t}{\|}^{\eta }\phi_{I_{D}}\left(2\|x_{t}\|x_{t}\right).$$

Similarly for $\left\|x_{t}\right\|\leq m$, it is possible to show that given a constant $\delta >0,f_{P_{\epsilon ,\Sigma =h_{m}I_{D}}}\left(x_{t}|\right.$ $\left.X_{1:(t-1)}\right)\geq c\underset{\|r-x_{t}\|<\delta }{inf}f_{0}\left(r|X_{1:(t-1)}\right)$, using similar steps as in the proof of Theorem 2 in Wu and Ghosal (2008). Hence for a given constant R<m, we have

(19)
$$\log\left(\frac{f_{0}\left(x_{t}|X_{1:(t-1)}\right)}{f_{P_{\epsilon ,\text{Σ}=h_{m}I_{D}}}\left(x_{t}|X_{1:(t-1)}\right)}\right)=\xi \left(x_{t};X_{1:(t-1)}\right)\leq \{\begin{array}{cc} r_{1}^{*}=\log\left(\frac{f_{0}\left(x_{t}|X_{1:(t-1)}\right)}{c_{\underset{\|r-x_{t}\|<\delta }{inf}}f_{0}\left(r|X_{1:(t-1)}\right)}\right), & \|x_{t}\|<R,\\ \max\left\{\log\left(\frac{f_{0}\left(x_{t}|X_{1:(t-1)}\right)}{\|x_{t}{\|}^{\eta }\phi_{I_{D}}\left(2\|x_{t}\|x_{t}\right)}\right),r_{1}^{*}\right\} & \|x_{t}\|\geq R. \end{array}$$

Further note that $f_{P_{\epsilon ,\Sigma =h_{m}I_{D}}}\left(x_{t}|X_{1:(t-1)}\right)\leq Mt_{1}$ which implies $\log\left(\frac{f_{0}\left(x_{t}|X_{1:(t-1)}\right)}{\left(f_{P_{\epsilon ,\Sigma =h_{m}I_{D}}\left(x_{t}|X_{1:(t-1)}\right)}\right)}\right.\geq \log\left(\frac{f_{0}\left(x_{t}|X_{1:(t-1)}\right)}{M\phi_{1}^{*}}\right)$, where $\phi_{1}^{*}=\underset{\left\|x_{t}\right\|<1}{\int }f_{0}\left(x_{t}|X_{1:(t-1)}\right)$ the lower bound is <0. Combining this fact with the upper bound in (19), it is possible to write

$$KL\left(\frac{f_{0}\left(x_{t}|X_{1:(t-1)}\right)}{f_{P_{\epsilon ,\Sigma =h_{m}I_{D}}}\left(x_{t}|X_{1:(t-1)}\right)}\right)\leq \int \;f_{0}\left(x_{t}|X_{1:(t-1)}\right)max\left\{\xi \left(x_{t};X_{1:(t-1)}\right),\left|log\left(\frac{f_{0}}{M\phi_{1}^{*}}\right)\right|\right\}.$$

Using assumptions *(A2)-(A4)* and similar steps as in the proof of Theorem 2 in Wu and Ghosal (2008), it is possible to show that the RHS is bounded, point-wise for all $X_{1:(t-1)}$. Hence using DCT, the term $KL_{\left(f_{0},f_{P_{\epsilon },\Sigma =h_{m}I_{D}}\right)}\left(x_{t}|X_{1:(t-1)}\right)$ can be made arbitrarily small with positive prior probability. Therefore, we see that for any ϵ>0, there exists $m_{\epsilon }$ such that $KL_{\left(f_{0},f_{P_{\epsilon }}\right)}\left(x_{t}|X_{1:(t-1)}\right)\leq {\left(\frac{\lambda_{1}(\Sigma )}{\lambda_{D}(\Sigma )}\right)}^{(D-1)/2}KL_{\left(f_{0},f_{P_{\epsilon ,\Sigma =h_{m}I_{D}}}\right)}\left(x_{t}|X_{1:(t-1)}\right)\leq \epsilon /2$. Hence the first term in (18) is bounded by ϵ/2 with positive prior probability.

To show that the second term in (18) is negligible, we will demonstrate that the conditions in Lemma 3 of Wu and Ghosal (2008) are satisfied. Using similar arguments as in Wu and Ghosal (2008) as well as the proof of Lemma 1 in Canale and De Blasi (2017), it is possible to show that the weak support of $\Pi^{*}$ contains any compactly supported 𝒫. Since $P_{\epsilon }$ is compactly supported by definition, it belongs to the weak support of $\Pi^{*}$. Next, condition (A7) of Lemma 3 in Wu and Ghosal (2008) requires $log\left(f_{P_{\epsilon }}\right)$ and $log\underset{A_{1},\ldots ,A_{K},\Sigma }{inf}\phi_{\Sigma }\left(x_{t}-\sum_{k=1}^{K}A_{k}x_{t-k}\right)$ to be $f_{0}-$ integrable. Note that for $\left\|x_{t}\right\|<m$, $log\underset{A_{1},\ldots ,A_{K},\Sigma }{inf}\phi_{\Sigma }\left(x_{t}-\sum_{k=1}^{K}A_{k}x_{t-k}\right)$ is bounded, and for $\left\|x_{t}\right\|>m,\underset{A_{1},\ldots ,A_{K},\Sigma }{inf}\phi_{\Sigma }\left(x_{t}-\right.$ $\left.\sum_{k=1}^{K}A_{k}x_{t-k}\right)\leq {\overset{‾}{M}}^{-D}\phi \left(exp\left\{-\frac{4{\left\|x_{t}\right\|}^{2}}{2m^{-\eta }}\right\}\right)$, which is $f_{0}$ integrable. A similar upper bound can be applied for bounding $\left|log\left(f_{P_{\epsilon }}\right)\right|$ for $\left\|x_{t}\right\|>m$ that implies that $\left|log\left(f_{P_{\epsilon }}\right)\right|$ is $f_{0}$ integrable, which satisfies condition (A7) in Lemma 3 of Wu and Ghosal (2008). Note that the above statements hold point-wise for all $X_{1:(t-1)}$.

Condition (A8) of Lemma 3 in Wu and Ghosal (2008) corresponding to the second term in (18) is clearly satisfied since the multivariate normal kernel $\phi_{\Sigma }\left(x_{t}-\sum_{k=1}^{K}A_{k}x_{t-k}\right)$ is bounded away from zero for $x_{t}$ in a compact set of ${ℜ}^{d}$ and $\left(A_{1},\ldots ,A_{K},\Sigma \right)\in {𝒟}_{1}^{*}\times {𝒟}_{2}^{*}$. To show condition (A9) in Lemma 3 in Wu and Ghosal (2008), we will need to show that the kernel $\phi_{\Sigma }\left(x_{t}-\sum_{k=1}^{K}A_{k}x_{t-k}\right)$ is equicontinuous as a family of functions of $\left\{A_{1},\ldots ,A_{K},\Sigma \right\}$ for $x_{t}$ lying on a compact subset of ${ℜ}^{D}$ and conditional on given values of $X_{1:(t-1)}$. Note that for two distinct sets of parameters (Θ,Σ) and $\left(\Theta^{\prime },\Sigma^{\prime }\right)$,

(20)
$$\left|\phi_{\text{Σ}}\left(x_{t}-\overset{K}{\underset{k=1}{\Sigma }}A_{k}x_{t-k}\right)-\phi_{{\text{Σ}}_{t}^{\prime }}\left(x_{t}-\overset{K}{\underset{k=1}{\Sigma }}A_{k}^{\prime }x_{t-k}\right)\right|\leq |\phi_{{\text{Σ}}^{\prime }}\left(x_{t}-\overset{K-1}{\underset{k=1}{\Sigma }}A_{k}x_{t-k}-A_{K}^{\prime }x_{t-K}\right)-\phi_{\text{Σ}}\left(x_{t}-\overset{K}{\underset{k=1}{\Sigma }}A_{k}x_{t-k}\right)|+\left|\phi_{{\text{Σ}}^{\prime }}\left(x_{t}-\overset{K-1}{\underset{k=1}{\Sigma }}A_{k}x_{t-k}-A_{K}^{\prime }x_{t-K}\right)-\phi_{{\text{Σ}}^{\prime }}\left(x_{t}-\overset{K}{\underset{k=1}{\Sigma }}A_{k}^{\prime }x_{t-k}\right)\right|.$$

The first term in (20) can be shown to be arbitrarily small when $\left(A_{K}^{\prime },\Sigma^{\prime }\right)$ lies within a small neighborhood of $\left(A_{K},\Sigma \right)$ (or equivalently $A_{K}x_{t-K}$ is in a neighborhood of $A_{K}^{\prime }x_{t-K}$ pointwise for $x_{t-K}$, and Σ lies in a neighborhood of $\Sigma^{\prime }$) for $x_{t}\in C\subset {ℜ}^{D}$, where C is a compact subset of ${ℜ}^{D}$, using arguments similar to the last part of the proof of Theorem 2 in Wu and Ghosal (2008). The second term in (20) can be decomposed using similar and repeated iterative steps, and hence shown to be arbitrarily small. Hence the equicontinuity condition holds, and all conditions of Lemma 3 in Wu and Ghosal (2008) are satisfied, pointwise for all $X_{1:(t-1)}$. This implies that the second term in (18) is less than or equal to ϵ/2. Combining this with the upper bound on the first term in (18), it is possible to show that $KL\left(f_{0},f_{P_{\epsilon }}\right)\left(x_{t}|X_{1:(t-1)}\right)<\epsilon$, point-wise almost everywhere for $X_{1:(t-1)}$. Combining the above results and using the expression in (17), we have $KL\left(f_{0}(X),f(X)\right)\leq \epsilon$. Finally, we note that this bound holds with positive prior probability under the PDPM-VAR, lgPDPM-VAR and rgPDPM-VAR models, thus yielding the desired result in Theorem 3.

**Proof of Theorem 4:** The proof of this result is based on modifications of the arguments in the proof of Proposition 2 in Shen et al. (2013) and Lemma 2 in Canale and De Blasi (2017). We will show that for every $f_{P}\in {ℱ}_{n,jl}$, it is possible to find another density $f_{\overset{ˆ}{P}}$ belonging to $\overset{ˆ}{𝒢}$ (the ϵ-net over ${ℱ}_{n,jl}$) such that ${\left\|f_{P}-f_{\overset{ˆ}{P}}\right\|}_{1}\leq \epsilon$. Since $N\left(\epsilon ,{ℱ}_{n,jl},\|\cdot {\|}_{1}\right)$ is the minimum cardinality of the ϵ-net over ${ℱ}_{n,jl}$, we will be able to obtain a desired upper bound on the entropy if the number of balls required to cover the ϵ-net $\overset{ˆ}{𝒢}$ is bounded. Let us consider $P_{1}=\underset{h_{1}\geq 1}{\sum }\underset{h_{\sigma }\geq 1}{\sum }\pi_{h_{1}}^{\left(1\right)}\pi_{\sigma ,h_{\sigma }}^{\left(1\right)}\delta_{\left(\Theta_{h_{1}}^{\left(1\right)},\Sigma_{h_{\sigma }}^{\left(1\right)}\right)}$ and $P_{2}=\sum_{h_{1}\geq 1}\sum_{h_{\sigma }\geq 1}\pi_{h_{1}}^{\left(2\right)}\pi_{\sigma ,h_{\sigma }}^{\left(2\right)}\delta_{\left(\Theta_{h_{1}},\Sigma_{h_{\sigma }}^{\left(2\right)}\right)}$. Using Lemma 4 (elaborated in the sequel), the distance between the corresponding densities can be expressed as

(21)
$$\|f_{P_{1}}-f_{P_{2}}{\|}_{1}\leq 2\epsilon^{2}+\underset{h_{1},h_{\sigma }<H_{n}}{\Sigma }\left|\pi_{h_{1}}^{\left(1\right)}\pi_{\sigma ,h_{\sigma }}^{\left(1\right)}-\pi_{h_{1}}^{\left(2\right)}\pi_{\sigma ,h_{\sigma }}^{\left(2\right)}\right|+4\epsilon \;+\sum_{h_{1},h_{\sigma }\leq H_{n}}\pi_{h_{1}}^{\left(1\right)}\pi_{\sigma ,h_{\sigma }}^{\left(1\right)}\|\prod_{t=1}^{T}\phi_{{\text{Σ}}_{h_{\sigma }}^{\left(1\right)}}\left(x_{t}-\sum_{k=1}^{K}A_{k,h_{1}}^{\left(1\right)}x_{t-k}\right)-\prod_{t=1}^{T}\phi_{{\text{Σ}}_{h_{\sigma }}^{\left(2\right)}}{\left(x_{t}-\sum_{k=1}^{K}A_{k,h_{1}}^{\left(2\right)}x_{t-k}\right)}_{1}\|$$

Let us first investigate the upper bound for the last term on the right hand side of (21). Note that

(22)
$$\|\prod_{t=1}^{T}\phi_{{\text{Σ}}_{h_{\sigma }}^{\left(1\right)}}\left(x_{t}-\sum_{k=1}^{K}A_{k,h_{1}}^{\left(1\right)}x_{t-k}\right)-\prod_{t=1}^{T}\phi_{{\text{Σ}}_{h_{\sigma }}^{\left(2\right)}}{\left(x_{t}-\sum_{k=1}^{K}A_{k,h_{1}}^{\left(2\right)}x_{t-k})\right\|}_{1}\;\leq \|\prod_{t=1}^{T}\phi_{{\text{Σ}}_{h_{\sigma }}^{\left(2\right)}}\left(x_{t}-\sum_{k=1}^{K}A_{k,h_{1}}^{\left(1\right)}x_{t-k}\right)-\prod_{t=1}^{T}\phi_{{\text{Σ}}_{h_{\sigma }}^{\left(2\right)}}{\left(x_{t}-\sum_{k=1}^{K}A_{k,h_{1}}^{\left(2\right)}x_{t-k})\right\|}_{1}\;+\|\prod_{t=1}^{T}\phi_{{\text{Σ}}_{h_{\sigma }}^{\left(1\right)}}\left(x_{t}-\sum_{k=1}^{K}A_{k,h_{1}}^{\left(1\right)}x_{t-k}\right)-\prod_{t=1}^{T}\phi_{{\text{Σ}}_{h_{\sigma }}^{\left(2\right)}}{\left(x_{t}-\sum_{k=1}^{K}A_{k,h_{1}}^{\left(1\right)}x_{t-k})\right\|}_{1},$$

using the triangle inequality. The first term on the right hand side of (22) can be bounded above using the following steps. In particular, the first term is ≤

(23)
$$\|\left\{\phi_{{\text{Σ}}_{h_{\sigma }}^{\left(2\right)}}\left(x_{T}-\sum_{k=1}^{K}A_{k,h_{1}}^{\left(1\right)}x_{T-k}\right)-\phi_{{\text{Σ}}_{h_{\sigma }}^{\left(2\right)}}\left(x_{T}-\sum_{k=1}^{K}A_{k,h_{1}}^{\left(2\right)}x_{T-k}\right)\right\}\prod_{t=1}^{T-1}\phi_{{\text{Σ}}_{h_{\sigma }}^{\left(2\right)}}\left(x_{t}-\sum_{k=1}^{K}A_{k,h_{1}}^{\left(1\right)}x_{t-k}\right)|{|}_{1}\;+\|\phi_{{\text{Σ}}_{h_{\sigma }}^{\left(2\right)}}\left(x_{T}-\sum_{k=1}^{K}A_{k,h_{1}}^{\left(2\right)}x_{T-k}\right){\left\{\prod_{t=1}^{T-1}\phi_{{\text{Σ}}_{h_{\sigma }}^{\left(2\right)}}\left(x_{t}-\sum_{k=1}^{K}A_{k,h_{1}}^{\left(1\right)}x_{t-k}\right)-\prod_{t=1}^{T-1}\phi_{{\text{Σ}}_{h_{\sigma }}^{\left(2\right)}}\left(x_{t}-\sum_{k=1}^{K}A_{k,h_{1}}^{\left(2\right)}x_{t-k}\right)\right\}}_{1}\|.$$

The first term in (23) may be written as $\int \left\{\int |\left\{\phi_{\Sigma_{h_{\sigma }}^{\left(2\right)}}\left(x_{T}-\sum_{k=1}^{K}A_{k,h_{1}}^{\left(1\right)}x_{T-k}\right)-\phi_{\Sigma_{h_{\sigma }}^{\left(2\right)}}\left(x_{T}-\right.\right.\right.$ $\left.\left.\left.\sum_{k=1}^{K}A_{k,h_{1}}^{\left(2\right)}x_{T-k}\right)\right\}|dx_{T}\right\}\prod_{t=1}^{T-1}\phi_{\Sigma_{h_{\sigma }}^{\left(2\right)}}\left(x_{t}-\sum_{k=1}^{K}A_{k,h_{1}}^{\left(1\right)}x_{t-k}\right)dx_{T-1}\ldots dx_{1}$, which is $\leq \frac{2}{\pi \sqrt{\lambda_{D}\left(\Sigma_{h_{\sigma }}^{\left(2\right)}\right)}}\times$ $\int \ldots \int \left\{\left\|\sum_{k=1}^{K}\left(A_{k,h_{1}}^{\left(1\right)}-A_{k,h_{1}}^{\left(2\right)}\right)x_{T-k}\right\|\times \prod_{t=1}^{T-1}\phi_{\Sigma_{h_{\sigma }}^{\left(2\right)}}\left(x_{t}-\sum_{k=1}^{K}A_{k,h_{1}}^{\left(1\right)}x_{t-k}\right)\right\}dx_{T-1}\ldots dx_{1}$, where we have used the well-known result ${\left\|\phi_{\Sigma }\left(x_{T}-\mu_{1}\right)-\phi_{\Sigma }\left(x_{T}-\mu_{2}\right)\right\|}_{1}\leq \frac{2}{\pi \sqrt{\lambda_{D}(\Sigma )}}\|\mu_{1}-$$\mu_{2}\|$. One can write $\left\|\sum_{k=1}^{K}\left(A_{k,h_{1}}^{\left(1\right)}-A_{k,h_{1}}^{\left(2\right)}\right)x_{T-k}\right\|=\sqrt{\sum_{k=1}^{K}\sum_{l=1}^{D}{\left\{\sum_{l^{\prime }=1}^{D}\left(A_{k,h_{1}}^{\left(1\right)}\left(l,l^{\prime }\right)-A_{k,h_{1}}^{\left(2\right)}\left(l,l^{\prime }\right)\right)x_{T-k,l^{\prime }}\right\}}^{2}}$ $\leq \sqrt{\sum_{k=1}^{K}\sum_{l=1}^{D}\left(\sum_{l^{\prime }=1}^{D}{\left(A_{k,h_{1}}^{\left(1\right)}\left(l,l^{\prime }\right)-A_{k,h_{1}}^{\left(2\right)}\left(l,l^{\prime }\right)\right)}^{2}\right){\left\|x_{T-k}\right\|}^{2}}=\sqrt{\sum_{k=1}^{K}{\left\|\mathrm{vec}\left(A_{k,h_{1}}^{\left(1\right)}-A_{k,h_{1}}^{\left(2\right)}\right)\right\|}^{2}\times {\left\|x_{T-k}\right\|}^{2}}$$\leq \sum_{k=1}^{K}\left\|\mathrm{vec}\left(A_{k,h_{1}}^{\left(1\right)}-A_{k,h_{1}}^{\left(2\right)}\right)\right\|\times \left\|x_{T-k}\right\|$, where the second to last inequality is obtained using Cauchy-Schwarz inequality, and the last inequality uses the fact $\sum_{k=1}^{K}{\left(a^{*}\right)}_{k}^{2}\leq {\left(\sum_{k=1}^{K}{\left|a^{*}\right|}_{k}\right)}^{2}$. Hence, the first term in (23) has an upper bound $2{\left(\pi \sqrt{\lambda_{D}\left(\Sigma_{h_{\sigma }}^{\left(2\right)}\right)}\right)}^{-1}\times {𝒦}^{*}$, where ${𝒦}^{*}=$ $\sum_{k=1}^{K}\left\|\mathrm{vec}\left(A_{k,h_{1}}^{\left(1\right)}-A_{k,h_{1}}^{\left(2\right)}\right)\right\|\int \left\{\left\|x_{T-k}\right\|\prod_{t=1}^{T-1}\phi_{\Sigma_{h_{\sigma }}^{\left(2\right)}}\left(x_{t}-\sum_{k^{\prime }=1}^{K}A_{k^{\prime },h_{1}}^{\left(1\right)}x_{t-k^{\prime }}\right)\right\}dx_{T-1}\ldots dx_{1}$. Using Cauchy-Schwarz, $\int \left\{\left\|x_{T-k}\right\|\prod_{t=1}^{T-1}\phi_{\Sigma_{h_{\sigma }}^{\left(2\right)}}\left(x_{t}-\sum_{k^{\prime }=1}^{K}A_{k^{\prime },h_{1}}^{\left(1\right)}x_{t-k^{\prime }}\right)\right\}dx_{T-1}\ldots dx_{1}\leq$ $\sqrt{\tilde{\zeta }}\times \sqrt{\zeta_{1:(T-k)}}$, where $\tilde{\zeta }=\int \prod_{t=T-k+1}^{T-1}{\left\{\phi_{\Sigma_{h_{\sigma }}^{\left(2\right)}}\left(x_{t}-\sum_{k^{\prime }=1}^{K}A_{k^{\prime },h_{1}}^{\left(1\right)}x_{t-k^{\prime }}\right)\right\}}^{2}dx_{T-1}\ldots dx_{T-k+1}$ and $\zeta_{1:(T-k)}=\int {\left\|x_{T-k}\right\|}^{2}{\left\{\prod_{t=1}^{T-k}\phi_{\Sigma_{h_{\sigma }}^{\left(2\right)}}\left(x_{t}-\sum_{k^{\prime }=1}^{K}A_{k^{\prime },h_{1}}^{\left(1\right)}x_{t-k^{\prime }}\right)\right\}}^{2}dx_{T-k}\ldots dx_{1}$.

Noting that $\phi_{\text{Σ}}(x)\leq {\left(\frac{\lambda_{1}(\text{Σ})}{\lambda_{D}(\text{Σ})}\right)}^{(D-1)/2}\phi_{\lambda_{1}(\text{Σ})I_{D}}(x)$ and the fact that $\left(\frac{\lambda_{1}\left({\text{Σ}}_{h_{\sigma }}^{\left(2\right)}\right)}{\lambda_{D}\left({\text{Σ}}_{h_{\sigma }}^{\left(2\right)}\right)}\right)\leq u_{h_{\sigma },l}$ for all densities belonging to ${ℱ}_{n,jl}$, one can write $\tilde{\zeta }\leq \int \left\{\prod_{t=T-k+1}^{T-1}{\left(u_{h_{\sigma },l}\right)}^{(D-1)/2}\phi_{\lambda_{D}\left(\Sigma_{h_{\sigma }}^{\left(2\right)}\right)I_{D}}\left(x_{t}-\right.\right.$ ${\left.\left.\sum_{k^{\prime }=1}^{K}A_{k^{\prime },h_{1}}^{\left(1\right)}x_{t-k^{\prime }}\right)\right\}}^{2}dx_{T-1}\ldots dx_{T-k+1}=\tilde{\zeta^{*}}\times \frac{{\left(u_{h_{\sigma },l}\right)}^{(D-1)k}}{\lambda_{D}^{Dk/2}\left(\Sigma_{h_{\sigma }}^{\left(2\right)}\right)}\int \left\{\prod_{t=T-k+1}^{T-1}\phi_{\frac{1}{2}\lambda_{D}\left(\Sigma_{h_{\sigma }}^{\left(2\right)}\right)I_{D}}\left(x_{t}-\right.\right.$ $\left.\left.\sum_{k^{\prime }=1}^{K}A_{k^{\prime },h_{1}}^{\left(1\right)}x_{t-k^{\prime }}\right)dx_{T-1}\ldots dx_{T-k+1}\right\}$, which is equal to $\tilde{\zeta^{*}}\times \frac{{\left(u_{h_{\sigma },l}\right)}^{(D-1)k}}{\lambda_{D}^{Dk/2}\left(\Sigma_{h_{\sigma }}^{\left(2\right)}\right)}$ since the integral is one, where $\tilde{\zeta^{*}}$ is some constant.

Next, we need to derive an upper bound for $\zeta_{1:(T-k)}$. Note that $\zeta_{1:(T-k)}\leq \zeta^{*}\times$ $\frac{{\left(u_{h_{\sigma },l}\right)}^{\left(D-1)(T-k\right)}}{\lambda_{D}^{D(T-k)/2}\left(\Sigma_{\left.h_{\sigma }\right)}^{\left(2\right)}\right)}\int_{\left(T-k\right)}\left\{{\left\|x_{T-k}\right\|}^{2}\prod_{t=1}^{T-k}\phi_{\frac{1}{2}\lambda_{D}\left(\Sigma_{h_{\sigma }}^{\left(2\right)}\right)I_{D}}\left(x_{t}-\sum_{k^{\prime }=1}^{K}A_{k^{\prime },h_{1}}^{\left(1\right)}x_{t-k^{\prime }}\right)dx_{T-k}\right\}dx_{T-k-1}\ldots dx_{1}=$ $\zeta^{*}\times \frac{{\left(u_{h_{\sigma },l}\right)}^{\left(D-1)(T-k\right)}}{\lambda_{D}^{D(T-k)/2}\left(\Sigma_{h_{\sigma }}^{\left(2\right)}\right)}I_{1:(T-k)}$, where $\zeta^{*}$ is some constant and $I_{1:(T-k)}$ denotes the integral term. Therefore, ${𝒦}^{*}\leq \sum_{k=1}^{K}\left\|\mathrm{vec}\left(A_{k,h_{1}}^{\left(1\right)}-A_{k,h_{1}}^{\left(2\right)}\right)\right\|\times \sqrt{{\tilde{\zeta }}^{*}\times \frac{{\left(u_{h_{\sigma },l}\right)}^{(D-1)k}}{\lambda_{D}^{Dk/2}\left(\Sigma_{h_{\sigma }}^{\left(2\right)}\right)}\times \zeta^{*}\times \frac{{\left(u_{h_{\sigma },l}\right)}^{\left(D-1)(T-k\right)}}{\lambda_{D}^{D(T-k)/2}\left(\Sigma_{h_{\sigma }}^{\left(2\right)}\right)}I_{1:(T-k)}}=$ $\tilde{\zeta^{**}}\sum_{k=1}^{K}\left\|\mathrm{vec}\left(A_{k,h_{1}}^{\left(1\right)}-A_{k,h_{1}}^{\left(2\right)}\right)\right\|\times \frac{{\left(u_{h_{\sigma },l}\right)}^{(D-1)T/2}}{\lambda_{D}^{DT/2}\left(\Sigma_{h_{\sigma }}^{\left(2\right)}\right)}\times \sqrt{I_{1:(T-k)}}$. Recalling that $I_{1:(T-k)}=$ $\int \left\{{\left\|x_{T-1}\right\|}^{2}\prod_{t=1}^{T-1}\phi_{\lambda_{D}\left(\Sigma_{h\sigma }^{\left(2\right)}\right)I_{D}}\left(x_{t}-\sum_{k^{\prime }=1}^{K}A_{k^{\prime },h_{1}}^{\left(1\right)}x_{t-k^{\prime }}\right)dx_{T-1}\right\}dx_{T-2}\ldots dx_{1}$, we note that $I_{1:(T-k)}$ can be bounded using the relationship $E\left(\frac{{\left\|x_{T-k}\right\|}^{2}}{\frac{1}{2}\lambda_{D}\left(\Sigma_{h_{\sigma }}^{\left(2\right)}\right)}\right)=D+\sum_{l^{\prime }=1}^{D}{\left(\sum_{k^{\prime }=1}^{K}A_{k^{\prime },h_{1}}^{\left(1\right)}\left(l^{\prime },\cdot \right)x_{T-k-k^{\prime }}\right)}^{2}\leq D+K{\overset{¯}{a}}_{h_{1},j}^{2}\left(\sum_{k^{\prime }=1}^{K}D{\left\|x_{T-k-k^{\prime }}\right\|}^{2}\right)$. In other words, $I_{1:(T-k)}\leq \frac{D}{2}\lambda_{D}\left(\Sigma_{h_{\sigma }}^{\left(2\right)}\right)+K{\overset{‾}{a}}_{h_{1},j}^{2}\int \;\left(\sum_{k^{\prime }=1}^{K}D{\left\|x_{T-k-k^{\prime }}\right\|}^{2}\right)\prod_{t=1}^{T-k-1}\phi_{\frac{1}{2}\lambda_{D}\left(\Sigma_{h_{\sigma }}^{\left(2\right)}\right)I_{D}}\left(x_{t}-\sum_{k^{\prime }=1}^{K}A_{k^{\prime },h_{1}}^{\left(1\right)}x_{t-k^{\prime }}\right)dx_{T-k-1}\ldots dx_{1},=\frac{D}{2}\lambda_{D}\left(\Sigma_{h_{\sigma }}^{\left(2\right)}\right)+KD{\overset{‾}{a}}_{h_{1},j}^{2}I_{1:(T-k-1)}^{*}=\frac{D}{2}\lambda_{D}\left(\Sigma_{h_{\sigma }}^{\left(2\right)}\right)+KD{\overset{‾}{a}}_{h_{1},j}^{2}\left\{{\tilde{I}}_{1:(T-k-1),1}^{*}+\ldots +{\tilde{I}}_{1:(T-k-1),K}^{*}\right\},$ using Cauchy-Schwarz inequality and the sieve constructions, and where $I_{1:(T-k-1)}^{*}$ denotes the integral in the first line of the above upper bound, which is decomposable into K different integrals denoted as $\left\{{\tilde{I}}_{1:(T-k-1),1}^{*},\ldots ,{\tilde{I}}_{1:(T-k-1),K}^{*}\right\}$. For $k^{\prime }=1$, the term ${\tilde{I}}_{1:(T-k-1),1}^{*}$ in the above integral can be written as

$$=\int \|x_{T-k-1}{\|}^{2}\phi_{\frac{1}{2}\lambda_{D}\left({\text{Σ}}_{h_{\sigma }}^{\left(2\right)}\right)I_{D}}\left(x_{T-k-1}-\sum_{k^{\prime }=1}^{K}A_{k^{\prime },h_{1}}^{\left(1\right)}x_{t-k-1-k^{\prime }}\right)dx_{T-k-1}\;\times \int \left\{\prod_{t=1}^{T-k-2}\phi_{\frac{1}{2}\lambda_{D}\left({\text{Σ}}_{h_{\sigma }}^{\left(2\right)}\right)I_{D}}\left(x_{t}-\sum_{k^{\prime }=1}^{K}A_{k^{\prime },h_{1}}^{\left(1\right)}x_{t-k^{\prime }}\right)dx_{T-k-2}\ldots dx_{1}\right\}\;\leq \frac{D}{2}\lambda_{D}\left({\text{Σ}}_{h_{\sigma }}^{\left(2\right)}\right)+KD{\bar{a}}_{h_{1},j}^{2}I_{1:(T-k-2)}^{*}=\frac{D}{2}\lambda_{D}\left({\text{Σ}}_{h_{\sigma }}^{\left(2\right)}\right)+KD{\bar{a}}_{h_{1},j}^{2}\times \left\{{\tilde{I}}_{1:(T-k-2),1}^{*}+\ldots +{\tilde{I}}_{1:(T-k-2),K}^{*}\right\},$$

using similar notations as previously used. Similarly, when $k^{\prime }=2$, the term ${\tilde{I}}_{1:(T-k-1),2}^{*}$ in the upper bound for $I_{1:(T-k)}$ may be written as ${\tilde{I}}_{1:(T-k-1),2}^{*}\leq \frac{D}{2}\lambda_{D}\left(\Sigma_{h_{\sigma }}^{\left(2\right)}\right)+KD{\overset{‾}{a}}_{h_{1},j}^{2}I_{1:(T-k-3)}^{*}=$ $\frac{D}{2}\lambda_{D}\left(\Sigma_{h_{\sigma }}^{\left(2\right)}\right)+KD{\overset{‾}{a}}_{h_{1},j}^{2}\times \left\{{\tilde{I}}_{1:(T-k-3),1}^{*}+\ldots +{\tilde{I}}_{1:(T-k-3),K}^{*}\right\}$, using similar notation as above.

The number of terms in the above expression can be derived via a decision tree. For lag K, the total number of terms will be less than or equal to $K^{T-1}$. In the special case when K=1, the upper bound for $I_{T-1}$ is given as

(24)
$$I_{1:(T-1)}\leq D\frac{1}{2}\lambda_{D}\left(\Sigma_{h_{\sigma }}^{\left(2\right)}\right)\left\{1+KD{\overset{‾}{a}}_{h_{1},j}^{2}+{\left(KD{\overset{‾}{a}}_{h_{1},j}^{2}\right)}^{2}+\ldots +{\left(KD{\overset{‾}{a}}_{h_{1},j}^{2}\right)}^{T-2}\right\}+{\left(KD{\overset{‾}{a}}_{h_{1},j}^{2}\right)}^{T-1}\;\leq D\lambda_{D}\left(\Sigma_{h_{\sigma }}^{\left(2\right)}\right){\left(KD{\overset{‾}{a}}_{h_{1},j}^{2}\right)}^{T-2}+{\left(KD{\overset{‾}{a}}_{h_{1},j}^{2}\right)}^{T-1}\leq c^{*}{\left(KD{\overset{‾}{a}}_{h_{1},j}^{2}\right)}^{T-1},$$

where $c^{*}$ is some constant. Given that there are a total of $K^{T-1}$ terms, each being bounded as in (24), the total upper bound for lag K is given as

$$I_{1:(T-1)}\leq D\lambda_{D}\left(\Sigma_{h_{\sigma }}^{\left(2\right)}\right){\left(KD{\overset{‾}{a}}_{h_{1},j}^{2}\right)}^{T-2}+{\left(KD{\overset{‾}{a}}_{h_{1},j}^{2}\right)}^{T-1}\leq c^{*}{\left(KD{\overset{‾}{a}}_{h_{1},j}^{2}\right)}^{T-1}\times K^{T-1}=c^{*}{\left(K^{2}D{\overset{‾}{a}}_{h_{1},j}^{2}\right)}^{T-1}.$$

Hence using previous calculations, ${𝒦}^{*}\leq \frac{c^{**}}{{\underline{\sigma }}_{n}^{\left(DT+1\right)}}\times \left\{\sum_{k=1}^{K}\left\|\mathrm{vec}\left(A_{k,h_{1}}^{\left(1\right)}-A_{k,h_{1}}^{\left(2\right)}\right)\right\|{\left(u_{h_{\sigma },l}\right)}^{T(D-1)/2}\times \right.\left.{\left(DK^{2}{\overset{‾}{a}}_{h_{1},j}^{2}\right)}^{T-1}\right\}$. The second term in (23) is equal to $\|\phi_{\Sigma_{h_{\sigma }}^{\left(2\right)}}\left(x_{T}-\sum_{k=1}^{K}A_{k,h_{1}}^{\left(2\right)}x_{T-k}\right)\left\{\prod_{t=1}^{T-1}\phi_{\Sigma_{h_{\sigma }}^{\left(2\right)}}\left(x_{t}-\right.\right.\left.\left.\sum_{k=1}^{K}A_{k,h_{1}}^{\left(1\right)}x_{t-k}\right)-\prod_{t=1}^{T-1}\phi_{\Sigma_{h_{\sigma }}^{\left(2\right)}}\left(x_{t}-\sum_{k=1}^{K}A_{k,h_{2}}^{\left(2\right)}x_{t-k}\right)\right\}{\|}_{1}=\prod_{t=1}^{T-1}\phi_{\Sigma_{h_{\sigma }}^{\left(2\right)}}\left(x_{t}-\sum_{k=1}^{K}A_{k,h_{1}}^{\left(1\right)}x_{t-k}\right)-\prod_{t=1}^{T-1}\phi_{\Sigma_{h_{\sigma }}^{\left(2\right)}}\left(x_{t}-\sum_{k=1}^{K}A_{k,h_{1}}^{\left(2\right)}x_{t-k}\right){\|}_{1}$, since ${\left\|\phi_{\Sigma_{h_{\sigma }}^{\left(2\right)}}\left(x_{T}-\sum_{k=1}^{K}A_{k,h_{1}}^{\left(2\right)}x_{T-k}\right)\right\|}_{1}=1$. Hence the first term in the upper bound in (22) can be written as

(25)
$${𝒦}^{*}≲c^{**}\frac{\overset{k=1}{\underset{K}{\sum }}\|vec\left(A_{k,h_{1}}^{\left(1\right)}-A_{k,h_{1}}^{\left(2\right)}\right)\|{\left(u_{h_{\sigma },l}\right)}^{T(D-1)/2}}{{\underline{\sigma }}_{n}^{DT+1}}\times {\left(DK^{2}{\bar{a}}_{h_{1},j}^{2}\right)}^{T-1}\;+\|\overset{t=1}{\underset{T-1}{\prod }}\phi_{{\text{Σ}}_{h_{\sigma }}^{\left(2\right)}}\left(x_{t}-\overset{k=1}{\underset{K}{\sum }}A_{k,h_{1}}^{\left(1\right)}x_{t-k}\right)-\overset{t=1}{\underset{T-1}{\prod }}\phi_{{\text{Σ}}_{h_{\sigma }}^{\left(2\right)}}{\left(x_{t}-\overset{k=1}{\underset{K}{\sum }}A_{k,h_{1}}^{\left(2\right)}x_{t-k})\right\|}_{1}\;≲c^{**}T\overset{k=1}{\underset{K}{\sum }}\|vec\left(A_{k,h_{1}}^{\left(1\right)}-A_{k,h_{1}}^{\left(2\right)})\right\|{\left(u_{h_{\sigma },l}\right)}^{T(D-1)/2}\times {\left\{\frac{{\underline{\sigma }}_{n}^{TD+1}}{{\left(DK^{2}{\bar{a}}_{h_{1},j}^{2}\right)}^{T-1}}\right\}}^{-1}$$

using similar steps to obtain an upper bound for $\|\prod_{t=1}^{T-1}\phi_{\Sigma_{h_{\sigma }}^{\left(2\right)}}\left(x_{t}-\sum_{k=1}^{K}A_{k,h_{1}}^{\left(1\right)}x_{t-k}\right)-$ $\prod_{t=1}^{T-1}\phi_{\Sigma_{h_{\sigma }}^{\left(2\right)}}\left(x_{t}-\sum_{k=1}^{K}A_{k,h_{1}}^{\left(2\right)}x_{t-k}\right){\|}_{1}$. For the second term in (22), note that

(26)
$$\|\prod_{t=1}^{T}\phi_{\sum_{h^{\sigma }}^{\left(1\right)}}\left(x_{t}-\sum_{k=1}^{K}A_{k,h_{1}}^{\left(1\right)}X_{t}-k\right)-\prod_{t=1}^{T}\phi_{\sum_{h^{\sigma }}^{\left(2\right)}}\left(x_{t}-\sum_{k=1}^{K}A_{k,h_{1}}^{\left(1\right)}X_{t}-k\right){\|}_{1}\leq \;\|\prod_{t=1}^{T}\phi_{{\tilde{\sum }}_{h^{\sigma }}}\left(x_{t}-\sum_{k=1}^{K}A_{k,h_{1}}^{\left(1\right)}X_{t}-k\right)-\prod_{t=1}^{T}\phi_{\sum_{h^{\sigma }}^{\left(2\right)}}\left(x_{t}-\sum_{k=1}^{K}A_{k,h_{1}}^{\left(1\right)}X_{t}-k\right){\|}_{1}+\;\|\prod_{t=1}^{T}\phi_{{\tilde{\sum }}_{h^{\sigma }}}\left(x_{t}-\sum_{k=1}^{K}A_{k,h_{1}}^{\left(1\right)}X_{t}-k\right)-\prod_{t=1}^{T}\phi_{\sum_{h^{\sigma }}^{\left(2\right)}}\left(x_{t}-\sum_{k=1}^{K}A_{k,h_{1}}^{\left(1\right)}X_{t}-k\right){\|}_{1},$$

where ${\tilde{\Sigma }}_{h_{\sigma }}={\left(O_{t,h_{\sigma }}^{\left(2\right)}\Lambda_{t,h_{\sigma }}^{\left(1\right)}{\left(O_{th_{\sigma }}^{\left(2\right)}\right)}^{\prime }\right)}^{-1}$, and $\Sigma_{h_{\sigma }}^{\left(j\right)}={\left(O_{t,h_{\sigma }}^{\left(j\right)}\Lambda_{t,h_{\sigma }}^{\left(j\right)}{\left(O_{t,h_{\sigma }}^{\left(j\right)}\right)}^{\prime }\right)}^{-1},j=1,2$. Using Csiszar’s inequality the first term in (26) ≤

(27)
$$\sqrt{2\int \cdots \int \log\left(\prod_{t=1}^{T}\frac{(\phi_{{\tilde{\sum }}_{h_{\sigma }}-}\sum_{k=1}^{K}A_{k,h_{1}}^{\left(1\right)}x_{t}-k)}{(\phi_{\sum_{h_{\sigma }}^{\left(2\right)}-}\sum_{k=1}^{K}A_{k,h_{1}}^{\left(1\right)}x_{t}-k)}\right)}\left\{\prod_{t=1}^{T}(\phi_{{\tilde{\sum }}_{h_{\sigma }}(x_{t}-}\sum_{k=1}^{K}A_{k,h_{1}}^{\left(1\right)}x_{t}-k)\right\}dx_{T}\ldots dx_{1}\;=\sqrt{2\times \frac{1}{2}\sum_{t=1}^{T}\left\{\log\;\det(\sum_{h_{\sigma }}^{\left(-1\right)}\sum_{h_{\sigma }}^{\left(2\right)})+tr((\sum_{h_{\sigma }}^{\left(2\right)}{)}^{-1}{\tilde{\sum }}_{h_{\sigma }})-D\right\}}\;=\sqrt{\sum_{t=1}^{T}\sum_{d^{\prime }=1}^{D}\left\{\frac{\lambda d^{\prime }(\sum_{h_{\sigma }}^{\left(1\right)})}{\lambda d^{\prime }({\tilde{\sum }}_{h_{\sigma }}^{\left(2\right)})}-\log(\frac{\lambda d^{\prime }({\tilde{\sum }}_{h_{\sigma }}^{\left(1\right)})}{\lambda d^{\prime }({\tilde{\sum }}_{h_{\sigma }}^{\left(2\right)})})-1\right\}}.$$

Similarly, the second term in R.H.S. of $(26)\leq \sqrt{\sum_{t=1}^{T}\left\{logdet\left({\tilde{\Sigma }}_{h_{\sigma }}^{-1}\Sigma_{h_{\sigma }}^{\left(1\right)}\right)+\mathrm{tr}\left({\left(\Sigma_{h_{\sigma }}^{\left(1\right)}\right)}^{-1}{\tilde{\Sigma }}_{h_{\sigma }}\right)-D\right\}}$.

Using similar steps as in the proof of Lemma 2 in Canale and De Blasi (2017), it is possible to show that the second term in the R.H.S. of (26) (and hence the last term in the R.H.S. of (22)), has an upper bound given by $\sqrt{2T{\left\|O_{h_{\sigma }}^{\left(1\right)}-O_{h_{\sigma }}^{\left(2\right)}\right\|}_{2}\frac{\lambda_{1}\left(\Sigma_{h_{\sigma }}^{\left(1\right)}\right)}{\lambda_{D}\left(\Sigma_{h_{\sigma }}^{\left(1\right)}\right)}}$. Finally, we need to establish an upper bound for the term $\sum_{h_{1},h_{\sigma }<H_{n}}\left|{\tilde{\pi }}_{h_{1}}^{\left(1\right)}{\tilde{\pi }}_{\sigma ,h_{\sigma }}^{\left(1\right)}-\pi_{h_{1}}^{\left(2\right)}\pi_{\sigma ,h_{\sigma }}^{\left(2\right)}\right|$ in (21), which is given by Lemma 5 as $\sum_{h_{1},h_{\sigma }<H_{n}}\left|{\tilde{\pi }}_{h_{1}}^{\left(1\right)}{\tilde{\pi }}_{\sigma ,h_{\sigma }}^{\left(1\right)}-\pi_{h_{1}}^{\left(2\right)}\pi_{\sigma ,h_{\sigma }}^{\left(2\right)}\right|+\left|1-(1-\epsilon {)}^{2}\right|$, where $\tilde{\pi }=\frac{\pi_{h}}{\left(1-\sum_{h>H}\pi_{h}\right)}$.

For a given $f_{P}\in {ℱ}_{n,\mathrm{jl}}$ with $P=\sum_{h_{1}\geq 1}\sum_{h_{\sigma }\geq 1}\pi_{h_{1}}^{\left(1\right)}\pi_{\sigma ,h_{\sigma }}^{\left(1\right)}\delta_{\left(\Theta_{h_{1}}^{\left(1\right)},\Sigma_{h_{\sigma }}^{\left(1\right)}\right)}$ and $\Sigma_{h}={\left(O_{h}\Lambda_{h}O_{h}^{T}\right)}^{-1}$ where $\Lambda_{h}=\mathrm{diag}\left(\lambda_{h,1},\ldots ,\lambda_{h,D}\right)$, we will construct another density $f_{\overset{ˆ}{P}}$ with $\overset{ˆ}{P}=\sum_{h_{1}\geq 1}\sum_{h_{\sigma }\geq 1}\pi_{h_{1}}^{\left(1\right)}\pi_{\sigma ,h_{\sigma }}^{\left(1\right)}\delta \left({\overset{ˆ}{\Theta }}_{h_{1}}^{\left(1\right)}{\overset{ˆ}{\Sigma }}_{h_{\sigma }}^{\left(1\right)}\right)$ within the ϵ-net and then compute the cardinality of the ϵ-net set to derive an upper bound for the entropy of sieve ${ℱ}_{n,jl}$. To construct such a density, we will choose:

**1.** ${\overset{ˆ}{A}}_{k,h_{1}}\in {\overset{ˆ}{ℛ}}_{h_{1}},h_{1}=1,\ldots ,H$, where ${\overset{ˆ}{ℛ}}_{h_{1}}$ is a $\epsilon^{*}$-net of ${ℛ}_{h_{1}}:=\left\{A\in {ℜ}^{D\times D}:{\underline{a}}_{h_{1},j}\leq \right.$ $\left.\|\mathrm{vec}(A)\|\leq {\overset{‾}{a}}_{h_{1},j}\right\}$, such that $\left\|\mathrm{vec}(A{)}_{k,h_{1}}-\mathrm{vec}(\overset{ˆ}{A}{)}_{k,h_{1}}\right\|\leq \epsilon^{*},k=1,\ldots ,K$, where $\epsilon^{*}=\pi \epsilon \left\{\frac{{\underline{\sigma }}_{n}^{TD+1}}{2T{\left(u_{h_{\sigma },l}\right)}^{T(D-1)/2}\times {\left(DK^{2}{\overset{‾}{a}}_{h_{1},j}^{2}\right)}^{T-1}}\right\}$ using (25) and the fact that ${\underline{\sigma }}_{n}<1$ for large n.

**2.** $\left\{{\overset{ˆ}{\pi }}_{h_{1}}{\overset{ˆ}{\pi }}_{h_{\sigma }},h_{1},h_{\sigma }\leq H_{n}\right\}\in \overset{ˆ}{\Delta }$, where $\overset{ˆ}{\Delta }$ is a ϵ-net of a $H_{n}^{2}$ dimensional probability simplex such that $\sum_{h_{1},h_{\sigma }\leq H_{n}}\left|{\tilde{\pi }}_{h_{1}}{\tilde{\pi }}_{h_{\sigma }}-{\overset{ˆ}{\pi }}_{h_{1}}{\overset{ˆ}{\pi }}_{h_{\sigma }}\right|\leq \epsilon$, and ${\tilde{\pi }}_{h}=\frac{\pi_{h}}{\sum_{h\leq H_{n}}\pi_{h}},h\leq H_{n}$.

**3.** ${\overset{ˆ}{O}}_{h}\in {\overset{ˆ}{𝒪}}_{h}$, where ${\overset{ˆ}{𝒪}}_{h}$ is a $\delta_{h}$-net of the set ${𝒪}_{h}$ defined as the set of D×D orthogonal matrices with respect to the spectral norm $\|\cdot {\|}_{2}$ with $\delta_{h}=\epsilon^{2}/\left(2Du_{h,l}\right)$ such that $\|O_{h}-$ ${\overset{ˆ}{O}}_{h}{\|}_{2}\leq T\delta_{h}$.

**4.** $\left(m_{h,1},\ldots ,m_{h_{D}}\right)\in \{1,\ldots ,M{\}}^{D},h=1,\ldots ,H$, such that ${\overset{ˆ}{\lambda }}_{h,l}={\left\{{\underline{\sigma }}^{2}(1+\epsilon \sqrt{D}{)}^{m_{h,l}-1}\right\}}^{-1}$ will satisfy $1\leq {\overset{ˆ}{\lambda }}_{h,l}/\lambda_{h,l}<(1+\epsilon /\sqrt{D})$.

Using this construction, the term (27) is shown to be bounded by $\sqrt{T\sum_{d^{\prime }=1}^{D}\left\{{\left(\frac{{\overset{ˆ}{\lambda }}_{h,D-d^{\prime }+1}}{\lambda_{h,D-d^{\prime }+1}}-1\right)}^{2}\right\}}$. Moreover under this construction, it can be shown that ${\left\|f_{P}-f_{\overset{ˆ}{P}}\right\|}_{1}<C^{*}\epsilon$ for some constant $C^{*}$, by employing some additional algebra and the above arguments. Further, the cardinality of the ϵ-net can be computed by noting that $\#(\overset{ˆ}{\Delta })≲\epsilon^{-H_{n}^{2}}$ for $j=1,2,\#\left({\overset{ˆ}{𝒪}}_{h}\right)≲$ $\delta_{h}^{-D(D-1)/2},\#\left({\overset{ˆ}{ℛ}}_{k,h}\right)≲\left[{\left(\frac{{\overset{‾}{a}}_{h}}{\epsilon^{*}}+1\right)}^{D^{2}}-{\left(\frac{{\underline{a}}_{h}}{\epsilon^{*}}-1\right)}^{D^{2}}\right]$. Using these quantities, one can write the upper bound for the exponential of the entropy bound as $≲(M{)}^{DH_{n}}\epsilon^{-H_{n}^{2}}\times {𝒦}^{*}$, where

$${𝒦}^{*}=\underset{h_{1}\leq H_{n}}{\prod }{\left\{{\left(\frac{{\bar{a}}_{h_{1},j}}{\epsilon^{*}}+1\right)}^{D^{2}}-{\left(\frac{{\underline{a}}_{h_{1},j}}{\epsilon^{*}}-1\right)}^{D^{2}}\right\}}^{K}\underset{h_{\sigma }\leq H_{n}}{\prod }{\left\{\frac{2Du_{h_{\sigma },l}}{\epsilon^{2}}\right\}}^{D(D-1)/2}\;≲\underset{h_{\sigma }\leq H_{n}}{\prod }{\left\{\frac{2Du_{h_{\sigma },l}}{\epsilon^{2}}\right\}}^{D(D-1)/2}\underset{h_{1}\leq H_{n}}{\prod }{\left\{{\left(\frac{C_{h_{1,j},h_{\sigma ,l}}^{*}{\bar{a}}_{h_{1},j}}{{\underline{\sigma }}_{n}\epsilon }+1\right)}^{D^{2}}-{\left(\frac{C_{h_{1,j},h_{\sigma ,l}l}^{*}h_{h_{1},j}}{\sigma_{n}\epsilon }-1\right)}^{D^{2}}\right\}}^{K}$$

and $C_{h_{1,j},h_{\sigma ,l}}^{*}=\frac{2}{\pi }\left\{T{\left(u_{h_{\sigma },l}\right)}^{(T)(D-1)/2}\right\}\times \frac{{\left(DK^{2}{\overset{‾}{a}}_{h_{1},j}^{2}\right)}^{T-1}}{{\underline{\sigma }}_{n}^{TD}}$.

**Proof of Corollary 1:** For computing the sieve entropy bound corresponding to lgPDPM-VAR, note that using similar calculations as in (21), one can show that $|f_{P_{1}}-f_{P_{2}}{\|}_{1}=$

(28)
$$\sum_{h_{11}}\ldots \sum_{h_{1K}}\sum_{h_{\sigma }<H_{n}}\left(\prod_{k=1}^{K}\pi_{k,h_{1k}}^{\left(1\right)}\right)\pi_{\sigma ,h_{\sigma }}^{\left(1\right)}\|\prod_{t=1}^{T}\phi_{{\text{Σ}}_{h_{\sigma }}^{\left(1\right)}}\left(x_{t}-\sum_{k=1}^{K}A_{k,h_{1k}}^{\left(1\right)}x_{t-k}\right)-\prod_{t=1}^{T}\phi_{{\text{Σ}}_{h_{\sigma }}^{\left(2\right)}}\left(x_{t}-\sum_{k=1}^{K}A_{k,h_{1k}}^{\left(2\right)}x_{t-k}\right){\|}_{1}\;+\sum_{h_{11}}\cdots \sum_{h_{1K}}\sum_{h_{\sigma }<H_{n}}\left|\left(\prod_{k=1}^{K}\pi_{k,h_{1k}}^{\left(1\right)}\right)\pi_{\sigma ,h_{\sigma }}^{\left(1\right)}-\left(\prod_{k=1}^{K}\pi_{k,h_{1k}}^{\left(2\right)}\right)\pi_{\sigma ,h_{\sigma }}^{\left(2\right)}\right|+K\epsilon_{1}^{K}+L\epsilon_{1},$$

for some constant L. Using similar calculations as under the PDPM-VAR case, $N\left(\epsilon_{1},{ℱ}_{n,jl},\|\right.$. $\left.{\|}_{1}\right)≲{\left(\frac{M^{D}}{\epsilon_{1}^{K}}\right)}^{H_{n}}\prod_{k=1}^{K}\prod_{h_{1k}\leq H_{n}}\left\{{\left(\frac{{\overset{‾}{a}}_{h_{1k},j}}{\epsilon_{1}^{k}}+1\right)}^{D^{2}}-{\left(\frac{{\underline{a}}_{h_{1k},j}}{\epsilon_{1}}-1\right)}^{D^{2}}\right\}\prod_{h_{\sigma }\leq H_{n}}{\left\{\frac{2Du_{h_{\sigma },l}}{\epsilon_{1}^{2}}\right\}}^{D(D-1)/2}≲$ ${\left(\frac{M^{D}}{\epsilon_{1}^{C_{1}^{*}}}\right)}^{H_{n}}\times \prod_{h_{\sigma }\leq H_{n}}{\left\{\frac{2Du_{h_{\sigma ,l}}}{\epsilon_{1}^{2}}\right\}}^{D(D-1)/2}\prod_{k=1}^{K}\prod_{h_{1k}\leq H_{n}}\left\{{\left(\frac{C_{h_{1,j},h_{\sigma ,l}}^{\sigma_{n}\epsilon_{1}}{\overset{‾}{a}}_{h_{1k},j}}{\sigma_{n}}+1\right)}^{D^{2}}-\left(\frac{C_{h_{1,j},h_{\sigma ,l}}^{**}{\underline{l}}_{h_{1k},j}}{{\underline{\sigma }}_{n}\epsilon_{1}}-\right.\right.$ $\left.1{)}^{D^{2}}\right\}$, where $C_{h_{1,j},h_{\sigma ,l}}^{**}=\frac{2}{\pi }\left\{\frac{T{\left(u_{h_{\sigma ,l}}\right)}^{(T-1)(D-1)/2}}{{\underline{\sigma }}_{n}^{TD}}\right\}\times {\left(DK^{2}max\left\{{\overset{‾}{a}}_{h_{11},j}^{2},\ldots ,{\overset{‾}{a}}_{h_{1K},j}^{2}\right\}\right)}^{T-1}$.

Similarly, for computing the entropy bound corresponding to sieves in the rgPDPM-VAR model, note that using similar calculations as before, one can show that the densities satisfy ${\left\|f_{P_{1}}-f_{P_{2}}\right\|}_{1}=\sum_{h_{11}\leq H_{n}}\cdots \sum_{h_{1D}\leq H_{n}}\sum_{h_{\sigma }<H_{n}}\left|\left(\prod_{d^{\prime }=1}^{D}\pi_{d^{\prime },h_{1d^{\prime }}}^{*(1)}\right)\pi_{\sigma ,h_{\sigma }}^{\left(1\right)}-\left(\prod_{d^{\prime }=1}^{D}\pi_{d^{\prime },h_{1d^{\prime }}}^{*(2)}\right)\pi_{\sigma ,h_{\sigma }}^{\left(2\right)}\right|+$ $\sum_{h_{11}\leq H_{n}}\ldots \sum_{h_{1D}\leq H_{n}}\sum_{h_{\sigma }\leq H_{n}}\left(\prod_{d^{\prime }=1}^{D}\pi_{d^{\prime },h_{1d^{\prime }}}^{*(1)}\right)\pi_{\sigma ,h_{\sigma }}^{\left(1\right)}\times \|\prod_{t=1}^{T}\phi_{\Sigma_{h_{\sigma }}^{\left(1\right)}}\left(x_{t}-\sum_{k=1}^{K}A_{k,h_{11},\ldots ,h_{1d}}^{\left(1\right)}x_{t-k}\right)-$ $\prod_{t=1}^{T}\phi_{\Sigma_{h_{\sigma }}^{\left(2\right)}}\left(x_{t}-\sum_{k=1}^{K}A_{k,h_{11},\ldots ,h_{1d}}^{\left(2\right)}x_{t-k}\right){\|}_{1}+K\epsilon_{2}^{K}+L_{2}^{*}\epsilon_{2}$, for some constant $L_{2}^{*}$. Similar calculations as before yield the bound for exponential of the entropy $\left.N\left(\epsilon_{2},{ℱ}_{n,\mathrm{jl}},\|\cdot {\|}_{1}\right)\right)$ as:

(29)
$$≲{\left(\frac{M^{D}}{\epsilon_{2}^{D}}\right)}^{H_{n}}\underset{h_{\sigma }\leq H_{n}}{\prod }{\left\{\frac{2Du_{h_{\sigma },l}}{\epsilon_{2}^{2}}\right\}}^{D(D-1)/2}\overset{D}{\underset{d^{\prime }=1}{\prod }}\underset{h_{1d^{\prime }}\leq H_{n}}{\prod }{\left\{{\left(\frac{{\bar{a}}_{h_{1d^{\prime }},j}}{\epsilon_{2}^{*}}+1\right)}^{D}-{\left(\frac{{\underline{a}}_{h_{11^{\prime }},j}}{\epsilon_{2}^{*}}-1\right)}^{D}\right\}}^{K}≲{\left(\frac{M^{D}}{\epsilon_{2}^{C_{1}^{*}}}\right)}^{H_{n}}\;\times \underset{h_{\sigma }\leq H_{n}}{\prod }{\left\{\frac{2Du_{h_{\sigma },l}}{\epsilon_{2}^{2}}\right\}}^{D(D-1)/2}\overset{D}{\underset{d^{\prime }=1}{\prod }}\underset{h_{1d^{\prime }}\leq H_{n}}{\prod }{\left\{{\left(\frac{{\tilde{C}}_{h_{1,j},h_{\sigma ,l}}^{*}{\underline{a}}_{h_{1d^{\prime }},j}}{{\underline{\sigma }}_{n}\epsilon_{2}}+1\right)}^{D}-{\left(\frac{{\tilde{C}}_{h_{1,j},h_{\sigma ,l}}^{*}{\underline{a}}_{h_{1d^{\prime }},j}}{{\underline{\sigma }}_{n}\epsilon_{2}}-1\right)}^{D}\right\}}^{K}$$

where ${\tilde{C}}_{h_{1,j},h_{\sigma ,l}}^{*}=\frac{2}{\pi }\left\{\frac{T(u_{h_{\sigma ,l}}^{(T-1)(D-1)/2}}{{\underline{\sigma }}_{n}^{TD}}\right\}\times {\left(DK^{2}\max\left\{{\bar{a}}_{h_{11},j}^{2},\ldots ,{\bar{a}}_{h_{1d},j}^{2}\right\}\right)}^{T-1}$.

**Proof of Theorem 5:** The proof follows using Theorem 2 and the entropy bounds established in Theorem 3. For the case of PDPM-VAR, consider the sieves

(30)
$${ℱ}_{n,j,l}=\{f_{p}\in {ℱ}_{n}:\text{for}\;h_{1},h_{\sigma }\leq H_{n},n^{H_{n}^{2}}\left(j_{h_{1}}-1\right)\leq \|vec\left(A_{k,h_{1}})\right\|\leq n^{H_{n}^{2}}j_{h_{1}},\forall k,\;n^{l_{h_{\sigma }}-1}<\frac{\lambda_{1}\left({\text{Σ}}_{h_{\sigma }}\right)}{\lambda_{D}\left({\text{Σ}}_{h_{\sigma }}\right)}\leq n^{l_{h_{\sigma }}}\},{ℱ}_{n}=\{f_{p}:P=\underset{h_{1}\geq 1}{\sum }\underset{h_{\sigma }\geq 1}{\sum }\pi_{h_{1}}\pi_{\sigma ,h_{\sigma }}\delta_{{\text{Θ}}_{h_{1}},{\text{Σ}}_{h_{\sigma }}}:\underset{h_{1}>H_{n}}{\sum }\pi_{h_{1}}<\epsilon ,\;\underset{h_{\sigma }>H_{n}}{\sum }\pi_{\sigma ,h_{\sigma }}<\epsilon ,\text{for}\;h_{\sigma }\leq H_{n},{\underline{\sigma }}_{n}^{2}\leq \lambda_{D},\lambda_{1}\leq {\underline{\sigma }}_{n}^{2}{\left(1+\epsilon /\sqrt{D}\right)}^{M_{n}},1<\frac{\lambda_{1}}{\lambda_{D}}\leq n^{H_{n}}\},$$

where $M_{n}={\underline{\sigma }}^{-2c_{2}}=n$ and $H_{n}=\left\lfloor Cn\epsilon^{2}/log(n)\right\rfloor$ for some positive constant C, and clearly ${ℱ}_{n}\subset \cup_{j,1}{ℱ}_{n,j,1}$. Comparing to the notations used in the manuscript, we note that ${\underline{a}}_{h_{1},j}=n^{H_{n}}\left(j_{h_{1}}-1\right),{\overset{‾}{a}}_{h_{1},j}=n^{H_{n}}j_{h_{1}}$, and $u_{h_{\sigma },l}=n^{l_{h_{\sigma }}}$, for integers $\left(j_{1},\ldots ,j_{H_{n}}\right)\in \mathbb{N}$ and $\left(l_{1},\ldots ,l_{H_{n}}\right)\in \left\{1,\ldots ,H_{n}\right\}$.

Using Lemma 6 in the Appendix, it is clear that condition (2*A*) holds for the PDPM-VAR. Next, we will derive the entropy bounds and the complement of the prior probability for the sieves and illustrate that the summability condition *(2B)* in Theorem 2 holds.

Now, using Theorem 4, the upper bound on the entropy term is $≲{\left(M^{D}\epsilon^{-C_{1}}\right)}^{H_{n}}\times \;\overset{H_{n}}{\underset{h_{\sigma }=1}{\prod }}{\left\{\frac{2Du_{h_{\sigma },l}}{\epsilon^{2}}\right\}}^{D(D-1)/2}\times {𝒦}^{*},{𝒦}^{*}=\overset{H_{n}}{\underset{h_{1}=1}{\prod }}{\left\{{\left(\frac{C_{h_{1,j},h_{\sigma ,l}}^{*}{\bar{a}}_{h_{1},j}}{{\underline{\sigma }}_{n}\epsilon }+1\right)}^{D^{2}}-{\left(\frac{C_{h_{1,j},h_{\sigma ,l}}^{*}{\underline{\underline{a}}}_{h_{1},j}}{{\underline{\sigma }}_{n}\epsilon }-1\right)}^{D^{2}}\right\}}^{K},$

(31)
$$\text{i.e.}\;{𝒦}^{*}\approx \underset{h_{1}\leq H_{n}}{\prod }{\left(C_{h_{1,j},h_{\sigma ,l}}^{*}\right)}^{KD^{2}}{\left\{{\left(\frac{{\bar{a}}_{h_{1},j}}{{\underline{\sigma }}_{n}\epsilon }+o_{n}(1)\right)}^{D^{2}}-{\left(\frac{{\underline{a}}_{h_{1},j}}{{\underline{\sigma }}_{n}\epsilon }-o_{n}(1)\right)}^{D^{2}}\right\}}^{K}\;≲\underset{h_{1}\leq H_{n}}{\prod }{\left(C_{h_{1,j},h_{\sigma ,l}}^{*}\right)}^{KD^{2}}{\left\{{\left(\frac{{\bar{a}}_{h_{1,j}}}{{\underline{\sigma }}_{n}\epsilon }+1\right)}^{D^{2}}-{\left(\frac{{\underline{a}}_{h_{1},j}}{{\underline{\sigma }}_{n}\epsilon }-1\right)}^{D^{2}}\right\}}^{K}≲{\left\{{\left(u_{h_{\sigma },l}\right)}^{(T-1)(D-1)/2}\right\}}^{KD^{2}H_{n}}\;\times \underset{h_{1}\leq H_{n}}{\prod }{\left[\frac{2}{\pi }\left(T{\left(u_{h_{\sigma },l}\right)}^{(T)(D-1)/2}\right)\times \frac{{\left(DK^{2}{\bar{a}}_{h_{1},j}^{2}\right)}^{T-1}}{{\underline{\sigma }}_{n}^{TD}}\right]}^{KD^{2}}{\left\{{\left(\frac{{\bar{a}}_{h_{1},j}}{{\underline{\sigma }}_{n}\epsilon }+1\right)}^{D^{2}}-{\left(\frac{{\underline{a}}_{h_{1},j}}{{\underline{\sigma }}_{n}\epsilon }-1\right)}^{D^{2}}\right\}}^{K},$$

where $o_{n}(1)$ is a vanishing term with increasing sample size. Using similar steps as in the proof of Theorem 2 in Canale and De Blasi (2017), it is possible to show that $[(\frac{{\overset{‾}{a}}_{h_{1},j}}{{\underline{\sigma }}_{n}\epsilon /2}+1{)}^{D^{2}}-(\frac{{\underline{a}}_{h_{1},j}}{{\underline{\sigma }}_{n}\epsilon /2}-1{)}^{D^{2}}{]}^{K}≲{\left[\frac{n^{\left(H_{n}+\frac{1}{2c_{2}}\right)D^{2}}j_{h^{2}-1}^{D_{1}}}{(\epsilon {)}^{D^{2}}}\right]}^{K}$, when n and $j_{h_{1}}$ are large. Hence, when n is large enough,

$${\left[\frac{2T}{\pi }\frac{{\left(DK^{2}{\bar{a}}_{h_{1},j}^{2}\right)}^{T-1}}{{\underline{\sigma }}_{n}^{TD}}\right]}^{KD^{2}}{\left\{{\left(\frac{{\bar{a}}_{h_{1},j}}{{\underline{\sigma }}_{n}\epsilon }+1\right)}^{D^{2}}-{\left(\frac{{\underline{a}}_{h_{1},j}}{{\underline{\sigma }}_{n}\epsilon }-1\right)}^{D^{2}}\right\}}^{K}\;\leq {\left\{\frac{n^{\left(H_{n}^{2}+\frac{1}{2c_{2}}\right)D^{2}j_{h1}^{D^{2}-1}}}{{\left(\epsilon \right)}^{D^{2}}}\right\}}^{K}\times {\left[\frac{2T}{\pi }{\left(\frac{DK^{2}{\bar{a}}_{h_{1},j}^{2}}{{\underline{\sigma }}_{n}}\right)}^{T-1}\times {\left({\underline{\sigma }}_{n}\right)}^{-TD}\right]}^{KD^{2}}\;\approx 𝒞{\left\{\frac{n^{\left(H_{n}^{2}+\frac{1}{2c_{2}}\right)D^{2}}j_{h1}^{D^{2}-1}}{{\left(\epsilon \right)}^{D^{2}}}\right\}}^{K}\times {\left(n^{2H_{n}^{2}+1/\left(2c_{2}\right)}j_{h_{1}}^{2}\right)}^{KD^{2}(T-1)}\times {\left(n^{TD/c_{2}}\right)}^{KD^{2}}\leq 𝒞\times {\left(\frac{1}{\epsilon }\right)}^{KD^{2}}\times \;\exp\left\{H_{n}^{2}KD^{2}(2T-3)\log(n)+\frac{1}{c_{2}}\left(KD^{2}(T-1)+TKD^{3}\right)\log(n)\right\}\times {\left(j_{h1}\right)}^{2KD^{2}(T-2)+K\left(D^{2}-1\right)},$$

where 𝒞 is a constant independent of n. Further,

$${\left\{\frac{2Du_{h_{\sigma },l}}{\epsilon^{2}}\right\}}^{D(D-1)/2}{\left\{{\left(u_{h_{\sigma },l}\right)}^{(T)(D-1)/2}\right\}}^{KD^{2}H_{n}}={𝒞}_{1}{\left(u_{h_{\sigma },l}\right)}^{D(D-1)/2+KD^{2}H_{n}(T)(D-1)/2}\;\approx {𝒞}_{1}\text{exp}\left\{\left(\frac{D(D-1)}{2}+KD^{2}H_{n}(T)\frac{D-1}{2}\right)\text{log}\left(n^{l_{h_{\sigma }}}\right)\right\}\times {\left(\frac{1}{\epsilon }\right)}^{D(D-1)},$$

for large n, where the constant ${𝒞}_{1}$ that does not depend on n.

Hence we have

(32)
$$N\left(\epsilon ,{ℱ}_{n,jl},\|\cdot {\|}_{1}\right)≲{𝒞}_{1}^{H_{n}}\text{exp}\left\{DH_{n}\text{log}(M)+H_{n}\text{log}\frac{C_{1}}{\epsilon }\right\}\;\times \text{exp}\left\{H_{n}^{3}\left(2KD^{2}(T-2)+KD^{2}\right)\text{log}(n)+\frac{H_{n}}{c_{2}}\left(KD^{2}(T-1)+TKD^{3}\right)\text{log}(n)\right\}\;\times \left\{\prod_{h_{\sigma }\leq H_{n}}{\left(n^{l_{h_{\sigma }}}\right)}^{\frac{D(D-1)}{2}+KD^{2}H_{n}(T)\frac{D-1}{2}}\right\}\left\{\prod_{h_{1}\leq H_{n}}{\left(j_{h1}\right)}^{2KD^{2}(T-2)+K\left(D^{2}-1\right)}\right\}\times {\left(\frac{1}{\epsilon }\right)}^{H_{n}\left(KD^{2}+D(D-1)\right)}$$

Further, under the specification $P_{1}^{*}\left(A_{1},\ldots ,A_{K}\right)=\prod_{k=1}^{K}P_{1}^{*}\left(A_{k}\right)$, we have

(33)
$$\text{Π}\left({ℱ}_{n,jl}\right)\leq \prod_{h_{1}\leq H_{n}}P_{1}^{\text{*}}\left(\|\mathrm{vec}\left(A_{k})\right\|>n^{H_{n}^{2}}\left(j_{h_{1}}-1\right),\forall k\right)\prod_{h_{\sigma }\leq H_{n}}P_{2}^{\text{*}}\left(\lambda_{1}(\text{Σ})/\lambda_{D}(\text{Σ})>n^{\left(l_{h_{\sigma }}-1\right)}\right),\;≲\prod_{h_{1}\leq H_{n}}{\left\{{\left(n^{H_{n}^{2}}\left(j_{h1}-1\right)\right)}^{-1{\left(j_{h_{1}}\geq 2\right)}^{2K(r+1)}}\right\}}^{K}\times \prod_{h_{\sigma }\leq H_{n}}{\left(n^{\left(l_{h_{\sigma }}-1\right)}\right)}^{-1{\left(l_{h_{\sigma }}\geq 1\right)}^{\kappa }}\;\approx \left\{n^{-2H_{n}^{3}(r+1)K}\prod_{h_{1}\leq H_{n}}{\left(j_{h_{1}}-1\right)}^{-1{\left(j_{h_{1}}\geq 2\right)}^{2K(r+1)}}\right\}\times \left\{\prod_{h_{\sigma }\leq H_{n}}{\left(n^{\kappa \left(l_{h_{\sigma }}-1\right)}\right)}^{-1\left(l_{h_{\sigma }}\geq 1\right)}\right\}\text{, for large}\;n.$$

Under Lemma 7 in the Appendix, we can show that for the PDPM-VAR, $\sum_{j_{h_{1}}\in N}\sum_{1\leq l_{h_{\sigma }\leq H_{n}}}\sqrt{N\left(\epsilon ,{ℱ}_{n,j_{h_{1}}l_{h_{\sigma }}}\right)\Pi \left({ℱ}_{n,j_{h_{1}}l_{h_{\sigma }}}\right)}e^{-(4-c)n\epsilon^{2}}\to 0$ as n→∞ for a suitable choice of constants. Hence the condition (2*B*) in Theorem 2 is satisfied and the strong posterior consistency result is proved corresponding to the PDPM-VAR model.

To prove the summability result for the lgPDPM-VAR approach, consider the sieves defined in the manuscript as

(34)
$${ℱ}_{n}=\{f_{p}:P=\sum_{h_{11}=1}^{\infty }\ldots \sum_{h_{1K}=1}^{\infty }\sum_{h_{\sigma }=1}^{\infty }\pi_{\sigma ,h_{\sigma }}\left(\prod_{k=1}^{K}\pi_{k,h_{1k}}\right)\delta_{{\text{Θ}}_{h_{1k}},{\text{Σ}}_{h_{\sigma }}}:\sum_{h_{1,1k}>H_{n}}\pi_{h_{1,1k}}<\epsilon_{1},1\leq k\leq K,\;\sum_{h_{\sigma }>H_{n}}\pi_{\sigma ,h_{\sigma }}<\epsilon_{2},{\underline{\sigma }}_{n}^{2}\leq \lambda_{D}\left({\text{Σ}}_{h_{\sigma }}\right),\lambda_{1}\left({\text{Σ}}_{h_{\sigma }}\right)\leq {\underline{\sigma }}_{n}^{2}{\left(1+\epsilon /\sqrt{D}\right)}^{M_{n}},1<\frac{\lambda_{1}\left({\text{Σ}}_{h_{\sigma }}\right)}{\lambda_{D}\left({\text{Σ}}_{h_{\sigma }}\right)}\leq n^{H_{n}},h_{\sigma }\leq H_{n}\},\;{ℱ}_{n,j,l}=\{f_{p}\in {ℱ}_{n}:\text{for}\;h_{11},\ldots ,h_{1K}\leq H_{n},n^{H_{n}^{2}}\left(j_{h_{1k}}-1\right)\leq \mathrm{vec}\left(A_{kh_{1k}}\right)\leq n^{H_{n}^{2}}j_{h_{1k}},\;\text{and for}\;h_{\sigma }\leq H_{n},n^{l_{h_{\sigma }}-1}\leq \frac{\lambda_{1}\left({\text{Σ}}_{h_{\sigma }}\right)}{\lambda_{D}\left({\text{Σ}}_{h_{\sigma }}\right)}\leq n^{l_{\sigma }}\}$$

Now note that the prior probability on the sieve ${ℱ}_{n}$ satisfies

$$\text{Π}\left({ℱ}_{n}^{c}\right)\leq \mathrm{Pr}\left(\sum_{h_{\sigma }>H_{n}}\pi_{\sigma ,h_{\sigma }}>\epsilon_{1}\right)+\sum_{k=1}^{K}\mathrm{Pr}\left(\sum_{h_{1k}>H_{n}}\pi_{k,h_{1k}}>\epsilon_{1}\right)+H_{n}P_{2}^{\text{*}}\left(\lambda_{D}(\text{Σ})\leq {\underline{\sigma }}_{n}^{2}\right)+\;H_{n}P_{2}^{\text{*}}\left(\lambda_{1}(\text{Σ})>{\underline{\sigma }}_{n}^{2}{\left(1+\epsilon_{1}/\sqrt{D}\right)}^{M_{n}}\right)+H_{n}P_{2}^{\text{*}}\left(\frac{\lambda_{1}\left({\text{Σ}}_{h_{\sigma }}\right)}{\lambda_{D}\left({\text{Σ}}_{h_{\sigma }}\right)}>n^{H_{n}}\right)≲e^{-b^{\text{*}}n},$$

using similar techniques used to derive (37) in Lemma 6 of the Appendix. Hence, condition (2*A*) in Theorem 2 holds. Further, one can rewrite (33) as

$$\Pi \left({ℱ}_{n,jl}\right)≲\left\{n^{-2H_{n}^{3}(r+1)K}\prod_{k=1}^{K}\prod_{h_{1k}\leq H_{n}}{\left(j_{h_{1k}}-1\right)}^{-1{\left(j_{h_{1k}}\geq 2\right)}^{2(r+1)}}\right\}\times \left\{\prod_{h_{\sigma }\leq H_{n}}{\left(n^{\kappa \left(l_{h_{\sigma }}-1\right)}\right)}^{-1\left(l_{h_{\sigma }}\geq 1\right)}\right\},$$

for large n. Moreover, using similar steps as in the proof for Lemma 7 corresponding to the PDPM-VAR model, it is possible to show that the entropy bound in Corollary 1 satisfies $\sqrt{N\left(\epsilon_{1},{ℱ}_{n,j_{h_{1}}l_{h_{\sigma }}},\|\cdot {\|}_{1}\right)\Pi \left({ℱ}_{n,j_{h_{1}}l_{h_{\sigma }}}\right)}$

$$≲\sqrt{{𝒞}_{1}^{H_{n}}}\text{exp}\left\{H_{n}\text{log}\left(\frac{C_{1}^{\text{*}}}{\epsilon_{1}}\right)+\frac{H_{n}}{2}\left(KD^{2}+D(D-1)\right)\text{log}\left(\frac{1}{\epsilon_{1}}\right)\right\}\;\times \text{exp}\left\{\frac{Cn\epsilon^{2}}{2}\left(D+\frac{1}{2c_{2}}KD^{2}(T-1+TD)\right)\right\}\times n^{KH_{n}^{3}\left(D^{2}(T-2)+\frac{1}{2}D^{2}-r\right)-KH_{n}^{3}}\;\times \{\prod_{k=1}^{K}\prod_{h_{1k}\leq H_{n}}(j_{h_{1k}}{)}^{D^{2}(T-2)+\frac{1}{2}\left(D^{2}-1\right)}\}\left\{\prod_{k=1}^{K}\prod_{h_{1k}\leq H_{n}}{\left(j_{h_{1k}}-1\right)}^{-1\left(j_{h_{1k}}\geq 2\right)(r+1)}\right\}\;\times \prod_{h_{\sigma }\leq H_{n}}{\left\{n^{l_{h_{\sigma }}}\right\}}^{\frac{D(D-1)}{4}+KD^{2}H_{n}(T-1)\frac{D-1}{4}}\times \left\{\prod_{h_{\sigma }\leq H_{n}}{\left(n^{\kappa \left(l_{h_{\sigma }}-1\right)/2}\right)}^{-1\left(l_{h_{\sigma }}\geq 1\right)}\right\}.$$

Using the above expressions and similar arguments as in (38), it is straightforward to show that condition (2*B*) in Theorem 2 holds. Hence the result is proved.

The proof for the rgPDPM-VAR proceeds in a similar fashion by noting that the prior probability for the complement of the sieves defined in the manuscript can be written as

$$\text{Π}\left({ℱ}_{n}^{c}\right)\leq H_{n}P_{2}^{\text{*}}\left(\lambda_{1}(\text{Σ})>{\underline{\sigma }}_{n}^{2}{\left(1+\epsilon /\sqrt{D}\right)}^{M_{n}}\right)+H_{n}P_{2}^{\text{*}}\left(\frac{\lambda_{1}\left({\text{Σ}}_{h_{\sigma }}\right)}{\lambda_{D}\left({\text{Σ}}_{h_{\sigma }}\right)}>n^{H_{n}}\right)+H_{n}P_{2}^{\text{*}}\left(\lambda_{D}(\text{Σ})\leq {\underline{\sigma }}_{n}^{2}\right)\;+\mathrm{Pr}\left(\sum_{h_{\sigma }>H_{n}}\pi_{\sigma ,h_{\sigma }}>\epsilon_{2}\right)+\mathrm{Pr}\left(\sum_{h_{11}>H_{n}}\pi_{1,h_{11}}^{\text{*}}>\epsilon_{2}\right)+\cdots +\mathrm{Pr}\left(\sum_{h_{1D}>H_{n}}\pi_{D,h_{1D}}^{\text{*}}>\epsilon_{2}\right)≲e^{-b^{\text{*}}n},$$

using similar techniques used to derive (37) in Lemma 6 in the Appendix. Hence, condition (2*A*) in Theorem 2 holds. Also define ${ℱ}_{n,j,l}$ such that ${ℱ}_{n}\subset \cup_{j,1}{ℱ}_{n,j,l}$ and

(35)
$${ℱ}_{n,j,l}=\left\{f_{p}\in {ℱ}_{n}:\text{for}\;h_{11},\ldots ,h_{1D},n^{H_{n}^{2}}\left(j_{h_{1d^{\prime }}}-1\right)\leq \left\|\mathrm{vec}\left(A_{kh_{1k}}\right)\right\|\leq n^{H_{n}^{2}}j_{h_{1d^{\prime }}},\right.\left.d^{\prime }=1,\ldots ,D,\text{and for}\;h_{\sigma }\leq H_{n},\;n^{l_{h_{\sigma }}-1}\leq \frac{\lambda_{1}\left(\Sigma_{h_{\sigma }}\right)}{\lambda_{D}\left(\Sigma_{h_{\sigma }}\right)}\leq n^{l_{h_{\sigma }}}\right\}.$$

Given the entropy bound in (29) and using similar calculations as in Lemma 7 for the PDPM-VAR case, it is possible to show that (2*B*) in Theorem 2 holds. The strong consistency result follows under rgPDPM-VAR once conditions (2*A*)-(2*B*) in Theorem 2 are satisfied.

**Proof of Lemma 2:** For the case with independent double exponential priors involving shrinkage parameter λ, note that $P\left(a^{2}(k,l)\leq \frac{{\left(x^{*}\right)}^{2}}{D^{2}}\right.$, for all $\left.1\leq k,l\leq D\right)\leq P\left(\left\|vec\left(A_{k}\right)\right\|\leq \right.$$\left.x^{*}\right)$. Further for large positive $x^{*}>D$, and denoting $\pi (s|\lambda )=\frac{1}{2}\sqrt{\lambda }exp\left(-\sqrt{\lambda }\frac{s}{2}\right)$

$$P_{1}^{\text{*}}\left(a^{2}(k,l)>\frac{{\left(x^{\text{*}}\right)}^{2}}{D^{2}}\right)=P\left(|a(k,l)|>\frac{\left(x^{\text{*}}\right)}{D}\right)=2\int_{x^{\text{*}}/D}^{\infty }\int \frac{1}{\sqrt{2\pi s}}\text{exp}\left(-\frac{1}{2s}a^{2}\right)\pi (s|\lambda )ds\;da\;\leq 2\int \frac{1}{\sqrt{2\pi }s\left(x^{\text{*}}/D\right)}\text{exp}\left(-\frac{1}{2s}{\left(x^{\text{*}}/D\right)}^{2}\right)\pi (s|\lambda )ds\leq 2\int \frac{1}{\sqrt{2\pi }s}\text{exp}\left(-\frac{1}{2s}{\left(x^{\text{*}}/d\right)}^{2}\right)\pi (s|\lambda )ds\;=2\text{exp}\left(-\lambda \left|x^{\text{*}}/D\right|\right)=2\text{exp}\left(-\lambda x^{\text{*}}/D\right)\leq {\left(x^{\text{*}}/D\right)}^{-\lambda }.$$

The above implies that $1-{\left(x^{*}/D\right)}^{-\lambda }\leq P_{1}^{*}\left(a^{2}(k,l)\leq \frac{{\left(x^{*}\right)}^{2}}{D^{2}}\right)$ and further that (${\left(1-{\left(x^{*}/D\right)}^{-\lambda }\right)}^{D^{2}}\leq P_{1}^{*}\left(a^{2}(k,l)\leq \frac{{\left(x^{*}\right)}^{2}}{D^{2}}\right.$, for all $\left.1\leq k,l\leq D\right)\leq P_{1}^{*}\left(\left\|\mathrm{vec}\left(A_{k}\right)\right\|\leq x^{*}\right)$. This implies that $P_{1}^{*}\left(\left\|\mathrm{vec}\left(A_{k}\right)\right\|>x^{*}\right)>1-{\left(1-{\left(x^{*}/D\right)}^{-\lambda }\right)}^{D^{2}}$. For large $x^{*}$ and choosing λ large enough, one can use the binomial expansion to write (when D is even)

$$1-{\left(1-{\left(x^{\text{*}}/D\right)}^{-\lambda }\right)}^{D^{2}}=1-\{\left(1-D^{2}{\left(x^{\text{*}}/D\right)}^{-\lambda }\right)+\frac{D^{2}\left(D^{2}-1\right)}{2}{\left(x^{\text{*}}/D\right)}^{-2\lambda }\left(1-\frac{D^{2}-2}{3}{\left(x^{\text{*}}/D\right)}^{-\lambda }\right)+\;\frac{D^{2}\left(D^{2}-1\right)\left(D^{2}-2\right)\left(D^{2}-3\right)}{4!}{\left(x^{\text{*}}/D\right)}^{-4\lambda }\left(1-\frac{D^{2}-4}{5}{\left(x^{\text{*}}/D\right)}^{-\lambda }\right)+\ldots +\;\frac{D^{2}\left(D^{2}-1\right)\ldots \left\{D^{2}-\left(D^{2}-1\right)\right\}}{\left(D^{2}\right)!}{\left(x^{\text{*}}/D\right)}^{-\left(D^{2}\right)\lambda }\}=D^{2}{\left(x^{\text{*}}/D\right)}^{-\lambda }-\kappa^{\text{*}}≲{\left(x^{\text{*}}\right)}^{-\lambda },$$

for large $x^{*}$ and λ such that $1-D^{2}{\left(x^{*}/D\right)}^{-\lambda }>0$ and for some positive constant $\kappa^{*}$. Similar calculations hold for odd D.

Further, when $P_{1}^{*}\left(\mathrm{vec}\left(A_{k}\right)\right)=N_{D^{2}}\left(\mathrm{vec}\left(A_{k}\right);\mu ,\Lambda \right),\Lambda ∼IW\left(\Lambda_{0},\nu_{\lambda }\right)$, the resulting distribution follows a multivariate t-distribution. Hence one can write $P_{1}^{*}\left(\left\|vec\left(A_{k}\right)\right\|>x^{*}\right)\leq$ ${\left(x^{*}\right)}^{\left(\nu_{\lambda }-D^{2}+1\right)/2}$, using arguments similar to those in Canale and De Blasi (2017).

## Appendix B. Additional Lemmas

**Lemma 4:** *The distance between densities* $f_{P_{1}}$ *and* $f_{P_{2}}$ *under the PDPM-VAR can be expressed as*

$${\left\|f_{P_{1}}-f_{P_{2}}\right\|}_{1}\leq 2\epsilon^{2}+\sum_{h_{1},h_{\sigma }<H_{n}}\left|\pi_{h_{1}}^{\left(1\right)}\pi_{\sigma ,h_{\sigma }}^{\left(1\right)}-\pi_{h_{1}}^{\left(2\right)}\pi_{\sigma ,h_{\sigma }}^{\left(2\right)}\right|+4\epsilon \;+\sum_{h_{1},h_{\sigma }\leq H_{n}}\pi_{h_{1}}^{\left(1\right)}\pi_{\sigma ,h_{\sigma }}^{\left(1\right)}{\left\|\prod_{t=1}^{T}\phi_{\Sigma_{h_{\sigma }}^{\left(1\right)}}\left(x_{t}-\sum_{k=1}^{K}A_{k,h_{1}}^{\left(1\right)}x_{t-k}\right)-\prod_{t=1}^{T}\phi_{\Sigma_{h_{\sigma }}^{\left(2\right)}}\left(x_{t}-\sum_{k=1}^{K}A_{k,h_{1}}^{\left(2\right)}x_{t-k}\right)\right\|}_{1}.$$

**Proof:** $\left||f_{P_{1}}-f_{P_{2}}{\|}_{1}=\right.$

$$\|\underset{h_{1},h_{\sigma }\geq 1}{\sum }\pi_{h_{1}}^{\left(1\right)}\pi_{\sigma ,h_{\sigma }}^{\left(1\right)}\overset{T}{\underset{t=1}{\prod }}\phi_{{\text{Σ}}_{h_{\sigma }}^{\left(1\right)}}\left(x_{t}-\overset{K}{\underset{k=1}{\sum }}A_{k,h_{1}}^{\left(1\right)}x_{t-k}\right)-\underset{h_{1},h_{\sigma }\geq 1}{\sum }\pi_{h_{1}}^{\left(2\right)}\pi_{\sigma ,h_{\sigma }}^{\left(2\right)}\overset{T}{\underset{t=1}{\prod }}\phi_{{\text{Σ}}_{h_{\sigma }}^{\left(2\right)}}\left(x_{t}-\overset{K}{\underset{k=1}{\sum }}A_{k,h_{1}}^{\left(2\right)}x_{t-k}\right){\|}_{1}\;=\|\underset{h_{1},h_{\sigma }>H_{n}}{\sum }\pi_{h_{1}}^{\left(1\right)}\pi_{\sigma ,h_{\sigma }}^{\left(1\right)}\overset{T}{\underset{t=1}{\prod }}\phi_{{\text{Σ}}_{h_{\sigma }}^{\left(1\right)}}\left(x_{t}-\overset{K}{\underset{k=1}{\sum }}A_{k,h_{1}}^{\left(1\right)}x_{t-k}\right)-\underset{h_{1},h_{\sigma }>H_{n}}{\sum }\pi_{h_{1}}^{\left(2\right)}\pi_{\sigma ,h_{\sigma }}^{\left(2\right)}\overset{T}{\underset{t=1}{\prod }}\phi_{{\text{Σ}}_{h_{\sigma }}^{\left(2\right)}}\left(x_{t}-\overset{K}{\underset{k=1}{\sum }}A_{k,h_{1}}^{\left(2\right)}x_{t-k}\right)\;+\;\underset{h_{1},h_{\sigma }\leq H_{n}}{\sum }\pi_{h_{1}}^{\left(1\right)}\pi_{\sigma ,h_{\sigma }}^{\left(1\right)}\left\{\overset{T}{\underset{t=1}{\prod }}\phi_{{\text{Σ}}_{h_{\sigma }}^{\left(1\right)}}\left(x_{t}-\overset{K}{\underset{k=1}{\sum }}A_{k,h_{1}}^{\left(1\right)}x_{t-k}\right)-\overset{T}{\underset{t=1}{\prod }}\phi_{{\text{Σ}}_{h_{\sigma }}^{\left(2\right)}}\left(x_{t}-\overset{K}{\underset{k=1}{\sum }}A_{k,h_{1}}^{\left(2\right)}x_{t-k}\right)\right\}\;+\underset{h_{1},h_{\sigma }<H_{n}}{\sum }\left(\pi_{h_{1}}^{\left(1\right)}\pi_{\sigma ,h_{\sigma }}^{\left(1\right)}-\pi_{h_{1}}^{\left(2\right)}\pi_{\sigma ,h_{\sigma }}^{\left(2\right)}\right)\overset{T}{\underset{t=1}{\prod }}\phi_{{\text{Σ}}_{h_{\sigma }}^{\left(2\right)}}\left(x_{t}-\overset{K}{\underset{k=1}{\sum }}A_{k,h_{1}}^{\left(2\right)}x_{t-k}\right)-\underset{j^{\prime }=1,2}{\sum }\{\underset{h_{1}\leq H_{n}}{\sum }\underset{h_{\sigma }>H_{n}}{\sum }\pi_{h_{1}}^{\left(j^{\prime }\right)}\pi_{\sigma ,h_{\sigma }}^{\left(j^{\prime }\right)}\;\overset{T}{\underset{t=1}{\prod }}\phi_{{\text{Σ}}_{h_{\sigma }}^{\left(j^{\prime }\right)}}\left(x_{t}-\overset{K}{\underset{k=1}{\sum }}A_{k,h_{1}}^{\left(j^{\prime }\right)}x_{t-k}\right)+\underset{h_{1}>H_{n}}{\sum }\underset{h_{\sigma }\leq H_{n}}{\sum }\pi_{h_{1}}^{\left(j^{\prime }\right)}\pi_{\sigma ,h_{\sigma }}^{\left(j^{\prime }\right)}\overset{T}{\underset{t=1}{\prod }}\phi_{{\text{Σ}}_{h_{\sigma }}^{(j^{\prime })}}\left(x_{t}-\overset{K}{\underset{k=1}{\sum }}A_{k,h_{1}}^{\left(j^{\prime }\right)}x_{t-k}\right)\}{\|}_{1}.$$

The upper bound for the right hand side of the above equation may be further written as $\sum_{j^{\prime }=1,2}\left\{\sum_{h_{1},h_{\sigma }>H_{n}}\pi_{h_{1}}^{\left(j^{\prime }\right)}\pi_{\sigma ,h_{\sigma }}^{\left(j^{\prime }\right)}{\left\|\prod_{t=1}^{T}\phi_{\Sigma_{h_{\sigma }}^{\left(1\right)}}\left(x_{t}-\sum_{k=1}^{K}A_{k,h_{1}}^{\left(j^{\prime }\right)}x_{t-k}\right)\right\|}_{1}\right\}+\sum_{h_{1},h_{\sigma }\leq H_{n}}\pi_{h_{1}}^{\left(1\right)}\pi_{\sigma ,h_{\sigma }}^{\left(1\right)}\times$${\left\|\prod_{t=1}^{T}\phi_{\Sigma_{h_{\sigma }}^{\left(1\right)}}\left(x_{t}-\sum_{k=1}^{K}A_{k,h_{1}}^{\left(1\right)}x_{t-k}\right)-\prod_{t=1}^{T}\phi_{\Sigma_{h_{\sigma }}^{\left(2\right)}}\left(x_{t}-\sum_{k=1}^{K}A_{k,h_{1}}^{\left(2\right)}x_{t-k}\right)\right\|}_{1}+\sum_{h_{1},h_{\sigma }<H_{n}}\left(\pi_{h_{1}}^{\left(1\right)}\pi_{\sigma ,h_{\sigma }}^{\left(1\right)}-\right.$ $\left.\pi_{h_{1}}^{\left(2\right)}\pi_{\sigma ,h_{\sigma }}^{\left(2\right)}\right){\left\|\prod_{t=1}^{T}\phi_{\Sigma_{h_{\sigma }}^{\left(2\right)}}\left(x_{t}-\sum_{k=1}^{K}A_{k,h_{1}}^{\left(2\right)}x_{t-k}\right)\right\|}_{1}+\sum_{j^{\prime }=1,2}\left\{\sum_{h_{1}\leq H_{n}}\sum_{h_{\sigma }>H_{n}}\pi_{h_{1}}^{\left(j^{\prime }\right)}\pi_{\sigma ,h_{\sigma }}^{\left(j^{\prime }\right)}\times \|\prod_{t=1}^{T}\phi_{\Sigma_{h_{\sigma }}^{\left(j^{\prime }\right)}}\left(x_{t}-\right.\right.\left.\left.\sum_{k=1}^{K}A_{k,h_{1}}^{\left(j^{\prime }\right)}x_{t-k}\right){\|}_{1}+\sum_{h_{1}>H_{n}}\sum_{h_{\sigma }\leq H_{n}}\pi_{h_{1}}^{\left(j^{\prime }\right)}\pi_{\sigma ,h_{\sigma }}^{\left(j^{\prime }\right)}\|\prod_{t=1}^{T}\phi_{\Sigma_{h_{\sigma }}^{\left(j^{\prime }\right)}}\left(x_{t}-\sum_{k=1}^{K}A_{k,h_{1}}^{\left(j^{\prime }\right)}x_{t-k}\right){\|}_{1}\right\}$.

The right hand side of the above can be further bounded as $\sum_{h_{1},h_{\sigma }\leq H_{n}}\pi_{h_{1}}^{\left(1\right)}\pi_{\sigma ,h_{\sigma }}^{\left(1\right)}\|\prod_{t=1}^{T}\phi_{\Sigma_{h_{\sigma }}^{\left(1\right)}}\left(x_{t}-\right.$ $\left.\sum_{k=1}^{K}A_{k,h_{1}}^{\left(1\right)}x_{t-k}\right)-\prod_{t=1}^{T}\phi_{\Sigma_{h_{\sigma }}^{\left(2\right)}}\left(x_{t}-\sum_{k=1}^{K}A_{k,h_{1}}^{\left(2\right)}x_{t-k}\right){\|}_{1}+\left|\sum_{h_{1},h_{\sigma }>H_{n}}\pi_{h_{1}}^{\left(1\right)}\pi_{\sigma ,h_{\sigma }}^{\left(1\right)}+\sum_{h_{1},h_{\sigma }>H_{n}}\pi_{h_{1}}^{\left(2\right)}\pi_{\sigma ,h_{\sigma }}^{\left(2\right)}\right|+$ $\sum_{h_{1},h_{\sigma }<H_{n}}\left|\pi_{h_{1}}^{\left(1\right)}\pi_{\sigma ,h_{\sigma }}^{\left(1\right)}-\pi_{h_{1}}^{\left(2\right)}\pi_{\sigma ,h_{\sigma }}^{\left(2\right)}\right|+\sum_{j^{\prime }=1,2}\left\{\sum_{h_{1}\leq H_{n}}\sum_{h_{\sigma }>H_{n}}\pi_{h_{1}}^{\left(j^{\prime }\right)}\pi_{\sigma ,h_{\sigma }}^{\left(j^{\prime }\right)}+\sum_{h_{1}>H_{n}}\sum_{h_{\sigma }\leq H_{n}}\pi_{h_{1}}^{\left(j^{\prime }\right)}\pi_{\sigma ,h_{\sigma }}^{\left(j^{\prime }\right)}\right\}$ $\leq \sum_{h_{1},h_{\sigma }\leq H_{n}}\pi_{h_{1}}^{\left(1\right)}\pi_{\sigma ,h_{\sigma }}^{\left(1\right)}{\prod_{t=1}^{T}\phi_{\Sigma_{h_{\sigma }}^{\left(1\right)}}\left(x_{t}-\sum_{k=1}^{K}A_{k,h_{1}}^{\left(1\right)}x_{t-k}\right)-\prod_{t=1}^{T}\phi_{\Sigma_{h_{\sigma }}^{\left(2\right)}}\left(x_{t}-\sum_{k=1}^{K}A_{k,h_{1}}^{\left(2\right)}x_{t-k}\right)}_{1}$ $+2\epsilon^{2}+\sum_{h_{1},h_{\sigma }<H_{n}}\left|\pi_{h_{1}}^{\left(1\right)}\pi_{\sigma ,h_{\sigma }}^{\left(1\right)}-\pi_{h_{1}}^{\left(2\right)}\pi_{\sigma ,h_{\sigma }}^{\left(2\right)}\right|+4\epsilon$, using the fact that $\sum_{h_{1}\leq H_{n}}\sum_{h_{\sigma }>H_{n}}\pi_{h_{1}}^{\left(j^{\prime }\right)}\pi_{\sigma ,h_{\sigma }}^{\left(j^{\prime }\right)}=$ $\left(\sum_{h_{1}\leq H_{n}}\pi_{h_{1}}^{\left(j^{\prime }\right)}\right)\left(\sum_{h_{\sigma }>H_{n}}\pi_{\sigma ,h_{\sigma }}^{\left(j^{\prime }\right)}\right)\leq \epsilon$, since $\sum_{h_{1}>H_{n}}\pi_{h_{1}}<\epsilon ,\sum_{h_{\sigma }>H_{n}}\pi_{\sigma ,h_{\sigma }}<\epsilon$.

**Lemma 5:** *The upper bound for* $\sum_{h_{1},h_{\sigma }<H_{n}}\left|\pi_{h_{1}}^{\left(1\right)}\pi_{\sigma ,h_{\sigma }}^{\left(1\right)}-\pi_{h_{1}}^{\left(2\right)}\pi_{\sigma ,h_{\sigma }}^{\left(2\right)}\right|$ *is given by* $\sum_{h_{1},h_{\sigma }<H_{n}}|{\tilde{\pi }}_{h_{1}}^{\left(1\right)}{\tilde{\pi }}_{\sigma ,h_{\sigma }}^{\left(1\right)}-$ $\pi_{h_{1}}^{\left(2\right)}\pi_{\sigma ,h_{\sigma }}^{\left(2\right)}\left|+\left|1-(1-\epsilon {)}^{2}\right|\right.$, *where* $\tilde{\pi }=\frac{\pi_{h}}{\left(1-\sum_{h>H}\pi_{h}\right)}$.

**Proof:** Note that $\sum_{h_{1},h_{\sigma }<H_{n}}\left|\pi_{h_{1}}^{\left(1\right)}\pi_{\sigma ,h_{\sigma }}^{\left(1\right)}-\pi_{h_{1}}^{\left(2\right)}\pi_{\sigma ,h_{\sigma }}^{\left(2\right)}\right|\leq$

(36)
$$\underset{h_{1},h_{\sigma }<H_{n}}{\sum }\left|\pi_{h_{1}}^{\left(1\right)}\pi_{\sigma ,h_{\sigma }}^{\left(1\right)}-\left(1-\underset{h_{1}>H}{\sum }\pi_{h_{1}}^{\left(1\right)}\right)\left(1-\underset{h_{\sigma }>H}{\sum }\pi_{\sigma ,h_{\sigma }}^{\left(2\right)}\right)\pi_{h_{1}}^{\left(2\right)}\pi_{\sigma ,h_{\sigma }}^{\left(2\right)}\right|\;+\underset{h_{1},h_{\sigma }<H_{n}}{\sum }\left|\left((1-\underset{h_{1}>H}{\sum }\pi_{h_{1}}^{\left(1\right)}\right)\left(1-\underset{h_{\sigma }>H}{\sum }\pi_{\sigma ,h_{\sigma }}^{\left(2\right)}\right)\pi_{h_{1}}^{\left(2\right)}\pi_{\sigma ,h_{\sigma }}^{\left(2\right)}-\pi_{h_{1}}^{\left(2\right)}\pi_{\sigma ,h_{\sigma }}^{\left(2\right)}\right|\;=\left(1-\underset{h_{1}>H}{\sum }\pi_{h_{1}}^{\left(1\right)}\right)\left(1-\underset{h_{\sigma }>H}{\sum }\pi_{\sigma ,h_{\sigma }}^{\left(2\right)}\right)\underset{h_{1},h_{\sigma }<H_{n}}{\sum }\left|{\tilde{\pi }}_{h_{1}}^{\left(1\right)}{\tilde{\pi }}_{h_{\sigma }}^{\left(1\right)}-\pi_{h_{1}}^{\left(2\right)}\pi_{\sigma ,h_{\sigma }}^{\left(2\right)}\right|\;+\left|\left(1-\underset{h_{1}>H}{\sum }\pi_{h_{1}}^{\left(1\right)}\right)\left(1-\underset{h_{\sigma }>H}{\sum }\pi_{\sigma ,h_{\sigma }}^{\left(2\right)}\right)-1\right|(\underset{\underset{h_{1},h_{\sigma }<H_{n}}{\sum }}{\sum }\pi_{h_{1}}^{\left(2\right)}\pi_{\sigma ,h_{\sigma }}^{\left(2\right)})\;\leq \underset{h_{1},h_{\sigma }<H_{n}}{\sum }\left|{\tilde{\pi }}_{h_{1}}^{\left(1\right)}{\tilde{\pi }}_{\sigma ,h_{\sigma }}^{\left(1\right)}-\pi_{h_{1}}^{\left(2\right)}\pi_{\sigma ,h_{\sigma }}^{\left(2\right)}\right|+\left|1-{\left(1-\epsilon \right)}^{2}\right|,\text{where}\;\tilde{\pi }=\frac{\pi_{h}}{\left(1-\underset{h>H}{\sum }\pi_{h}\right)}..$$

**Lemma 6:** *For the PDPM-VAR the prior tail condition (2A) in Theorem 2 is satisfied*.

**Proof:** For the PDPM-VAR, the prior probability on the sieve ${ℱ}_{n}$ defined in (30) satisfies

$$\text{Π}\left({ℱ}_{n}^{c}\right)\leq \mathrm{Pr}\left(\sum_{h_{\sigma }>H_{n}}\pi_{\sigma ,h_{\sigma }}>\epsilon \right)+\mathrm{Pr}\left(\sum_{h_{1}>H_{n}}\pi_{h_{1}}>\epsilon \right)+H_{n}P_{2}^{\text{*}}\left(\lambda_{D}(\text{Σ})\leq {\underline{\sigma }}_{n}^{2}\right)+\;H_{n}P_{2}^{\text{*}}\left(\lambda_{1}(\text{Σ})>{\underline{\sigma }}_{n}^{2}{\left(1+\epsilon /\sqrt{D}\right)}^{M_{n}}\right)+H_{n}P_{2}^{\text{*}}\left(\frac{\lambda_{1}\left({\text{Σ}}_{h_{\sigma }}\right)}{\lambda_{D}\left({\text{Σ}}_{h_{\sigma }}\right)}>n^{H_{n}}\right),$$

using the fact that $P\left[(A\cap B\cap C{)}^{c}\right]=P\left[A^{c}\cup B^{c}\cup C^{c}\right]\leq P\left(A^{c}\right)+P\left(B^{c}\right)+P\left(C^{c}\right)$. Using the stick-breaking representation for DP for the first term and the prior tail conditions, and following similar steps as in the proof of Proposition 2 in Shen et al. (2013), one has $\Pi \left({ℱ}_{n}^{c}\right)$

$$≲\sum_{m=1,2}{\left\{\frac{e\alpha_{m}}{H_{n}}\text{log}(1/\epsilon )\right\}}^{H_{n}}+H_{n}\left\{e^{-c_{1}{\underline{\sigma }}_{n}^{-2c_{2}}}+{\underline{\sigma }}_{n}^{-2c_{3}}{\left(1+\epsilon /\sqrt{D}\right)}^{-c_{3}M_{n}}+{\left(n^{\frac{1}{\text{log}\;n}}\right)}^{-\kappa Cn\epsilon^{2}}\right\}\;≲2{\left(Cn\epsilon^{2}/\text{log}(n)\right)}^{-Cn\epsilon^{2}/\text{log}(n)}+\left(Cn\epsilon^{2}/\text{log}(n)\right)\left(e^{-c_{1}n}+n^{c_{3}/c_{2}}{\left(1+\epsilon /\sqrt{D}\right)}^{-c_{3}n}+e^{-\kappa Cn\epsilon^{2}}\right)≲e^{-bn}$$

since $n^{\frac{1}{logn}}=e$ and due to the fact that $\left(Cn\epsilon^{2}/log(n)\right)log\left\{-Cn\epsilon^{2}/log(n)\right\}>Cn\epsilon^{2}$ for large n, where $0<b<min\left\{C\epsilon^{2}/2,c_{1},c_{3}log(1+\epsilon /\sqrt{D}),\kappa C\epsilon^{2}\right\}$. Hence the first condition (2*A*) in Theorem 2 is satisfied.

**Lemma 7:** For the PDPM-VAR, we have $\sum_{j_{h_{1}}\in N}\sum_{1\leq l_{h_{\sigma }\leq H_{n}}}\sqrt{N\left(\epsilon ,{ℱ}_{n,j_{h_{1}}l_{h_{\sigma }}}\right)\Pi \left({ℱ}_{n,j_{h_{1}}l_{h_{\sigma }}}\right)}e^{-(4-c)n\epsilon^{2}}\to 0$ as n→∞ for a suitable choice of constants.

**Proof:** Hence we can write $\sqrt{N\left(\epsilon ,{ℱ}_{n,jl},\|\cdot {\|}_{1}\right)\Pi \left({ℱ}_{n,jl}\right)}$

$$≲\sqrt{{𝒞}_{1}^{H_{n}}{\left(\frac{1}{\epsilon }\right)}^{C_{1}H_{n}}\times {\left\{𝒞{\left(1+o_{n}(1)\right)}^{KD^{2}}\right\}}^{H_{n}}\times {\left(\frac{1}{\epsilon }\right)}^{H_{n}KD^{2}+D(D-1)}}\;\times \exp\left\{\frac{1}{2}\left(DH_{n}\log(M)+H_{n}\log\frac{C_{1}}{\epsilon }\right)\right\}\times n^{H_{n}^{3}\left(KD^{2}(T-2)+\frac{1}{2}KD^{2}\right)+\frac{H_{n}}{c_{2}}\left(KD^{2}(T-1)+TKD^{3}\right)}\;\times \left\{n^{-H_{n}^{3}(r+1)K}\underset{h_{1}\leq H_{n}}{\prod }{\left(j_{h_{1}}-1\right)}^{-1_{\left(j_{h_{1}}\geq 2\right)}K(r+1)}\right\}\times \left\{\underset{h_{1}\leq H_{n}}{\prod }{\left(j_{h1}\right)}^{2KD^{2}(T-2)+K\left(D^{2}-1\right)}\right\}\;\times \{\underset{h_{\sigma }\leq H_{n}}{\prod }(n^{{l_{h_{\sigma }})}^{\frac{D(D-1)}{2}}+KD^{2}H_{n}(T-1)\frac{D-1}{2}}\}\times \{\underset{h_{\sigma }\leq H_{n}}{\prod }{\left(n^{\frac{\kappa }{2}\left(l_{h_{\sigma }}-1\right)}\right)}^{-1}(l_{h_{\sigma }}\geq 1)\}.$$

The above can be simplified further as

(37)
$$\sqrt{{𝒞}_{1}^{H_{n}}}\text{exp}\left\{H_{n}\text{log}\left(\frac{C_{1}}{\epsilon }\right)+\frac{H_{n}}{2}\left(KD^{2}+D(D-1)\right)\text{log}\left(\frac{1}{\epsilon }\right)\right\}\;\times \text{exp}\left\{\frac{1}{2}\left(DH_{n}\text{log}(M)+\frac{H_{n}}{c_{2}}\left(KD^{2}(T-1)+TKD^{3}\right)\text{log}(n)\right)\right\}\times n^{KH_{n}^{3}\left(D^{2}(T-2)+\frac{1}{2}D^{2}-r\right)-KH_{n}^{3}}\;\times \left\{\prod_{h_{1}\leq H_{n}}{\left(j_{h1}\right)}^{KD^{2}(T-2)+\frac{K}{2}\left(D^{2}-1\right)}\right\}\left\{\prod_{h_{1}\leq H_{n}}{\left(j_{h_{1}}-1\right)}^{-1\left(j_{h_{1}}\geq 2\right)K(r+1)}\right\}\;\times \prod_{h_{\sigma }\leq H_{n}}{\left\{n^{l_{h_{\sigma }}}\right\}}^{\frac{D(D-1)}{4}+KD^{2}H_{n}{\left(T-1\right)}^{\frac{D-1}{4}}}\times \left\{\prod_{h_{\sigma }\leq H_{n}}{\left(n^{\kappa \left(l_{h_{\sigma }}-1\right)/2}\right)}^{-1_{\left(l_{h_{\sigma }}\geq 1\right)}}\right\},$$

Note that $n^{KH_{n}^{3}\left(D^{2}(T-2)+\frac{1}{2}D^{2}-r\right)}$ is bounded when $r>D^{2}(T-2)+\frac{1}{2}D^{2}$. Further, looking at the terms involving j in the last line of (37), one can sum over j (for a fixed $h_{1}$) to have

$$\sum_{j_{h_{1}\geq 2}}\left\{{\left(j_{h1}\right)}^{KD^{2}(T-2)+\frac{K}{2}\left(D^{2}-1\right)}{\left(j_{h_{1}}-1\right)}^{-1_{\left(j_{h_{1}}\geq 2\right)}K(r+1)}\right\}\approx \sum_{j_{h_{1}\geq 2}}{\left(j_{h1}\right)}^{KD^{2}(T-2)+\frac{K}{2}\left(D^{2}-1\right)-K(r+1)}=(1+ℬ),$$

where ℬ is a suitable finite constant that does not depend on n when r is large enough such that $r>D^{2}(T-2)+\frac{1}{2}D^{2}$, and where the approximation holds for n large enough.

Similarly, looking at the terms involving l, one can sum over l (for a fixed $h_{\sigma }$) to have $\sum_{l_{h_{\sigma }\geq 1}}\left\{{\left(n^{l_{h_{\sigma }}}\right)}^{\frac{D(D-1)}{4}-(\kappa /2)}\right\}\times \left\{{\left(n^{l_{h_{\sigma }}}\right)}^{KD^{2}H_{n}(T-1)\frac{D-1}{4}}\right\}\leq \sqrt{\sum_{l_{h_{\sigma }\geq 1}}\left\{{\left(n^{l_{h\sigma }}\right)}^{\frac{D(D-1)}{2}-(\kappa /2)}\right\}}\times \sqrt{\sum_{l_{h_{\sigma }\geq 1}}\left\{{\left(n^{l_{h\sigma }}\right)}^{KD^{2}H_{n}(T-1)\frac{D-1}{2}}\right\}}$ where the inequality is using Cauchy-Schwartz, and the first term in the upper bound (denoted by ${ℬ}_{1}$ is finite when κ>D(D−1)/2.

Hence the upper bound on the combined terms in the last two lines in (37) is given by $(1+ℬ{)}^{H_{n}}{\left(1+{ℬ}_{1}\right)}^{H_{n}/2}n^{KH_{n}^{3}\left(D^{2}(T-2)+\frac{1}{2}D^{2}-r\right)-KH_{n}^{3}}\times \prod_{h_{\sigma }\leq H_{n}}\sqrt{\sum_{l_{2\leq h_{\sigma }\leq H_{n}}}\left(n^{{\left.l_{h_{\sigma }}\right)}^{KD^{2}H_{n}(T-1)\frac{D-1}{2}}}\right.}$ $=(1+ℬ{)}^{H_{n}}{\left(1+{ℬ}_{1}\right)}^{H_{n}/2}n^{KH_{n}^{3}\left(D^{2}(T-2)+\frac{1}{2}D^{2}-r\right)}\times \prod_{h_{\sigma }\leq H_{n}}\sqrt{\sum_{l_{2\leq h_{\sigma }\leq H_{n}}}\left\{\frac{\left(n^{{\left.l_{h_{\sigma }}\right)}^{KD^{2}}H_{n}(T-1)\frac{D-1}{2}}\right.}{n^{2KH_{n}^{2}}}\right\}=}$.

The sum within the square root can be simplified as $\sum_{l_{2\leq h_{\sigma }\leq H_{n}}}\left\{\frac{\left(n^{{\left.l_{h_{\sigma }}\right)}^{KD^{2}}H_{n}(T-1)\frac{D-1}{2}}\right.}{n^{2KH_{n}^{2}}}\right\}=$ $\sum_{h_{\sigma }\leq H_{n}}{\left(n^{KD^{2}H_{n}(T-1)\frac{D-1}{2}-2KH_{n}^{2}}\right)}^{l_{h_{\sigma }}}=\left(\frac{1-{\left(r^{*}\right)}^{H_{n}}}{1-r^{*}}\right)<1$, where the equality is obtained by summing $H_{n}$ terms in geometric progression, and $r^{*}=n^{KD^{2}H_{n}(T-1)\frac{D-1}{2}-2KH_{n}^{2}}<1$ since $KD^{2}H_{n}(T-1)\frac{D-1}{2}-2KH_{n}^{2}<0$ when n is large enough, and recalling that $1\leq l_{h_{\sigma }}=h_{\sigma }\leq$ $H_{n}$. Combining the above expressions, $\sum_{j_{h_{1}}\in N}\sum_{1\leq l_{h_{\sigma }}\leq H_{n}}\sqrt{N\left(\epsilon ,{ℱ}_{n,j_{h_{1}}l_{h_{\sigma }}}\right)\Pi \left({ℱ}_{n,j_{h_{1}}l_{h_{\sigma }}}\right)}$

(38)
$$≲{\left(1+\max\left\{ℬ,{ℬ}_{1}\right\}\right)}^{H_{n}}n^{KH_{n}^{3}\left(D^{2}(T-2)+\frac{1}{2}D^{2}-r\right)}{\left(\frac{1-{\left(r^{*}\right)}^{H_{n}}}{1-r^{*}}\right)}^{H_{n}}\;\times \exp\left\{H_{n}\log\left(\frac{C_{1}}{\epsilon }\right)+\frac{H_{n}}{2}\left(KD^{2}+D(D-1)\right)\log\left(\frac{1}{\epsilon }\right)\right\}\;\times \exp\left\{\frac{Cn\epsilon^{2}}{2}\left(D+\frac{1}{2c_{2}}KD^{2}(T-1+TD)\right)\right\}≲(\frac{{𝒦}^{*}}{{n^{KH_{n}^{2}\left(r-D^{2}(T-2)+\frac{1}{2}D^{2}\right)})}^{H_{n}}}\;\times \exp\left\{\frac{Cn\epsilon^{2}}{2}\text{\{}\left(D+\frac{1}{2c_{2}}KD^{2}(T-1)\right)+\log\left(\frac{C_{1}}{\epsilon }\right)+\frac{1}{2}\left(KD^{2}+D(D-1)\right)\log\left(\frac{1}{\epsilon }\right)\text{)}\right\}\}$$

where $r^{*}<1,r-D^{2}(T-2)+\frac{1}{2}D^{2}>0,H_{n}log\left(M_{n}\right)=Cn\epsilon^{2}$, and ${𝒦}^{*}>0$ is some finite constant that is a function of K,D,ϵ, and other constants but does not depend on n.

Therefore, $\sum_{j_{h_{1}}\in N}\sum_{1\leq l_{h_{\sigma }\leq H_{n}}}\sqrt{N\left(\epsilon ,{ℱ}_{n,j_{h_{1}}}l_{h_{\sigma }}\right)\Pi \left({ℱ}_{n,j_{h_{1}}l_{h_{\sigma }}}\right)}e^{-(4-c)n\epsilon^{2}}\to 0$ as n→∞ for a suitable choice of C such that $\frac{1}{2}\left(D+\frac{1}{2c_{2}}KD^{2}(T-1)\right)C<1$. Hence the condition (2*B*) in Theorem 2 is satisfied and the strong posterior consistency result is proved corresponding to the PDPM-VAR model.

## Appendix C. Posterior Computation Steps

### C.1 Residual Covariance Updates:

Under the low rank representation, we impose DP mixture priors on ($\Gamma_{i}^{*},\Xi_{i},\Psi_{i}$) leading to a mixture prior on $\Sigma_{i}$. This corresponds to the prior $\left.\Sigma_{i}∼\sum_{h_{\sigma }=1}^{\infty }\pi_{\sigma ,h_{\sigma }}\delta_{\left(\Gamma_{h_{\sigma }}^{*}\right.}^{*},\Xi_{h_{\sigma }},\Psi_{h_{\sigma }}\right)$, where $\left(\Gamma_{h_{\sigma }}^{*},\Xi_{h_{\sigma }},\Psi_{h_{\sigma }}\right)∼P_{2}^{*}\equiv P_{\Gamma^{*}}\times P_{\Xi }\times P_{\Psi }$. Here $P_{\Gamma^{*}}$ is a product of independent standard normal distributions, $P_{\Xi }$ is a product of independent Gamma(1/2,1/2) distributions yielding a half-Cauchy prior on the diagonal elements of Γ and a Cauchy prior on the lower-off-diagonal elements of Γ as in Ghosh and Dunson (2009), and the inverse of the diagonal elements of Ψ have independent $Gamma\left(\alpha_{\sigma },\beta_{\sigma }\right)$ priors. Note that here $\Gamma_{i}^{*}$ is not a square matrix, and by diagonal elements we refer to elements $\Gamma_{i,1,1},\ldots ,\Gamma_{i,B,B}$.

Under the stick-breaking representation of Sethuraman (1994), we can write $\pi_{\sigma ,h_{\sigma }}=$ $\nu_{\sigma ,h_{\sigma }}\prod_{l_{\sigma }=1}^{h_{\sigma }-1}\left(1-\nu_{\sigma ,l_{\sigma }}\right),\nu_{l_{\sigma }}∼Beta\left(1,\alpha_{2}\right)$. We use the slice sampling approach of Walker (2007) to facilitate sampling. This approach introduces a cluster membership indicator, V, with $V_{i}=h_{\sigma }$ when subject i belongs to cluster $h_{\sigma }$, and let ${𝒱}_{h_{\sigma }}=\left\{i:V_{i}=h_{\sigma }\right\}$ be the indices of all subjects belonging to covariance cluster $h_{\sigma }$ and let $n_{\sigma ,h_{\sigma }}$ be the cardinality of this set. Let $g_{i}$ be a uniformly distributed latent variable used to reduce the stick-breaking representation of the DPM to a finite sum. Our sampler updates $\nu_{\sigma ,h_{\sigma }}$, and $g_{i}$ as: $\nu_{\sigma ,h_{\sigma }}\left|\left\{V_{1},\ldots ,V_{N}\right\}∼Beta\left(1+n_{\sigma ,h_{\sigma }},\alpha_{2}+\sum_{i=1}^{n}I_{\left(V_{i}>\nu_{\sigma ,h_{\sigma }}\right)}\right),g_{i}\right|V_{i}∼U\left(0,\pi_{\sigma ,V_{i}}\right)$.

The cluster membership indicators $V_{i}$ are then sampled from a multinomial distribution with posterior probabilities $\left(p\left(V_{i}=1|-\right),\ldots ,p\left(V_{i}=h_{\sigma }^{*}|-\right)\right)$ expressed as, $p\left(V_{i}=h_{\sigma }|-\right)∼\frac{I_{\left(g_{i}<\pi_{\sigma ,h_{\sigma }}\right)}\prod_{t=1}^{T_{i}}\phi_{\Sigma_{h_{\sigma }}}\left(x_{i,t};A_{i1},\ldots ,A_{iK}\right)}{\left.\sum_{h_{\sigma }^{\prime }=1}^{h_{\sigma }^{*}}\{I_{{(g}_{i}<\pi_{\sigma ,h_{\sigma }^{\prime }}})\prod_{t=1}^{T_{i}}\phi_{h_{\sigma }^{\prime }}\left(x_{i,t};A_{i1},\ldots ,A_{iK}\right)\right\}}$, where $h_{\sigma }^{*}=min\left\{h_{\sigma }:g_{i}>1-\sum_{h_{\sigma }^{\prime }=1}^{h_{\sigma }}\pi_{\sigma ,h_{\sigma }}\right.$, for all $\left.i\right\}$. Conditioned on the cluster memberships, it is straightforward to update the variables in the low rank representation of Σ using similar steps as in Ghosh and Dunson (2009). We start by sampling the elements of $\Gamma_{h_{\sigma }^{*}}$ one row at a time from their full conditionals: $\Gamma_{h_{\sigma },d^{\prime }}^{*}|-∼$ $N\left(\mu_{\Gamma_{h_{\sigma },d^{\prime }}^{*}},\Sigma_{\Gamma_{h_{\sigma },d^{\prime }}^{*}}\right)$, where $\Sigma_{\Gamma_{h_{\sigma },d^{\prime }}^{*}}={\left(\sigma_{h_{\sigma },d^{\prime }}^{-2}\sum_{i\in {𝒱}_{h_{\sigma }}}\sum_{t=1}^{T_{i}}{\left({ℰ}_{\eta ,itd^{\prime }}^{*}\right)}^{\prime }\left({ℰ}_{\eta ,itd^{\prime }}^{*}\right)+I_{min\left\{d^{\prime },B\right\}}\right)}^{-1}$, $\mu_{\Gamma_{h_{\sigma },d^{\prime }}^{*}}^{*}=\Sigma_{\Gamma_{h_{\sigma },d^{\prime }}^{*}}\left(\sigma_{h_{\sigma },d^{\prime }}^{-2}\sum_{i\in {𝒱}_{h_{\sigma }}}\sum_{t=1}^{T_{i}}{ℰ}_{\eta ,\mathrm{it}d^{\prime }}^{*}\left[x_{i,t}^{\left(d^{\prime }\right)}-A_{i,d^{\prime },•}z_{i,t}\right]\right),{ℰ}_{\eta ,\mathrm{it}d^{\prime }}^{*}={\left(\eta_{i,t,1}^{*},\ldots ,\eta_{i,t,min\left\{d^{\prime },B\right\}}^{*}\right)}^{\prime }$, $x_{i,t}^{\left(d\right)}$ is the response for the ith subject at the $d^{\prime }\mathrm{th}$ node and tth time point, and $A_{ik,d^{\prime }}^{\prime }•$ is the transpose of the $d^{\prime }\mathrm{th}$ row of $A_{ik},A_{i,d^{\prime }•}$ is the DK×1 vector formed by stacking the $A_{ik,d^{\prime }}^{\prime }•$ across all lags, and $z_{i,t}={\left[x_{i,t-1}^{\prime },\ldots ,x_{i,t-K}^{\prime }\right]}^{\prime }$ is the DK×1 vector of previous outcomes used to predict the outcome at time t, padded with zeros for the case that t−k<1. The conditionals for the remaining terms in the low rank representation for $\Sigma_{i}$ are: $\eta_{i,t}^{*}|-∼$ $N\left(\mu_{\;\eta_{i,t}^{*}},\Sigma_{\;\eta_{i,t}^{*}}\right),\;\xi_{h_{\sigma },b}\left|\;-∼\mathrm{Gamma}\left(\frac{1+N_{h_{\sigma }}}{2},\frac{1}{2}\left[1+\sum_{i\in {𝒱}_{h_{\sigma }}}\sum_{t=1}^{T_{i}}\eta_{i,t,b}^{*2}\right]\right)\right.$, $\sigma_{h_{\sigma },d^{\prime }}^{-2}-∼\mathrm{Gamma}\left(a_{\sigma }+\frac{N_{h_{\sigma }}}{2},b_{\sigma }+\frac{1}{2}\sum_{i\in {𝒱}_{h\sigma }}\sum_{t=1}^{T_{i}}{\left[x_{i,t}^{\left(d^{\prime }\right)}-A_{i,d^{\prime },•}z_{i,t}-\Gamma_{i,d^{\prime }}^{*}\eta_{i,t}^{*}\right]}^{2}\right)$ where $N_{h_{\sigma }}$ is equal to the total number of time points across all subjects in covariance cluster $h_{\sigma }$, $\Sigma \eta_{i,t}^{*}={\left(\Xi_{V_{i}}^{-1}+\Gamma_{V_{i}}^{\prime }\Psi_{V_{i}}\Gamma_{V_{i}}\right)}^{-1}$, and $\mu \;\eta_{i,t}^{*}=\Sigma \;\eta_{i,t}^{*}\Gamma_{V_{i}}^{{*}^{\prime }}\Psi_{V_{i}}\left[x_{i,t}^{\left(d^{\prime }\right)}-A_{i,d^{\prime },•}z_{i,t}\right]$.

### C.2 Autocovariance Parameter Updates

#### C.2.1 Computation Steps for PDPM-VAR

As with the covariance terms, we use the stick-breaking representation (Sethuraman, 1994) of the Dirichlet process to enable posterior computation under the DPM priors. For the autocovariance terms, we can express the prior as, $A_{i}|P_{\Theta }∼P_{\Theta },P_{\Theta }=\sum_{h_{1}=1}^{\infty }\pi_{h_{1}}\delta_{A_{h}}$, where $\pi_{h_{1}}=\nu_{h_{1}}\prod_{l_{1}<\nu_{h_{1}}}\left(1-\nu_{l_{1}}\right),\nu_{h_{1}}∼\mathrm{Beta}\left(1,\alpha_{1}\right)$, and $A_{h}∼P_{1}^{*}$, where $P_{1}^{*}$ is a multivariate normal distribution with mean 0 and variance $diag\left\{\tau^{2}\right\}$. The prior for the individual $\tau^{2}$ terms is given by $p\left(\tau_{k,d^{\prime }}^{2}\right)=\frac{\lambda^{2}}{2}exp\left\{-\frac{1}{2}\lambda^{2}\tau_{k,d^{\prime }}^{2}\right\}$, which implies a double exponential base measure for modeling the autocovariance terms (Park and Casella, 2008). Furthermore, we place a conjugate Gamma(r,δ) hyperprior on $\lambda^{2}$ to facilitate Gibbs sampling. Throughout our applications we fix r=1.0 and δ=2.0, which yield good performance in a wide range of settings. As with the residual covariance, we use the slice sampling approach of Walker (2007) to facilitate sampling from this infinite mixture. Let $H_{1}$ be a cluster membership indicator, where $H_{1,i}=h_{1}$ if subject i belongs to the $h_{1}\mathrm{th}$ autocovariance matrix cluster. Let ${ℋ}_{h_{1}}=\left\{i:H_{1,i}=h_{1}\right\}$ be the indices of all subjects belonging to autocovariance cluster $h_{1}$, with $n_{h_{1}}$ being the cardinality of this set. The sampler proceeds by introducing a latent uniform variable $u_{i}$, relating the cluster memberships to the stick breaking representation of the DPM. The sampler proceeds by iteratively sampling $\nu_{h_{1}}$ and $u_{1,i}$ from their full conditionals, $\nu_{h_{1}}\left|\left\{H_{1,i}\right\}∼\mathrm{Beta}\left(1+n_{h_{1}},\alpha_{1}+\sum_{i=1}^{n}I_{\left(H_{1,i}>h_{1}\right)}\right),u_{1,i}\right|\nu_{h_{1}},H_{1,i}∼U\left(0,\pi_{H_{1,i}}\right)$. The cluster memberships, $H_{1,i}$, are then sampled from a multinomial distribution with posterior probabilities $\left(P\left(H_{1,i}=1|-\right),\ldots ,P\left(H_{1,i}={h_{1}}^{*}|-\right)\right)$ given by: $P\left(H_{1,i}=h_{1}|-\right)=\frac{I_{\left(u_{1,i}<\pi_{h_{1}}\right)}\prod_{t=1}^{T_{i}}\phi_{\Sigma_{V_{i}}}\left(x_{i,t}-\sum_{k=1}^{K}A_{k,h_{1}}x_{i,t-k}\right)}{\sum_{{h_{1}}^{\prime }=1}^{{h_{1}}^{*}}\left\{I_{\left(u_{1,i}<{\pi_{h_{1}}}^{\prime }\right)}\prod_{t=1}^{T_{i}}\phi_{\Sigma_{V_{i}}}\left(x_{i,t}-\sum_{k=1}^{K}A_{k,{h_{1}}^{\prime }}x_{i,t-k}\right)\right\}}$ where ${h_{1}}^{*}=min\left\{h_{1}\right.$ : $u_{1,i}>1-\sum_{h_{1^{\prime }}=1}^{h_{1}}\pi_{A,{h_{1}}^{\prime }}$, for all $\left.i\right\}$.

Conditioned on the cluster memberships, we sample the autocovariance matrices across all lags one outcome at a time. The full conditional for $A_{h_{1},d^{\prime }•}$ is given by $A_{h_{1},d^{\prime }•}|-∼N\left(\mu_{A_{h_{1}},d^{\prime }}^{*},\Sigma_{A_{h_{1}},d^{\prime }}^{*}\right)$ with variance and mean: $\Sigma_{A_{h_{1}},d^{\prime }}^{*}={\left(\sum_{i\in {ℋ}_{h_{1}}}\sum_{t=1}^{T_{i}}\sigma_{i,d^{\prime }}^{-2}z_{i,t}z_{i,t}^{\prime }+\Lambda_{D}^{-1}\right)}^{-1}$, $\mu_{A_{h_{1}},d^{\prime }}^{*}=\Sigma_{A_{h_{1}},d^{\prime }}^{*}\left\{\sum_{i\in {ℋ}_{h_{1}}}\sum_{t=1}^{T_{i}}\left[\sigma_{i,d^{\prime }}^{-2}z_{i,t}\left(x_{i,t}^{\left(d^{\prime }\right)}-\Gamma_{i,d^{\prime }}^{*}\eta_{i,t}^{*}\right)\right]\right\}$, respectively.

Finally, the parameters of the double exponential base measure can be updated using the approach outlined in Park and Casella (2008). The variance term in the base measure can be sampled using $\tau_{k,d^{\prime }}^{-2}∼\mathrm{InverseGaussian}\left(\sqrt{\frac{\lambda^{2}}{CA_{k,d^{\prime }}^{2}}},\lambda^{2}\right)$, for k=1,…,K and $d^{\prime }=$ $1,\ldots ,D^{2}$, where C is the number of clusters. The posterior distribution for the lasso parameter is a gamma distribution, $\lambda^{2}\left|\;\tau^{2}∼\mathrm{Gamma}\left(KD^{2}+r,\delta +\sum_{k=1}^{K}\sum_{d^{\prime }=1}^{D^{2}}\frac{\tau_{k,d^{\prime }}^{2}}{2}\right)\right.$.

#### C.2.2 Computation Steps for rgPDPM-VAR

The rgDPM-VAR requires some modification to the slice sampling approach. In particular, the sampler for the rgDPM-VAR extends the latent terms in the slice sampler along the outcome dimension. Let $H_{1d^{\prime }}$ be the vector of autocovariance cluster indices for outcome $d^{\prime }$, with $H_{1d^{\prime },i}=h_{1,d^{\prime }}$ when subject i belongs to outcome $d^{\prime }$ cluster $h_{1,d^{\prime }}$, and let ${ℋ}_{h_{1,d^{\prime }}}=\left\{i\;:\;H_{1d^{\prime },i}=h_{1,d^{\prime }}\right\}$ be the indices of all subjects belonging to outcome $d^{\prime }$ cluster $h_{1,d^{\prime }}$, with $n_{h_{1,d^{\prime }}}$ being the cardinality of this set. Then we have the following full conditionals: $\nu_{h_{1,d^{\prime }}}|\left\{H_{1d^{\prime },i}\right\}∼\mathrm{Beta}\left(1+n_{d^{\prime },h},\alpha_{1}+\sum_{i=1}^{n}I_{\left(H_{1d^{\prime },i}>h_{1,d^{\prime }}\right)}\right)$, $u_{1d^{\prime },i}|\nu_{h_{1,d^{\prime }}},H_{1d^{\prime },i}∼U\left(0,\pi_{d^{\prime },H_{1d^{\prime },i}}\right)$. The cluster memberships, $H_{1d^{\prime },i}$, are then sampled from a multinomial distribution with posterior probabilities $\left(P\left(H_{1d^{\prime },i}=1|-\right),\ldots ,P\left(H_{1d^{\prime },i}={h_{1,d^{\prime }}}^{*}|-\right)\right)$ given by: $P\left(H_{1d^{\prime },i}=h_{1,d^{\prime }}|-\right)=\frac{I_{\left(u_{1d^{\prime },i}<\pi_{d^{\prime },h_{1,d^{\prime }})}\right)}\overset{T_{i}}{\underset{t=1}{\prod }}\phi_{{\text{Σ}}_{V_{i}}}\left(x_{i,t}^{\left(d^{\prime }\right)}-\overset{K}{\underset{k=1}{\sum }}A_{k,h_{1d^{\prime }}}x_{t-k}\right)}{\overset{{h_{1,d^{\prime }}}^{*}}{\underset{h_{1,d^{\prime }}=1}{\sum }}\left\{I_{(u_{1d^{\prime },i}<\pi_{d^{\prime },h_{1,d^{\prime }}}}\overset{T_{i}}{\underset{t=1}{\prod }}\phi_{{\text{Σ}}_{V_{i}}}\left(x_{i,t}^{\left(d^{\prime }\right)}-\overset{K}{\underset{k=1}{\sum }}A_{k,h_{1,d^{\prime }}}x_{t-k}\right)\right\}}\text{, where}\;{h_{1,d^{\prime }}}^{*}=min\left\{h\;:\;u_{1d^{\prime },i}>1-\sum_{h_{1,d^{\prime }}=1}^{h}\pi_{d^{\prime },h_{1,d^{\prime }}}\right.$, for all $\left.i\right\}$. Conditioned on the cluster memberships, the autocovariance terms can be updated in an identical manner to the PDPM-VAR. When updating the parameters of the double exponential base measure the variance terms can be sampled from inverse Gaussian distributions: $\tau_{k,d^{\prime },d^{*}}^{-2}∼\mathrm{InverseGaussian}\left(\sqrt{\frac{\lambda_{d^{\prime }}^{2}}{C_{d^{\prime }}{\bar{A}}_{k,d^{\prime },d^{*}}^{2}}},\lambda_{d^{\prime }}^{2}\right)$ for $k=1,\ldots ,K,d^{*}=1,\ldots ,D$ and $d^{\prime }=1,\ldots ,D$, where $C_{d^{\prime }}$ is the number of autocovariance clusters for outcome $d^{\prime }$ and $\tau_{k,d^{\prime },d^{*}}^{-2}$ is the variance term corresponding to the $d^{*}\mathrm{th}$ element of the $d^{\prime }\mathrm{th}$ row of $A_{k}$, and ${\overset{‾}{A}}_{k,d^{\prime },d^{*}}$ is the average of element $d^{*}$ of the $d^{\prime }\mathrm{th}$ row of $A_{k}$ across the $C_{d^{\prime }}$ clusters. The outcome-specific lasso parameters have gamma posteriors: $\lambda_{d^{\prime }}^{2}\left|\tau_{k,d^{\prime },d^{*}}^{2}∼Ga\left(DK+r,\delta +\sum_{k=1}^{K}\sum_{d^{*}=1}^{D}\frac{\tau_{k,d^{\prime },d^{*}}^{2}}{2}\right)\right.$ for $d^{\prime }=1,\ldots ,D$.

**Table 3:** Average effective sample size (ESS) for elements of the autocovariance matrix for the three proposed methods for varying number of nodes (D). All ESS terms are based on 1500 burnin iterations followed by 3500 MCMC iterations.

D	PDPM-VAR	lgPDPM-VAR	rgPDPM-VAR
50	1736	3160	2850
100	2329	3072	3011

#### C.2.3 Computation Steps for lgPDPM-VAR

The sampling steps under the lgPDPM-VAR model proceeds in a similar manner as the other variants outlined in the manuscript, and are omitted here for space constraints.

## Appendix D. Robustness and Convergence

We assessed the mixing of the chains for the simulation experiment. We evaluated the effective sample size (ESS) and visually inspected trace plots. Table 3 displays the average ESS, providing a picture of how well the sampler does in general. The table clearly displays that we can achieve good mixing. Trace plots for some selected elements of the autocovariance matrix are displayed in Figure 6. The plots do not show any evidence of poor mixing.
